# Ruthenium(II)–Tris-pyrazolylmethane Complexes Inhibit Cancer Cell Growth by Disrupting Mitochondrial Calcium Homeostasis

**DOI:** 10.1021/acs.jmedchem.2c00722

**Published:** 2022-08-01

**Authors:** Jakub Cervinka, Alberto Gobbo, Lorenzo Biancalana, Lenka Markova, Vojtech Novohradsky, Massimo Guelfi, Stefano Zacchini, Jana Kasparkova, Viktor Brabec, Fabio Marchetti

**Affiliations:** † Czech Academy of Sciences, Institute of Biophysics, Kralovopolska 135, CZ-61265 Brno, Czech Republic; ‡ Faculty of Science, Department of Biochemistry, Masaryk University, Kamenice 5, CZ-62500 Brno, Czech Republic; § Department of Chemistry and Industrial Chemistry, University of Pisa, Via G. Moruzzi 13, I-56124 Pisa, Italy; ∥ Department of Industrial Chemistry “Toso Montanari”, University of Bologna, Viale Risorgimento 4, I-40136 Bologna, Italy; ⊥ Faculty of Science, Department of Biophysics, Palacky University in Olomouc, Slechtitelu 27, CZ-78371 Olomouc, Czech Republic

## Abstract

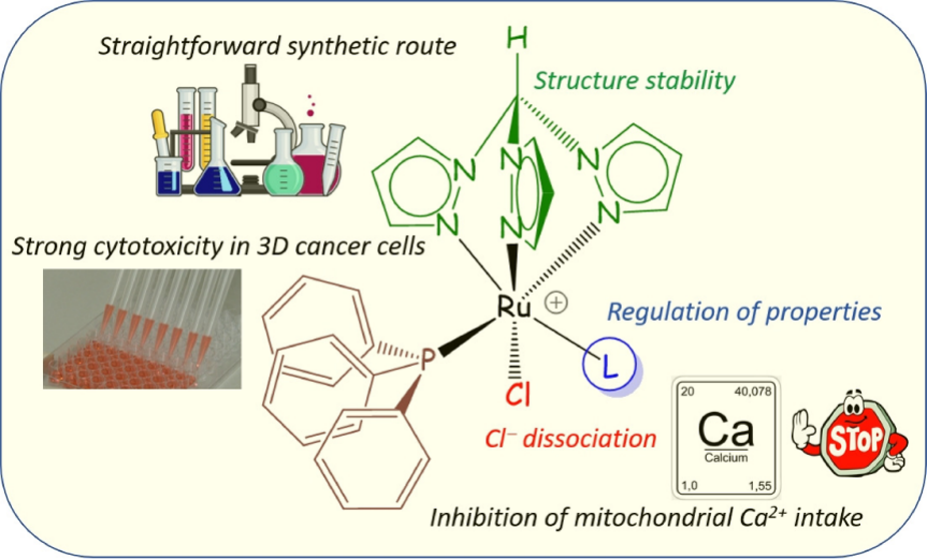

While ruthenium arene complexes have been widely investigated for their medicinal potential, studies on homologous compounds containing a tridentate tris(1-pyrazolyl)methane ligand are almost absent in the literature. Ruthenium(II) complex **1** was obtained by a modified reported procedure; then, the reactions with a series of organic molecules (L) in boiling alcohol afforded novel complexes **2**–**9** in 77–99% yields. Products **2–9** were fully structurally characterized. They are appreciably soluble in water, where they undergo partial chloride/water exchange. The antiproliferative activity was determined using a panel of human cancer cell lines and a noncancerous one, evidencing promising potency of **1**, **7**, and **8** and significant selectivity toward cancer cells. The tested compounds effectively accumulate in cancer cells, and mitochondria represent a significant target of biological action. Most notably, data provide convincing evidence that the mechanism of biological action is mediated by the inhibiting of mitochondrial calcium intake.

## Introduction

**Figure 1:**
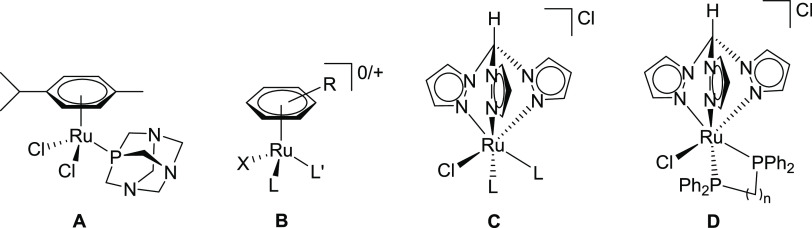
(A) structure of RAPTA-C, leading compound of the RAPTA family; (B) general structure of ruthenium(II)–arene complexes investigated as anticancer drugs (R = alkyl/aryl; X = neutral or ionic ligand; L,L′ = pair of neutral ligands of bidentate neutral/ionic ligand); (C, D) structures of ruthenium–tpm compounds assessed for their cytotoxicity (L = MeCN, DMSO, PMePh_2_; *n* = 2–4).

Complexes of d-block metals possess unique properties otherwise not available to organic compounds and thus offer significant medicinal potential.^1−3^ In particular, platinum compounds have been used in clinical treatments against various types of cancer;^4−6^ however, despite their undisputed efficacy, they exhibit serious drawbacks, such as negative side effects, phenomena of intrinsic or acquired resistance, a limited number of treatable tumors, and the necessity of hospitalization for the intravenous administration.^7−10^ These facts have stimulated research to develop new drugs based on other transition-metal elements.^11−13^ Specifically, a variety of ruthenium complexes have shown great promise.^14,15^ Besides the prototypal NAMI-A, KP1019, and related ruthenium(III) salts that entered clinical trials,^14,16,17^ half-sandwich organometallic complexes based on the Ru^II^–arene scaffold have attracted considerable attention. In particular, RAPTA compounds, featured by the amphiphilic 1,3,5-triaza-7-phosphaadamantane ligand (PTA), have emerged as prominent and are currently pointing to clinical trials.^18,19^ The popularity of RAPTAs and the easy accessibility of related structures have steered the way to the exploration of a considerable number of derivatives with a diversity of arenes and coligands (Figure 1A,B).^20−22^ However, a suitable combination of electronic factors should be formulated to avoid the removal of the arene moiety and the consequent disaggregation of the complexes in aqueous media, which is a disliking characteristic for a drug candidate.^23−27^

Tris(pyrazolyl)methane (tpm) and ring-substituted derivatives are homologous to arenes in that both types of compounds are neutral and may behave as six-electron ligands towards transition-metal centers. However, while the arene–metal bond possesses a π-backbonding component, tpm is essentially a strong donor and provides substantial stability to the resulting complexes.^28,29^ Moreover, tripodal coordination with three nitrogen atoms (κ^3^) is usually observed, although alternative modes are possible,^30,31^ and the interchange between tri- and bidentate coordination might play some key role in metal-catalyzed organic transformations.^32−34^ In sharp contrast with the related arene systems and the fact that NAMI-A and KP1019 contain a mono-pyrazolyl ring, ruthenium(II)–tpm complexes remain almost unexplored for their medicinal potential heretofore. Indeed, to the best of our knowledge, studies are limited to sparse DNA binding experiments^35,36^ and the assessment of the *in vitro* cytotoxicity of complexes [RuCl(κ^3^-tpm)(L)_2_]PF_6_ (L = MeCN, DMSO, PMePh_2_) and [RuCl(κ^3^-tpm)(LL)]PF_6_ (LL= diphosphine) against breast MCF-7 and cervical HeLa cancer cell lines (Figure 1C,D).^37^ In the latter case, while the MeCN and DMSO adducts revealed inactive, the introduction of phosphine ligands resulted in IC_50_ values falling in the low micromolar range. Presumably, the lack of biological studies is also a consequence of the paucity of straightforward routes to add diversity to the {Ru^II^–tpm} scaffold.

Here, we report a general synthetic strategy to access a new family of robust ruthenium(II)–tpm complexes and an extensive investigation of their anticancer activity.

## Results and Discussion

### Synthesis and Structural Characterization of Ruthenium Complexes

**Scheme 1:**
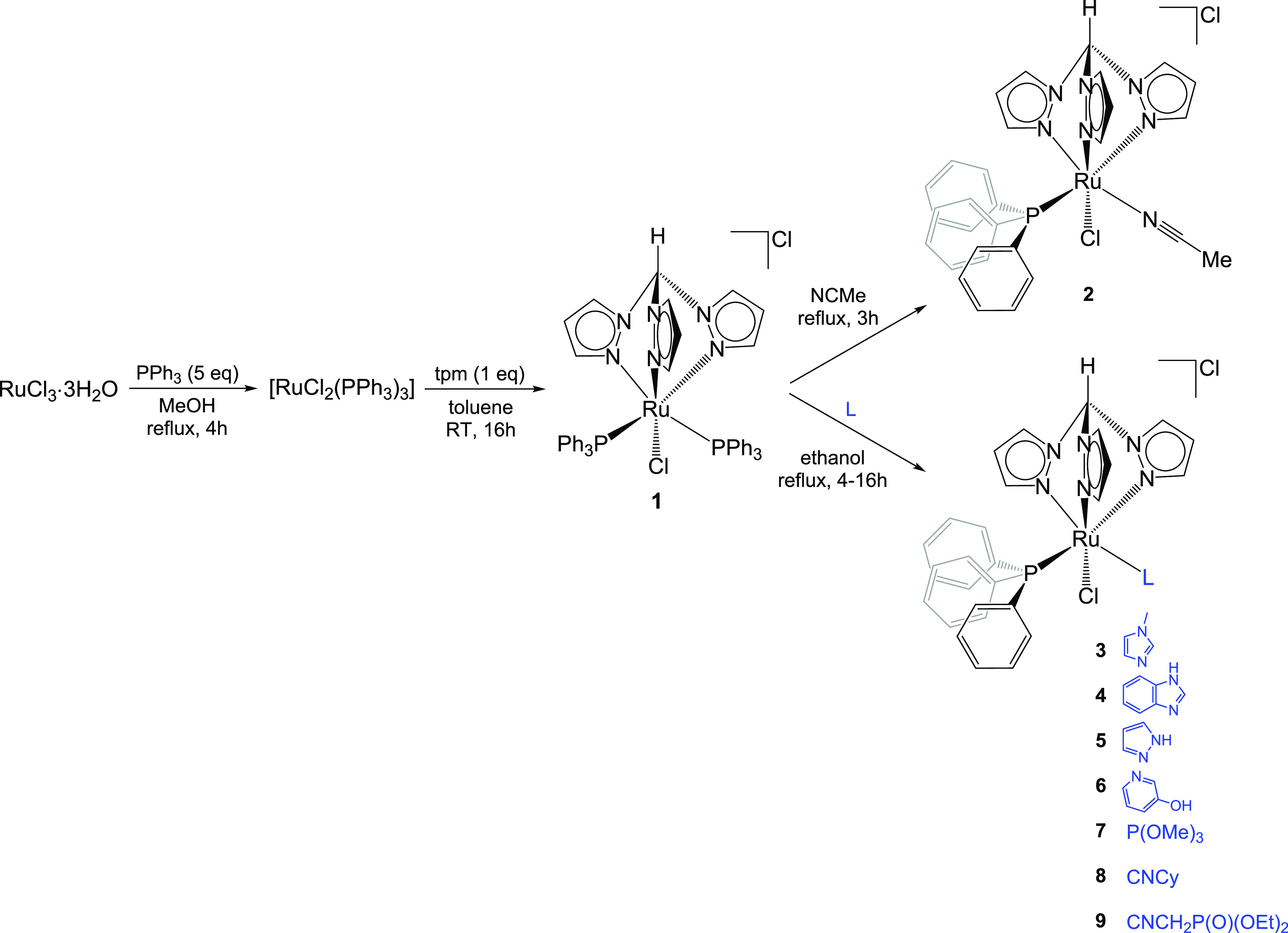
Synthesis of Ruthenium(II) Tris(pyrazolyl)methane Complexes Investigated in This Work (**1–9**); Cy = C_6_H_11_

The ruthenium–tpm complex **1**, containing two triphenylphosphine ligands, was obtained from commercial RuCl_3_·3H_2_O via a straightforward two-step procedure that was optimized with respect to the literature (Scheme 1).^38,39^ In particular, toluene was found to be the optimal solvent for the reaction of tpm with [RuCl_2_(PPh_3_)_3_], allowing to collect the desired product in 95% yield (gram scale). Following a previous work reporting the thermal substitution of one PPh_3_ with PTA (1,3,5-triaza-7-phosphaadamantane),^40^ we investigated the possibility of modifying the ruthenium coordination set in **1** by introducing different types of ligands (i.e., different N-heterocyclic ligands; a phosphite that should provide more hydrophilicity than PPh_3_; two isocyanides with an aryl and alkyl substituent, respectively). Quantitative PPh_3_/MeCN replacement was achieved by heating a solution of **1** in acetonitrile at reflux, affording complex **2** in 95% yield (Scheme 1). Note that nitrile ligands usually behave as labile ones and are easily replaced by phosphines when coordinated to group 8^41−44^ or other transition metals.^45,46^ In the present case, the reversed reaction is probably favored by steric factors arising from two bulky triphenylphosphines occupying adjacent coordination sites in **1**.^38^

To enable other substitution reactions, we found ethanol to be the best solvent; the reactions of **1** with a slight excess of various N-heterocyclic donors, trimethylphosphite and isocyanides, were carried out in ethanol at reflux and proceeded straightforwardly to afford novel complexes **3**–**9**, which were isolated in good to nearly quantitative yields (Scheme 1).

The IR spectra of **4** and **5** (solid state, Figures S1–S9 in the Supporting Information) display the absorption attributed to the NH moiety at ca. 3450 cm^–1^, while the OH group belonging to **6** was detected at 3668 cm^–1^. The intense band due to the carbon–nitrogen triple bond in the isocyanide complexes occurs at 2141 (CNCy, **8**) and 2147 cm^–1^ (CNCH_2_P(O)(OEt)_2_, **9**). These values are almost coincident with those of the respective free isocyanides, indicating a scarce metal to isocyanide backdonation.^47−51^

The NMR spectra of **2–9** (in CDCl_3_, see Figures S10–S35) contain single sets of resonances, and the signals related to tpm are not significantly affected by the nature of the varying ligand (L). More precisely, the ring carbons resonate in the ranges 149.2–144.0 ppm (C^α^), 109.0–107.3 ppm (C^β^), and 136.0–133.4 ppm (C^γ^), whereas the methylidyne group gives rise to resonances at about 12 ppm (^1^H) and 74 ppm (^13^C). Inequivalence of the NMR resonances of the three pyrazolyl rings is in accordance with the chirality of the metal atom. The ^31^P NMR spectra display the resonance related to PPh_3_ falling at 44.8 (**7**)–52.5 (**3**) ppm; the resonance associated with the additional phosphorus ligand in **9** and **7** occurs at 15.7 and 138.0 ppm, respectively.

**Figure 2:**
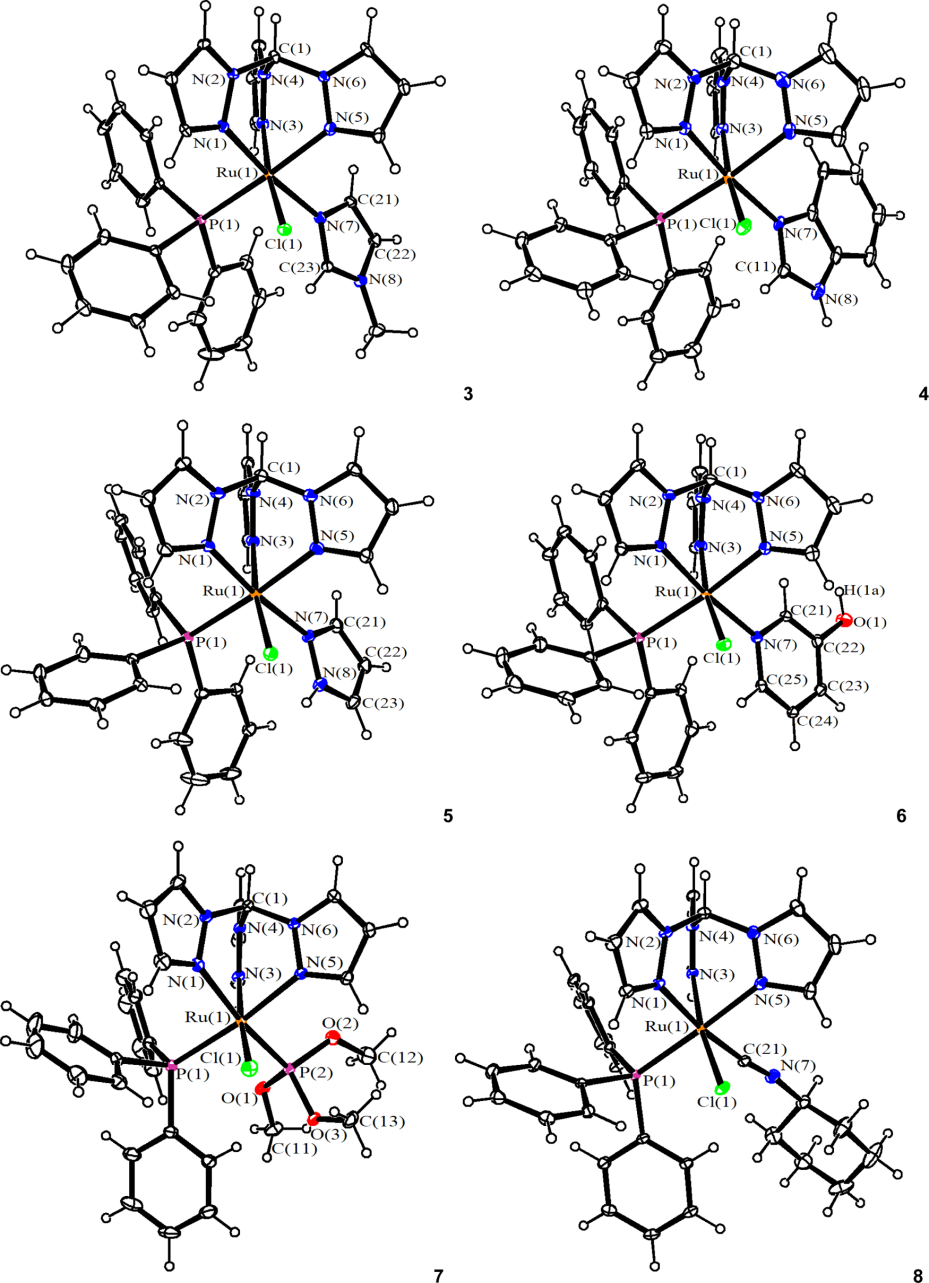
View of the molecular structures (ORTEP drawing) of the cations of **3**, **4**, **5**, **6**, **7**, and **8**. Displacement ellipsoids are at the 30% probability level. CCDC reference numbers are 2167597 (**3**), 2167598 (**4**), 2167599 (**5**), 2167600 (**6**), 2167601 (**7**), and 2167602 (**8**).

The structures of **3**, **4**, **5**, **6**, **7**, and **8** were confirmed by single-crystal X-ray diffraction analyses; views of these structures are shown in Figure 2 with relevant bond lengths and angles reported in Table 1. Complexes **3**–**8** display a distorted octahedral geometry, as found in precursor **1** and related [RuCl(κ^3^–tpm)(PPh_3_)(L)]^+^ complexes.^37,38,52^ The Ru(1)–N(3) and Ru(1)–N(5) distances (Table 1) are comparable in **3–8** since they are trans to Cl(1) and P(1), respectively, in all complexes. In contrast, the Ru(1)–N(1) bond displays similar values in **3–6** [2.056(4), 2.045(5), 2.070(4), and 2.077(3) Å], being in the *trans* position to an aromatic N(7) ligand, whereas it is slightly elongated in **7** [2.180(2) Å] and **8** [2.1428(19) Å], where better π-acceptors P(OMe)_3_ and CNCy are present in the *trans* position. The nature of the varying ligand, L, slightly affects the Ru(1)–Cl(1) distance, reaching the lowest values in complexes **7** and **8** [2.3950(10) and 2.3942(6) Å, respectively]; the strength of the ruthenium–chloride bond is likely to be correlated with the cytotoxic activity of the complexes (vide infra). The Ru(1)–C(21) bond length in **8** [1.918(2) Å] is the shortest reported for an octahedral Ru^II^ center bonded to CNXyl, for which typical Ru–C values fall in the range 1.95–2.04 Å.^53−56^ This is likely to be since CNXyl is *trans* to N(1) in **8**.

**Table 1:** Selected Bond Lengths (Å) and Angles (°) for **3**, **4**, **5**, **6**, **7**, and **8**

	**3**	**4**	**5**	**6**	**7**	**8**
Ru(1)–N(1)	2.056(4)	2.045(5)	2.070(4)	2.077(3)	2.180(2)	2.1428(19)
Ru(1)–N(3)	2.060(4)	2.055(5)	2.069(4)	2.061(3)	2.085(2)	2.0798(17)
Ru(1)–N(5)	2.103(4)	2.106(5)	2.104(4)	2.100(3)	2.131(2)	2.106(2)
Ru(1)–P(1)	2.3063(13)	2.3021(16)	2.3252(13)	2.3184(8)	2.3209(9)	2.3125(6)
Ru(1)–Cl(1)	2.3980(13)	2.4045(15)	2.4252(12)	2.4021(8)	2.3950(10)	2.3942(6)
Ru(1)–X^a^	2.076(4)	2.103(5)	2.082(4)	2.094(3)	2.2181(10)	1.918(2)
N(1)–Ru(1)–N(3)	87.66(17)	87.71(18)	87.68(16)	87.29(11)	87.81(8)	87.79(7)
N(1)–Ru(1)–N(5)	84.36(17)	84.39(19)	85.02(17)	84.69(10)	81.02(8)	82.67(7)
N(3)–Ru(1)–N(5)	84.23(17)	84.1(2)	83.50(17)	84.15(11)	82.88(9)	82.06(7)
P(1)–Ru(1)–Cl(1)	95.74(5)	98.16(6)	96.68(4)	97.75(3)	91.04(3)	92.98(2)
P(1)–Ru(1)–X^a^	94.14(12)	95.35(14)	94.84(12)	95.51(8)	93.35(3)	92.44(7)
Cl(1)–Ru(1)–X^a^	88.62(12)	87.92(14)	87.37(12)	88.03(8)	96.29(3)	91.20(6)

a X = N(7), **3**–**6**; P(2), **7**; C(21), **8**.

The ligands of complexes **4**, **5**, and **6** contain NH (**4** and **5**) or OH (**6**) groups involved in hydrogen bonds. In particular, the N(8)H(8) benzimidazole group of **4** forms a hydrogen bond with the chloride counterion Cl(3) [N(8)–H(8) 0.88 Å, H(8)···Cl(3) 2.15 Å, N(8)···Cl(3) 3.027(6) Å, N(8)–H(8)–Cl(3) 173.7°]. Similarly, the O(1)H(1a) group of **6** is involved in a similar H-bond with the counterion [O(1)–H(1a) 0.848 Å, H(1a)···Cl(2) 2.19 Å, O(1)···Cl(2) 3.027(3) Å, O(1)–H(1a)–Cl(2) 175.7°]. In contrast, the N(8)H(8) group of the pyrazole ligand of **5** forms an intramolecular H-bond with the Cl(1) ligand [N(8)–H(8) 0.88 Å, H(8)···Cl(1) 2.525 Å, N(8)···Cl(1) 3.039(5) Å, N(8)–H(8)–Cl(1) 118.2°].

### Solubility, Partition Coefficient, and Stability in Aqueous Media

A detailed study on the behavior of complexes **1–9** in aqueous media was performed: experimental procedures are provided in Experimental Section, and the results are presented in Table 2.

**Table 2:** Solubility in Water (D_2_O), Octanol/Water Partition Coefficient (log *P*_ow_), and Residual Ruthenium Complex in D_2_O (after 48 h) and DMSO-*d*_6_/DMEM-*d* (1:4 v/v, Except 1:3 v/v in the Case of **4**; after 24 h) Solutions Maintained at 37 °C

compound	solubility/10^–3^ mol·L^–1^ (D_2_O, 21°C)^a^	log *P*_ow_	residual complex % in D_2_O^a^^,^^b^	residual complex % in DMEM-*d*/ DMSO-*d*_6_^a^^,^^b^
**1**		1.18 ± 0.05	69^c^	88
**2**	4.5	–0.33 ± 0.07	89	51
**3**	3.4	–0.15 ± 0.03	100	100
**4**	2.4	0.58 ± 0.06	100	93
**5**	3.4	–0.05 ± 0.05	100	98
**6**	1.9	1.11 ± 0.07	100	100
**7**	3.1	–0.02 ± 0.05	100	95
**8**	1.1	0.34 ± 0.01	92	98
**9**	4.6	–0.20 ± 0.05	93	97

a Calculated by ^1^H NMR (Me_2_SO_2_ as the internal standard).

b Sum of chloro (**2–9**) and aquo (**2**^**W**^–**9**^**W**^) complexes.

c DMSO-*d*_6_/D_2_O (4:1 v/v) mixture.

**Scheme 2:**
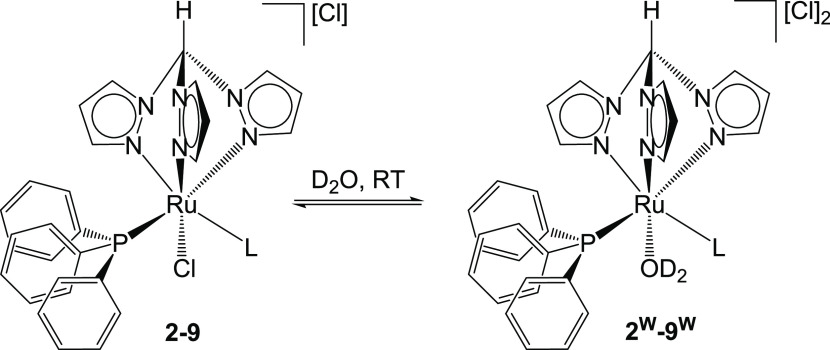
Chloride Dissociation Reaction of Ruthenium(II) Tris(pyrazolyl)methane Complexes in Water

NMR experiments (^1^H and ^31^P) were used to study the speciation of **2**–**9** in D_2_O; preparation of the samples required 2 h (*t*_0_) stirring of a suspension of each complex in D_2_O; then, NMR analyses of the solutions pointed out the occurrence of a chloride–water exchange process (Scheme 2).

NMR data of the resulting dicationic aquo species **2**^**W**^–**9**^**W**^ are reported in the Supporting Information (Figures S36–S43); the representative spectra of **3** dissolved in D_2_O are shown in Figures S44–S45. The aquo complexes were detected in a variable relative amount according to the ligand L (0–90%) after 2 h, as sharp NMR signals; **2**^**W**^–**9**^**W**^ became the largely prevalent species in solution after 48 h in all cases (see below) when the system had probably reached equilibrium. A significant increase of the molar conductivity of a 10^–3^ M solution of **6** in H_2_O was recognized over 24 h, in alinement with the increase by one unit of the net cationic charge of the ruthenium species (see Experimental Section). The pH of a 10^–3^ M H_2_O solution of **3**, this compound undergoing almost complete aquation over 48 h (Table S1), was monitored over time, revealing a substantially constant and close to neutrality value [pH(*t*_0_) = 6.78, pH(_24h_) = 6.66, pH(_48h_) = 6.36]. This evidence reveals that the chloride/water exchange process is not followed by deprotonation of the H_2_O ligand, differently from what is documented for RAPTA compounds.^57^ The chloride/water substitution is fully reversible, in that a mixture of **3** and **3**^**W**^ (isolated from the D_2_O solution) fully reverted to **3** in CD_3_OD during 48 h (see page S32 and Figures S48–S49 in the SI).

Note that leading ruthenium(II)–arene complexes, such as RAPTA compounds, are considered prodrugs, and it has been proposed that the dissociation of chloride ligand(s) in the physiological environment is key to activation, enabling the binding of the metal center with biosubstrates.^18,58−60^ However, the kinetics of chloride/water exchange typically occurs in a much shorter time scale (≤30 min) for the arene complexes.^58−60^

Compound **1** is insoluble in water, and its behavior was evaluated in a DMSO-*d*_6_/D_2_O mixture. Under these conditions, chloride displacement was not recognized, whereas partial substitution of PPh_3_ with one solvent molecule was suggested by the appearance of a new signal at −7.2 ppm in the ^31^P NMR spectrum.

The D_2_O solubility of **2–9** was determined by ^1^H NMR using dimethyl sulfone (Me_2_SO_2_) as the internal standard, ranging between 1.1 and 4.6 mM, with reference to the sum of the chloro and aquo complex observed after 2 h (see above).

The octanol–water partition coefficients (log *P*_ow_) of **1–9** were measured by a UV–vis method; log *P*_ow_ values of **2–9** were obtained approx. 20 min after the dissolution and thus are representative of the monocationic chloro complexes **2**–**9**, as suggested by ^1^H and ^31^P NMR spectra recorded on a D_2_O solution of **3** after the same time (Figures S46–S47). The log *P*_ow_ values are compiled in Table 2 and reflect an overall amphiphilic or moderate lipophilic character. More in detail, **1**, which contains two triphenylphosphine ligands, and **6**, featured by a hydroxy-pyridine ligand, revealed to be the most lipophilic compounds of the series. Conversely, the incorporation of acetonitrile, 1-methylimidazole, pyrazole, trimethylphosphite, and diethyl isocyanomethyl phosphonate as ligands leads to slightly negative log *P*_ow_ values.

The D_2_O solutions of Ru^II^–tpm complexes were then maintained at 37 °C and monitored for 48 h. Apart from the chloride dissociation process, no other changes were observed, except for a minor degradation in the cases of **2**, **8**, and **9**, ascribable to the release of L (see Experimental Section for details). In fact, signals of dissociated PPh_3_ and tpm were not found in the NMR spectra.

The behavior of **1–9** was also analyzed in deuterated cell culture medium. The solutions were diluted with a variable amount of DMSO-*d*_6_ to obtain an appreciable solubility and then kept at 37 °C for 24 h. The compounds were found to be stable even in these conditions, and only minor degradation of the starting materials was detected by ^1^H and ^31^P NMR (1–12%, 49% for **2**). The ^31^P NMR spectra of **1** and **3–9** resembled those recorded in D_2_O–DMSO-*d*_6_ and D_2_O solutions, respectively, while four different signals were detected in the final ^31^P NMR spectrum of **2** (Figure S50). In the case of **2**, in addition to chloride/water exchange, it is possible that replacement of the acetonitrile ligand by solvent molecules occurs. On the other hand, the NMR spectra of **3–9** in DMSO–DMEM after 24 h only contained the resonances related to the starting complexes and the respective aquo species; therefore, DMSO coordination must be ruled out. The presence of ca. 0.1 M chloride ion in the medium slowed down the chloride/water substitution from **1–9**, being almost negligible in the initial solution, and decreased the relative amount of aquo species after 48 h with respect to the analogous experiments in pure D_2_O (see Table S1 in the Supporting Information). The lower percentage of the aquo complex is exhibited by **7**, **8**, and **9**, in accordance with the electron-withdrawing property of trimethylphosphite and isocyanide ligands, presumably strengthening the ruthenium–chloride bond.^61,62^

### Antiproliferative Activity

Primary screening of antiproliferative activity of the family of ruthenium–tpm complexes **1–9** was performed by the commonly used MTT assay on five cancer cell lines of various origins: MCF-7 (breast), HeLa (cervical), 518A2 (melanoma), HCT116 (colon), and RD (rhabdomyosarcoma). In addition, normal human fibroblasts MRC5pd30 were used to assess the toxicity of the complexes on a noncancerous cell line. The results are summarized in Table 3. The IC_50_ values (concentration that causes 50% inhibition of cell proliferation) obtained for **1–9** are compared to those obtained for clinically used cisplatin under the same experimental conditions.

**Table 3:** IC_50_ Values (μM) Determined by the MTT Test after 72 h of Treatment^a^

	MCF-7	HeLa	518A2	HCT116	RD	MRC5pd30	SI^b^
**1**	2.4 ± 0.6	4.0 ± 0.4	2.6 ± 0.4	1.5 ± 0.1	2.2 ± 0.2	5.8 ± 0.7	2.3
**2**	32 ± 4	53 ± 4	26 ± 4	25 ± 2	26 ± 5	76 ± 1	2.4
**3**	38 ± 8	91 ± 3	33 ± 5	25 ± 1	27 ± 4	83.6 ± 0.5	2.0
**4**	37 ± 7	36 ± 1	35 ± 4	25 ± 2	25 ± 3	56.6 ± 0.7	1.8
**5**	32 ± 5	54 ± 6	31 ± 3	30 ± 2	23 ± 2	63 ± 4	1.9
**6**	46 ± 7	54 ± 14	38 ± 8	31 ± 2	38 ± 6	82 ± 4	2.0
**7**	6 ± 1	10 ± 2	6.8 ± 0.8	6.7 ± 0.4	6 ± 1	24 ± 1	3.4
**8**	10 ± 2	15 ± 1	10 ± 2	8 ± 2	6.6 ± 0.7	19.7 ± 0.4	2.0
**9**	43 ± 7	36 ± 2	38 ± 7	35 ± 6	24 ± 3	50.8 ± 0.4	1.5
**cisplatin**	13 ± 3^c^	14 ± 3^c^	2.6 ± 0.7^d^	8 ± 1^c^	4.6 ± 0.3	11.7 ± 0.8^c^	1.1

a The results are expressed as mean values ± SD from at least three independent experiments.

b Selectivity index (SI) was calculated as IC_50_ for noncancerous MRC-5pd30 vs the average IC_50_ value of cancer cell lines.

c Data taken from ref (63).

d Data taken from ref (64).

Most of the investigated compounds possess a moderate activity with IC_50_ values in tens of micromolar. The antiproliferative activity appears significantly influenced by the nature of the ligand (L) and only partially correlated with the log *P*_ow_ values. Thus, among the tested compounds, three complexes stand out. Namely, compound **1** featured the highest degree of lipophilicity within the series (log *P*_ow_ = 1.18) and **7** showed the best antiproliferative activity with IC_50_ values in a single-digit micromolar range in almost all of the investigated cancer cell lines. Furthermore, the potency of these two complexes is comparable to or even better than that of clinically used cisplatin, depending on the specific cell line. On the other hand, the substantially lower activity of **6** (log *P*_ow_ = 1.11), compared to that of **1**, is associated with the presence of the benzimidazole ligand in place of triphenylphosphine.

In addition to **1** and **7**, complex **8** (log *P*_ow_ = 0.34), bearing a cyclohexyl isocyanide ligand, also showed very good activity, still roughly comparable to cisplatin. The beneficial effect of incorporating the cyclohexyl moiety (Cy) within anticancer metal compounds was previously recognized and attributed to its compact and hydrophobic structure.^65−67^

Notably, the cytotoxic effects of **1–9** on noncancerous human skin fibroblasts MRC5pd30 were significantly lower, demonstrating selectivity toward cancer over noncancerous cells.

It should be mentioned that complexes of formula [RuCl(κ*N*-Py)(PPh_3_)(η^6^-*p*-cymene)]^+^ (Py = substituted pyridine) were previously assessed for their cytotoxicity toward the HL60 leukemia tumor cell line, displaying IC_50_ values in the 5–15 μM range.^68^

The MTT assay, employed in the previously described experiment, is based on the mitochondrial reduction of MTT dye to formazan in living cells. However, several ruthenium complexes have been shown to affect mitochondrial metabolism.^69,70^ Moreover, the ruthenium–tris-pyrazolylmethane complexes studied in this work contain triphenylphosphine, which is known to impart specific features and activate additional modes of action to the related metal complexes.^68,71,72^ The data obtained by the MTT assay could then be overestimated due to the possible impact of the investigated compounds on mitochondrial metabolism. Therefore, the experiments focused on antiproliferative activity were repeated using an assay based on a mechanism other than mitochondrial metabolism, namely, Sulphorhodamine B (SRB) assay. This method relies on the stoichiometric binding of SRB dye to proteins in cells. The amount of dye is a proxy for cell mass and thus the number of cells in a sample. For SRB testing (and all further experiments as well), the HCT116 cell line was chosen in which the most active compound **1** exhibited the most promising anticancer activity (the lowest IC_50_ value).

As shown in Table S2 in the Supporting Information, the values obtained by the SRB assay were similar to (in the range of experimental error) or slightly lower than those from the MTT assay. This indicates that mitochondrial succinate dehydrogenase is likely not inhibited by the ruthenium complexes. Notably, the SRB assay confirmed the same trend in the biological activity of all tested complexes as found by MTT, with **1** being the most effective drug, followed by **7** and **8**.

### Intracellular Accumulation

To reveal a possible relationship between the cellular uptake and the *in vitro* activity of the investigated complexes, the ruthenium content of HCT116 cells after 24 h of incubation with complexes **1–9** was determined by inductively coupled plasma mass spectrometry (ICP-MS). The viabilities of the cells after the treatment ranged from 93 to 97%, as verified by the trypan blue exclusion assay so that the results were not affected by elevated permeability of compromised cell membranes of dying/dead cells. The results are summarized in Table 4.

**Table 4:** Accumulation of Ruthenium in HCT116 Cells after Treatment with Ruthenium Complexes (15 μM, 24 h)^a^^,^^b^

	ng Ru/10^6^ cells	log *P*_ow_
**1**	145.1 ± 5.9	1.18 ± 0.05
**2**	29.6 ± 2.9	–0.33 ± 0.07
**3**	66.1 ± 8.0	–0.15 ± 0.03
**4**	54.9 ± 1.3	0.58 ± 0.06
**5**	58.4 ± 3.0	–0.05 ± 0.05
**6**	27.2 ± 1.1	1.11 ± 0.07
**7**	63.3 ± 6.0	–0.02 ± 0.05
**8**	59.4 ± 0.6	0.34 ± 0.01
**9**	16.2 ± 1.4	–0.20 ± 0.05

a log *P*_ow_ values are also reported for comparison.

b Data for intracellular Ru concentration represent the mean ± standard error of the mean (SEM) from two independent experiments.

The inspection of data in Tables 3 and 4 reveals a correlation between antiproliferative activity and intracellular accumulation of the tested ruthenium complexes (Pearson’s correlation coefficient calculated for IC_50_ and accumulated Ru *r* = −0.76, see also Figure S51). This indicates that the ability of individual complexes to cross the cell membrane and accumulate in cells significantly contributes to their respective biological activity. In agreement with this view, the most active complex **1** accumulates in cells much more effectively than the other compounds.

The overall collection of data (speciation in aqueous solutions, log *P*_ow_, average IC_50_, cellular uptake) suggests that the antiproliferative activity of the complexes might be related to adequate lipophilicity of the active species. This condition can be achieved with coligand L (see Scheme 1) providing hydrophobic character and/or disfavoring the conversion into more hydrophilic biscationic complexes via chloride/water exchange (Scheme 2). More precisely, **7** and **8** display log *P*_ow_ values of −0.02 and 0.34, respectively; however the electron-withdrawing character of L limits the elimination of the chloride and the consequent formation of **7**^**W**^–**8**^**W**^ (16% in DMEM-*d*/DMSO-*d*_6_ after 24 h, see Table S1). On the other hand, for instance, **4** and **6** are considerably more lipophilic than **7** and **8**, but the former complexes generate a higher fraction of biscations (**4**^**W**^ and **6**^**W**^, respectively, 59 and 70% in DMEM-*d*/DMSO-*d*_6_), which are expected to be less prone than the parent monocationic complexes to pass through the cell membrane.^73^ Structural factors might also disfavor the penetration of the membrane for [{Ru}–OH_2_]^2+^ species compared to the corresponding [{Ru}–Cl]^+^.

The three most potent compounds, i.e., **1**, **7**, and **8**, were selected for further studies to elucidate their mechanism of action.

### Mechanism of Cell Death Induced by Ru Complexes

**Figure 3:**
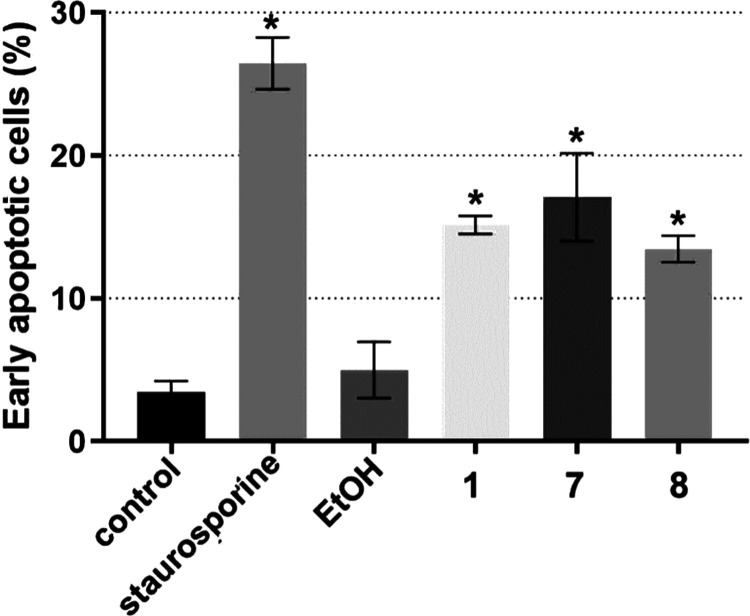
Bar graph of early apoptotic cells (% of the total population) in HCT116 cells treated with Ru complexes **1**, **7**, and **8** for 24 h at their equitoxic concentration (4xIC_50,72h_) quantified by FACS. Positive controls staurosporine (10 μM) and EtOH (5% v/v) were included as well-known apoptosis and necrosis inducers, respectively. Data represent mean ± SEM from three independent measurements; * indicates a significant (*p* < 0.05) difference from control, untreated cells.

The previous test revealed that complexes **1**, **7**, and **8** possess an interesting antiproliferative activity. However, these tests cannot distinguish between the cytostatic (growth arrest and inhibition of division) and cytotoxic (loss of viability) effects.^74,75^ Therefore, we were interested in whether such complexes could induce cell death and, if so, by what mechanism. For this purpose, a commonly used Annexin-V/Propidium iodide (PI) assay was used, and the results were evaluated by flow cytometry (FACS). The typical densitograms obtained from FACS are shown in Figure S52, and Figure 3 shows a quantitative evaluation of the results. All tested complexes effectively induced apoptosis in HCT116 cells (Annexin-V-positive/PI-negative cells), whereas the percentage of cells undergoing necrosis was negligible (0.2–0.3%). This result was confirmed by measuring apoptosis/necrosis in real time immediately after the treatment (Figure S53).

### Intracellular Distribution of Ru

To assess the subcellular distribution of selected complexes (**1**, **7**, and **8**) in HCT116 cells, cell fractionation was carried out following the treatment for 5 and 24 h. A FractionPREP Cell Fractionation kit (BioVision) was employed for the assay. According to the manufacturer’s information, the isolated fractions contain the nucleus (total nucleus soluble proteins, including the nuclear membrane proteins), cytosol (total cellular soluble proteins from cytoplasm), membrane/particulate (total cellular membrane proteins including cellular organelles and organelles membrane proteins), and cytoskeleton + DNA (total cellular insoluble proteins, genomic DNA). In addition, the ruthenium content in each fraction was determined by ICP-MS.

**Figure 4:**
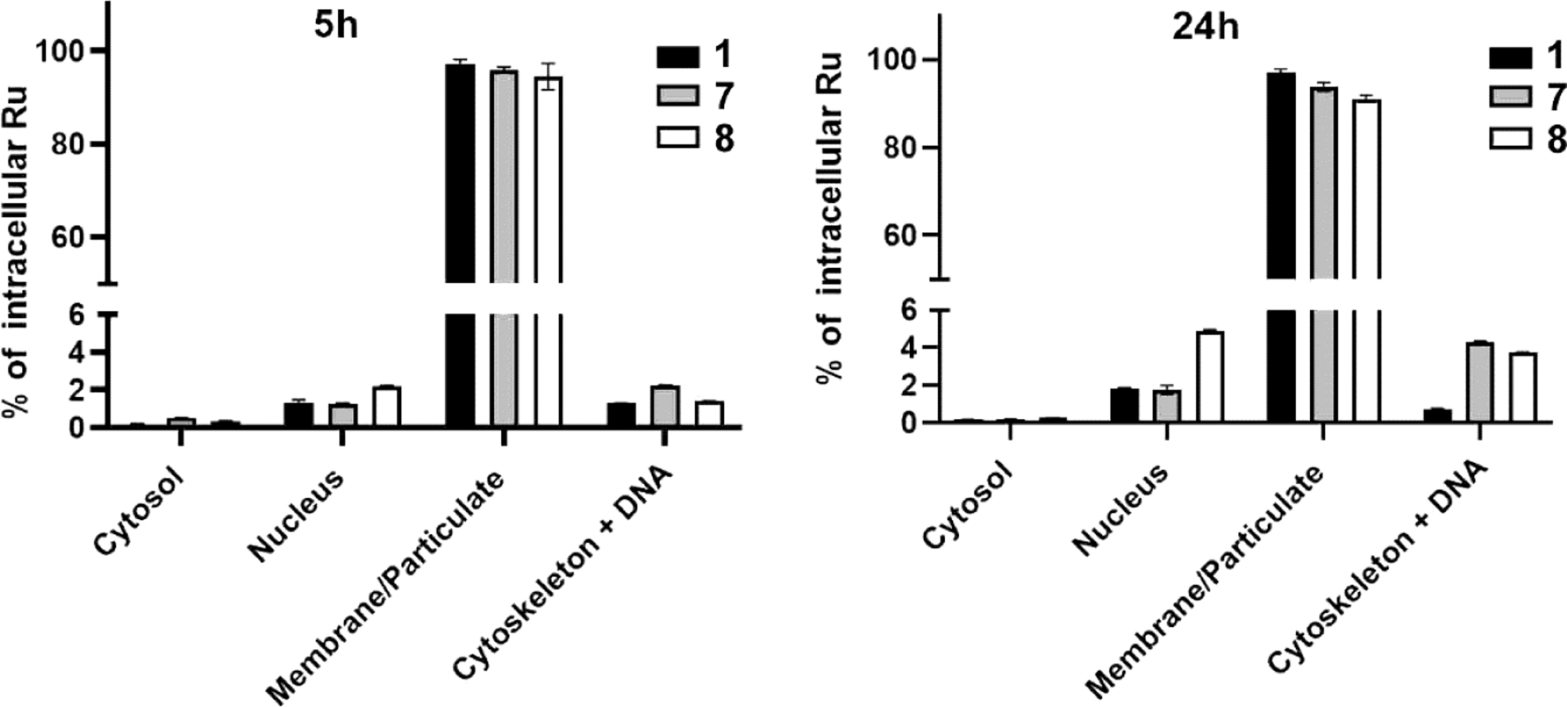
Relative distribution of selected Ru complexes in subcellular fractions after 5 and 24 h treatment of HCT116 cells with equimolar concentrations (10 μM) of **1**, **7**, and **8**. The sum of Ru concentration in all sample compartments was taken as 100%. The experiment was performed in duplicate. Data represents mean ± SEM from two measurements.

As shown in Figure 4, ruthenium from Ru–tpm complexes was detected primarily on the membrane/particulate fraction, so approximately 92–98% of total intracellular Ru was associated with this fraction. This means that the tested complexes preferentially localize in the membrane of organelles like mitochondria or endoplasmic reticulum. However, a small but not insignificant portion of Ru was also associated with nuclear and genomic DNA-containing fractions (2–8%), and the amount of Ru in these fractions increased with incubation time. It has been shown that in the case of cisplatin, whose anticancer mode of action is accepted as mediated by its interaction with DNA,^76^ only ca. 2–3% of intracellular platinum reaches the nucleus and binds to DNA.^77,78^ Thus, although the amount of ruthenium in fractions containing nuclear components (DNA and proteins) is minor, the mechanism of antiproliferative activity via interaction and chromatin damage cannot be excluded.

Additionally, many Ru complexes from the literature have been shown to act via DNA-damaging mechanisms.^79^ The complexes studied in this work contain one chlorido ligand, which is prone to substitution by water in a low-chloride environment such as the intracellular one, thus possibly favoring the DNA binding. Despite not collecting relevant evidence from the stability studies (see Section 2), the additional potential role of κ^3^ to κ^2^ switching of tpm coordination should not be ruled out in principle (see the Introduction). Next, experiments were performed to investigate the possibility of DNA-damaging potency in the overall biological activity of the three leading complexes.

One of the experimental criteria applied to prove DNA binding to be responsible for the cytotoxicity of metal-based drugs is based on the observation that the drug exhibits higher toxicity in the cells deficient in DNA repair.^80,81^ The reason lies in the fact that the ability of DNA lesions to induce cell death is inversely dependent on the capacity of the cells to repair the damage. Therefore, an experiment focused on the effect of the studied complexes in wild-type Chinese hamster ovary cell line CHO-K1 and its mutant NER-deficient counterpart MMC-2 was performed to clarify the involvement of nuclear DNA damage in mediating cellular sensitivity to **1**, **7**, and **8**.

As shown in Table S3, the MMC-2 cells were significantly more sensitive to the treatment with cisplatin (IC_50_ values being almost ten times lower than the IC_50_ values found for parental cells CHO-K1). This result confirms that unrepaired DNA damage caused by cisplatin contributes markedly to its antiproliferative activity, in agreement with the DNA-damage-mediated mechanism of action of cisplatin. However, the IC_50_ values found for selected ruthenium complexes were nearly the same for both MMC-2 and parental CHO-K1 cells, indicating that NER-reparable DNA lesions do not play a significant role in the mechanism of activity of Ru–tpm complexes. Notwithstanding the above results, it should also be noted that cytoskeletal proteins are also present in the DNA-containing fraction. Therefore, to evaluate the possible effect of Ru–tpm complexes on the cytoskeleton, the morphology of cytoskeletal polymers, such as actin and tubulin filaments that participate in many vital cell functions, including division, morphogenesis, phagocytosis, and motility, was monitored by confocal microscopy. As shown in Figures S54 and S55, incubation with complexes **1**, **7**, or **8** did not significantly affect the structure, shape, and layout of either tubulin (Figure S54) or actin (Figure S55) networks, even at concentrations causing a significant antiproliferative effect (IC_50_). Thus, the antiproliferative activity of the investigated complexes seems to be unlikely related to the damage of the two major components of the cellular cytoskeleton, i.e., actin or tubulin filaments.

### Real-Time Cell Growth Monitoring

**Figure 5:**
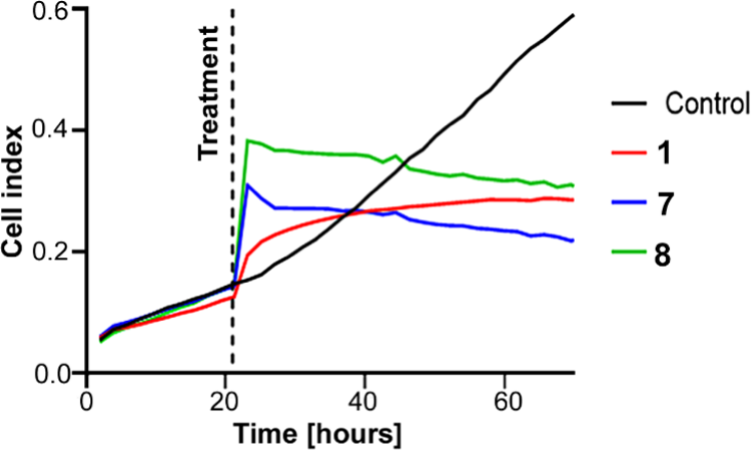
Interaction of HCT116 cells with **1**, **7**, and **8** at their 40, 160, and 160 μM concentrations, respectively, monitored by a real-time cell analyzer (RTCA). The vertical dashed lines indicate the start of the treatment after allowing the cells to adhere to microelectrodes and grow for 24 h. Cell indices were normalized to account for differences in cell counts across the wells prior to the treatment.

The results described above reveal DNA damage as an unlikely cause of the biological action of the studied Ru complexes. Therefore, real-time impedance monitoring of cellular responses was used further to elucidate their mechanism of action. It has been shown that bioactive compounds produce specific time-dependent cell response profiles (TCRPs), predictive of the mechanism of action of the investigated molecules.^82−84^ Furthermore, a comparison of the shape characteristics of the TCRPs obtained for **1**, **7**, and **8** with those published for classes of compounds acting through various mechanisms^82^ revealed that the TRCPs of these three complexes (Figure 5) significantly differ from those characteristic for DNA-damaging agents. This result further supports the view that DNA is not a major target of the Ru–tpm complexes tested in this work.

Interestingly, TRCPs obtained for **1**, **7**, and **8** resemble the profile of deoxycycline,^82^ a known inhibitor of mitochondria in cancer cells. This inhibitor has been shown to impair mitochondrial function by reducing mitochondrial membrane potential and mitochondrial respiration.^85^ Thus, the result of this experiment suggested that the mechanism of action of Ru–tpm complexes could be related to the impairment of mitochondria. Moreover, the majority of the intracellular Ru from **1**, **7**, and **8** was localized in the fraction comprising cellular organelles, including mitochondria (Figure 4). In isolated mitochondria, 467 ± 66, 127 ± 24, and 70 ± 15 pg Ru/10^6^ cells were found when the cells were treated with **1**, **7**, and **8** respectively, indicating that Ru–tpm complexes accumulate in these organelles, although not exclusively. These facts support the hypothesis that the mitochondria may represent one of the significant targets of biological action of the tested complexes. Therefore, several functional assays were used to confirm whether and how the Ru–tpm complexes affect mitochondria in HCT116 cells.

### Effect on Mitochondrial Membrane Potential

To provide evidence for the hypothesis that Ru–tris-pyrazolylmethane complexes tested in this work promote tumor cell death by a mitochondria-dependent mechanism, changes in the mitochondrial membrane potential of HCT116 cells after treatment with **1**, **7**, and **8** were determined by the TMRE assay. TMRE (ethyl ester of tetramethylrhodamine) is a cell-permeant fluorescent dye that accumulates in negatively charged mitochondria in a charge-dependent manner and is therefore used as a marker of mitochondrial membrane potential. If mitochondria depolarize or lose their integrity, the TMRE fluorescence intensity decreases accordingly.

**Figure 6:**
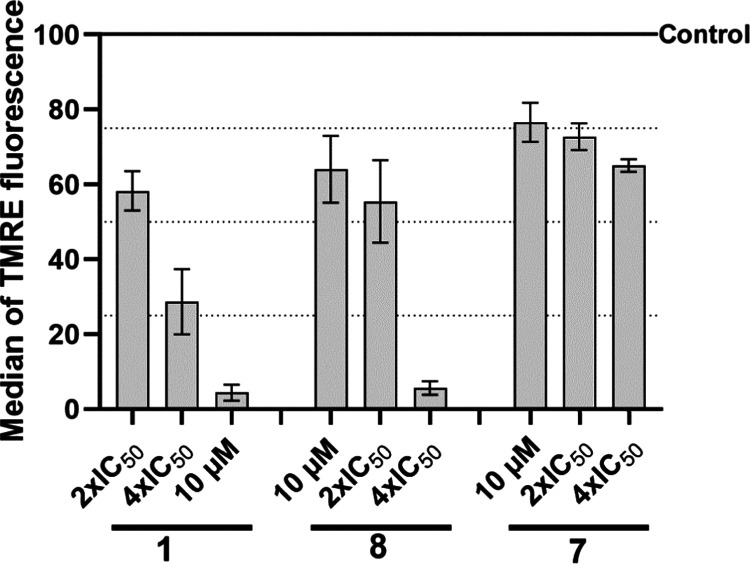
Bar graph showing median of TMRE intensity normalized to the intensity of the control. Cells were treated with equimolar and equitoxic concentrations of Ru complexes. Data represent mean ± SEM from three independent experiments.

After 5 h treatment, the HCT116 cells were stained with TMRE, and fluorescence changes reflecting mitochondrial membrane depolarization in response to the ruthenium complexes were observed by flow cytometry. Quantitative analysis (Figures 6 and S56) revealed a significant decrease of TMRE fluorescence in treated cells compared with control (untreated) cells, which indicated a marked mitochondrial depolarization in HCT116 cells due to the action of ruthenium complexes. The effect was concentration-dependent and more pronounced for **1** and **8**. Instead, **7** was less potent in reducing mitochondrial membrane potential, but its effect was still significant.

### Effect on Oxidative Phosphorylation

Mitochondria are key players in cellular bioenergetics, producing the majority of ATP by oxidative phosphorylation (OXPHOS). Therefore, the next experiment was performed to reveal whether the activity of the Ru complexes toward mitochondria also comprises respiratory chain uncoupling and inhibition of ATP syntheses. Mitochondrial Tox-Glo assay quantifies fluorescence signal originating from cell membrane permeabilization (nonspecific cell death) and luminescence signal generated by luciferase in the presence of ATP. Thus, it can distinguish primary mitochondrial disfunction from secondary cytotoxic events. Cells are treated and grown in a glucose-containing or glucose-free (galactose-supplemented) medium. In the presence of glucose, the cells may preferentially rely on glycolysis to meet bioenergetics needs and are relatively unresponsive to mitochondrial toxins. However, under glucose-free conditions (e.g., in the presence of galactose), the cells necessarily use OXPHOS to generate ATP and are more responsive to mitochondrial perturbation. If the drug disrupts oxidative phosphorylation, then a decrease in the ATP signal becomes observable.

**Figure 7:**
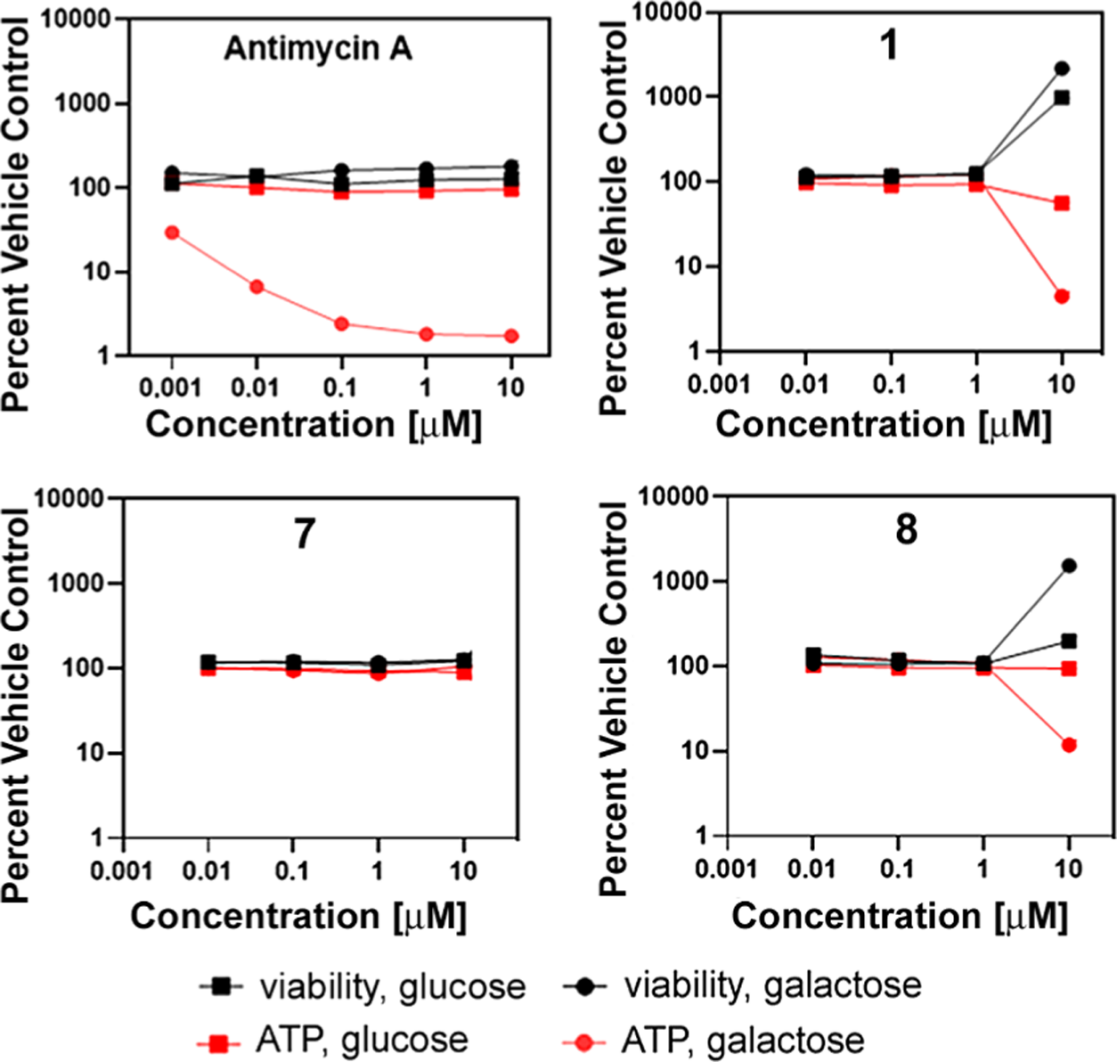
Graphs showing the fluorescence signal intensity (in black, cell viability) and luminescence (in red, ATP) normalized to the untreated control. Cells grown in a glucose-containing medium are marked with a rectangle, while cells grown in a glucose-free medium are marked with a circle. Antimycin A was used as a positive control of OXPHOS inhibition.

As demonstrated in Figure 7, well-known OXPHOS inhibitor Antimycin A (used as a positive control) significantly decreased ATP production by cells growing in galactose-supplemented media. Simultaneously, the viability of cells was unaffected, as demonstrated by no changes in fluorescence signals (Figure 7). This indicates that a decrease in ATP synthesis results from OXPHOS inhibition rather than the overall cytotoxic effect. In contrast, no effect on ATP synthesis was observed for the investigated Ru complexes, neither in glucose-containing nor glucose-free medium. A decrease in ATP production was noticed only at the highest concentrations of complexes **1** and **8**; however, it was accompanied by a decrease in viability. Thus, reducing ATP levels under these conditions likely resulted from the cytotoxic effect instead of OXPHOS inhibition. In summary, the primary mechanism of action of Ru–tris-pyrazolylmethane complexes does not consist in inhibition of OXPHOS; in other words, these compounds do not behave as specific mitotoxicants from the point of view of OXPHOS.

### Effect on Calcium Homeostasis

**Figure 8:**
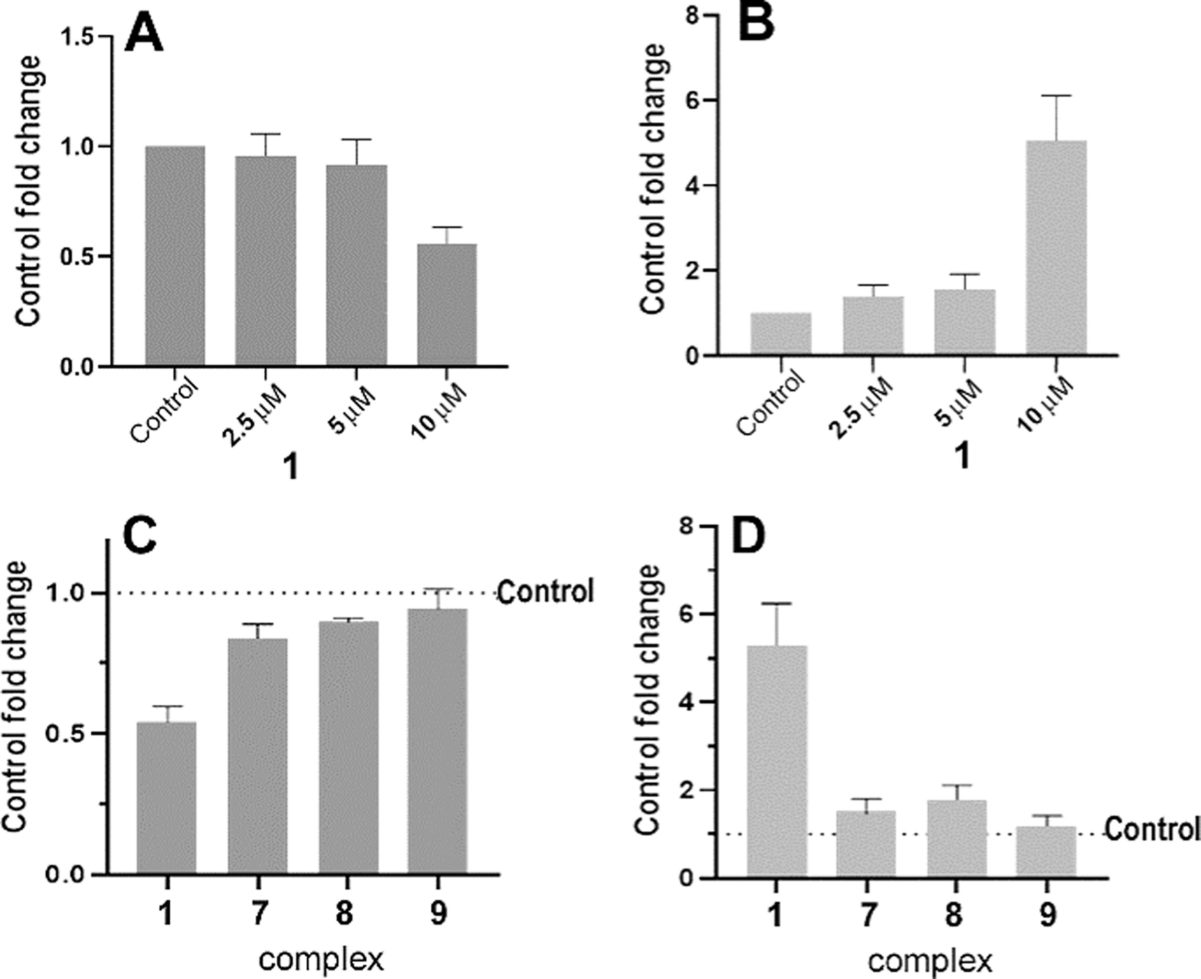
(A, C) Effect on the mitochondrial influx of calcium. Cells pretreated for 2.5 h with 2.5, 5, or 10 μM Ru complex **1** (panel A) or with 10 μM **1**, **7**, **8**, or **9** (panel C) were stained with 5 μM Rhod-2 (mitochondrial Ca^2+^ indicator) and treated with 5 μM ionomycin. (B, D) Effect on the cytosolic concentration of Ca^2+^. Cells were treated for 2.5 h with 2.5, 5, or 10 μM Ru complex **1** (panel B) or with 10 μM **1**, **7**, **8**, or **9** (panel D) and subsequently stained with 5 μM Fluo-4 (cytoplasmic Ca^2+^ indicator). In both experiments, the fluorescence intensity was recorded in PBS with 2 mM CaCl_2_.

The previous results revealed that the investigated ruthenium complexes, although affecting mitochondria, do not uncouple mitochondrial energy metabolism. Thus, another mechanism underlying the effect of the investigated Ru complexes on mitochondria must be in play. Until now, multiple mechanisms of mitochondrial toxicity have been reported besides OXPHOS inhibition.^86,87^ Mitochondria are important in intracellular signal transduction and tuning of calcium (Ca^2+^) homeostasis. In stress conditions, intracellular Ca^2+^ is often elevated, and functional mitochondria serve as a potent Ca^2+^-buffer system.^88^ Much of the function of the mitochondria relies on Ca^2+^ homeostasis and effective Ca^2+^ signaling. Calcium regulates mitochondrial dynamics and functionality, thus affecting various cellular processes, including the mitochondrial pathway, enzyme activity, etc. Then, the fine modulation of mitochondrial calcium homeostasis plays a fundamental role in many processes involving these organelles.^89^ Cancer cells have therefore evolved mechanisms to modulate mitochondrial Ca^2+^ transport in order to sustain their metabolic demand and ensure their survival.^90^ The complex role of mitochondrial calcium in cancer has been thoroughly summarized in several recent reviews.^90−92^ Therefore, we decided to assess the effect of selected complexes on Ca^2+^ homeostasis using cytoplasmic and mitochondrial Ca^2+^ fluorescent sensors. As demonstrated in Figure 8A, complex **1** reduced Ca^2+^ flux into the mitochondria induced by ionomycin, a potent ionophore commonly used for this assay. Similarly, a decrease in Ca^2+^ flux into the mitochondria was also observed for complexes **7**, **8**, and the less active **9** (Figure 8C), although their effects were less pronounced (**7** and **8**) or insignificant (**9**), in agreement with their noticeably lower activity (Table 3). This effect was accompanied by an increase in the concentration of Ca^2+^ in the cytoplasm (Figure 8B,D) and was most prominent for complex **1**, consistently with its greatest antiproliferative activity. The elevation of cytoplasmic calcium can be related to the inhibition of mitochondrial Ca^2+^ intake, as cytoplasmic calcium cannot be transferred to the mitochondria. However, it cannot be ruled out that this increase could also be related to other concurrent factors, such as a release of Ca^2+^ from the endoplasmic reticulum or an effect on Ca^2+^ channels in the cytoplasmic membrane. Thus, the tested complexes behave similarly to other well-known mitochondrial Ca^2+^ uptake inhibitors.^93−97^ The enhanced cytoplasmic concentration of Ca^2+^ ions can then lead to apoptosis via calcineurin-mediated proapoptotic protein activation,^98^ calpain proteases activation,^99^ or autophagy by mTOR inhibition.^100^ These data imply that the mechanism of biological action of the new complexes may also be mediated by disruption of Ca^2+^ homeostasis. On the other hand, in addition to disrupting Ca^2+^ homeostasis, other mechanisms may be involved in the biological effects of the here investigated compounds.

### Cytotoxicity in Three-Dimensional (3D) Spheroids

To emphasize the possibility that the tested complexes might be promising candidates for further preclinical testing and to improve the relevance of our *in vitro* results, we used 3D cell cultures (spheroids), which are much better at replicating in vivo environment than traditional two-dimensional (2D) cultures. Cells grow in complex 3D cultures in an environment closely reflecting the tumor microenvironment, such as nutrient and oxygen gradients, intercellular and cell-extracellular matrix interactions, and heterogeneity.^101^ Hence, 3D growth of immortalized established cell lines or primary cell cultures is regarded as a more stringent and representative model for performing *in vitro* drug screening.^101−103^ Moreover, 3D cell culture models using human cells can circumvent the drawbacks of animal models that, aside from the high cost and ethical considerations, cannot always recapitulate human diseases or capture the side effect of drugs accurately. Also, the tumor spheroids have exhibited several features of the *in vivo* solid tumors. The similarities in the drug responsiveness among the tumor spheroids and the animal models might largely be due to their similarities in enhanced cellular interactions via adhesion and secretion of soluble factors of tumors that lead to low pH and hypoxia.^104^

Therefore, we assessed the ability of **1**, **7**, and **8** to inhibit spheroid formation and growth. HCT116 cells were cultured under 3D cell culture conditions for 96 h to grow up to a tissue mass of around 100 μm in diameter, as described in Experimental Section. The spheroids were then treated with various concentrations of complexes for an additional 72 h. The Cell Titer-Glo 3D cell viability assay was used to determine IC_50_ values. The results (Table 5) confirm that Ru–tpm complexes tested in this experiment, which showed very good activity in conventional 2D cell cultures, also exhibit substantial activity in the 3D spheroids formed from HCT116 cells being significantly more effective than clinically used cisplatin. According to what is observed on 2D cultures, **1** excels in its activity, being approximately 18-fold more effective than cisplatin.

**Table 5:** IC_50_ Values (μM) for the HCT116 Cell Line Determined by the Cell Titer-Glo Test after 72 h of Treatment

compound	HCT116 (3D)
**1**	2.5 ± 0.6
**7**	12.2 ± 2.5
**8**	12.1 ± 2.7
**cisplatin**	44.6 ± 1.8

The effect on the morphology of 3D spheroids of HCT116 cells is shown in Figure S57. The spheroids formed by the control, untreated cells displayed round-shape morphology with a well-defined surrounding edge (Figure S57, panel A). However, after treatment with **7**, **8**, or cisplatin (Figure S57, panels C–E), spheroids displayed heterogeneous morphology with several dissociated cell clumps. Furthermore, these dissociated cell clumps were found to a much greater extent in the samples treated with **1**; under the condition of the experiment, the spheroids treated with this complex were almost completely disintegrated (Figure S57, panel B). Thus, also this morphologic study showed superior activity of Ru–tris-pyrazolylmethane complexes in the 3D spheroids formed from colon cancer HCT116 cells.

## Conclusions

The search for metal drugs alternative to platinum compounds, which are currently administered in clinical treatment against several types of tumors, is an ultimate demand of research. Ruthenium complexes have aroused a great interest in this regard due to their versatile anticancer activity and the scarce toxicity of the metal element, and some ruthenium(II)–arene compounds (RAPTA complexes) are pointing to clinical trials with great promise. Tris-pyrazolylmethane (tpm) is a versatile ligand behaving as a neutral six-electron donor like the arene moiety, but parallel studies on the anticancer potential of Ru–tpm species are lacking in the literature, apparently due to some synthetic drawbacks. Here, we report a straightforward route to access a family of novel, robust cationic ruthenium(II)–tpm complexes differing from each other in one key ligand, modulating both the amphiphilic character and the strength of the ruthenium–chloride bond, which may be implicated in the activation mechanism.

Three complexes of the series, showing a favorable combination of these two factors, displayed micromolar potency against a panel of human cancer cells (comparable to that of conventional cisplatin). Notably, the promising potency of these leading Ru–tpm complexes was bolstered by the results obtained with the 3D spheroids formed from cancer cells, which are much better at replicating in vivo environments than traditional 2D cultures. Moreover, these complexes demonstrated selectivity toward cancer over noncancerous cells. Our further data prove that Ru–tpm complexes effectively induced apoptosis in cancer cells, whereas the percentage of cells undergoing necrosis was negligible. The complexes are taken up in large amounts by cancer cells, and a correlation was observed between antiproliferative activity and intracellular accumulation. *In vitro* growth inhibition studies were completed by investigating the mechanism by which the studied complexes inhibit the growth of cancer cells. The results of these experiments showed that the mitochondria represent a significant target. Although mitochondria are key players in cellular bioenergetics, producing the majority of ATP by oxidative phosphorylation (OXPHOS), the primary mechanism of action of Ru–tpm complexes does not consist of inhibition of OXPHOS. In contrast, we conclude, based on the present findings, that the biological action is mediated by disruption of calcium homeostasis due to the inhibiting of mitochondrial calcium intake. To the best of our knowledge, this is the first study demonstrating a mechanism of antiproliferative activity of ruthenium complexes in cancer cells that involves the regulation of mitochondrial calcium homeostasis. However, the regulation of mitochondrial calcium homeostasis may not be the only mechanism by which the present complexes act. Ruthenium complexes are generally considered to be multifactorial agents, so the biological activity of Ru–tpm complexes may include other factors whose identification was beyond the scope of this work. Nevertheless, disruption of calcium homeostasis undoubtedly contributes to the overall activity of these complexes. Work is currently underway to evaluate other possible mechanistic contributions to the overall biological activity and the in vivo tumor efficacy of this new class of antitumor metal complexes.

## Experimental Section

### General Remarks

Reactants and solvents were purchased from Alfa Aesar, Merck, Strem, or TCI Chemicals and were of the highest purity available. Tris(1-pyrazolyl)methane (tpm) was prepared according to the published procedure.^105^ Reactions were conducted under a N_2_ atmosphere using standard Schlenk techniques, and all products were stored in air once isolated. All compounds are >95% pure by elemental analysis. Solvents were used as received unless otherwise stated. Toluene and diethyl ether were dried with the solvent purification system mBraun MB SPS5, while methanol was distilled from calcium hydride and isopropanol from magnesium. IR spectra of solid samples were recorded on an Agilent Cary630 FTIR spectrometer. IR spectra were processed with Spectragryph software.^106^ NMR spectra were recorded at 298 K on a JEOL JNM-ECZ500R instrument equipped with a Royal HFX Broadband probe. Chemical shifts (expressed in parts per million) are referenced to the residual solvent peaks (^1^H, ^13^C)^107^ or external standard (^31^P to H_3_PO_4_). ^1^H and ^13^C{^1^H} NMR spectra were assigned with the assistance of ^1^H–^13^C (gs-HSQC and gs-HMBC) correlation experiments.^108^ Elemental analyses were performed on a Vario MICRO cube instrument (Elementar). Conductivity measurements were performed at 25 °C using an XS COND 8 instrument (cell constant = 1.0 cm^–1^).^109,110^ pH measurements were performed with an Orion pH meter equipped with a Hamilton glass pH electrode. ESI-Q/ToF flow injection analysis was carried out using a 1200 Infinity HPLC (Agilent Technologies), coupled to a Jet Stream ESI interface (Agilent) with a Quadrupole-Time-of-Flight tandem mass spectrometer 6530 Infinity Q-TOF (Agilent Technologies).

### Synthesis and Characterization of Complexes

#### [RuCl(κ^3^-tpm)(PPh_3_)_2_]Cl, **1** (Chart 1)

**Chart 1:**
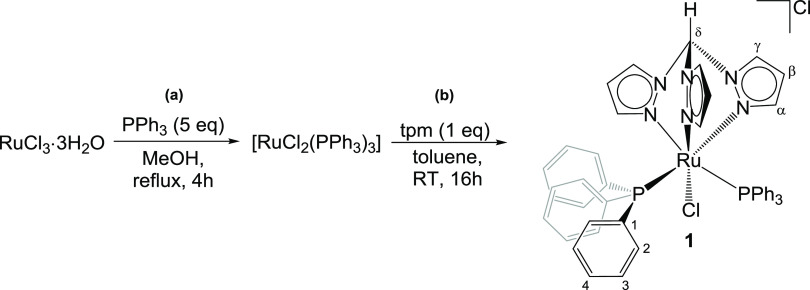
Synthesis and Structure of **1** (Labeling Refers to Carbon Atoms)

The title compound was prepared using a slightly modified literature procedure (Chart 1).^38,39^

#### Step (a): Synthesis of [RuCl_2_(PPh_3_)_3_]

A solution of RuCl_3_·3H_2_O (350 mg, 1.34 mmol) and triphenylphosphine (1.75 g, 6.70 mmol) in distilled methanol (20 mL) was heated at reflux for 4 h. Thus, complex [RuCl_2_(PPh_3_)_3_] precipitated as a brown-purple solid. This solid was separated by filtration under a N_2_ atmosphere, washed with methanol and three times with diethyl ether, and finally dried under vacuum for several hours. The isolated material contained minor amounts of ineliminable PPh_3_ and O=PPh_3_, according to ^31^P NMR (resonances at −5.5 and 29.2 ppm, respectively), which did not affect step (b).

#### Step (b): Synthesis of [RuCl_2_(κ^3^-tpm)(PPh_3_)_2_]

Complex [RuCl_2_(PPh_3_)_3_], as obtained from step (a), and an equimolar amount of tpm (286 mg, 1.34 mmol) were taken in 30 mL of anhydrous toluene under vigorous agitation. A yellow solid rapidly precipitated, and the mixture was left to stir overnight. The solid was filtered, washed with toluene and three times with diethyl ether, and finally dried under vacuum. Yellow solid, yield 1.13 g (95%). Anal. calcd for C_46_H_40_Cl_2_N_6_P_2_Ru: C, 60.66; H, 4.43; N, 9.23; Cl, 7.79. Found: C, 60.35; H, 4.47; N, 9.13; Cl, 7.90. IR (solid state): ṽ/cm^–1^ = 3138w, 3121w, 3101w, 3061w, 3054w, 1515w, 1480m, 1467m, 1454m, 1440m, 1433m, 1411m, 1404m, 1383w, 1303m, 1291m, 1259m, 1254m, 1222m, 1187m, 1090s, 1075m, 1048m, 1024w, 998w, 988w, 852m, 799m, 776m, 768m, 754s, 742s, 696s, 691s, 681m. ^1^H NMR (CDCl_3_): δ/ppm = 12.21 (s, 1H, C^δ^H); 8.86 (d, 1H, ^3^*J*_HH_ = 2.7 Hz, C^γ^H trans to P); 8.64 (d, 2H, ^3^*J*_HH_ = 2.8 Hz, C^γ^H trans to Cl); 7.29–7.26 (m, 3H, C^4^H); 7.15–7.07 (m, 12H, C^3^H + C^2^H); 6.83 (d, 2H, ^3^*J*_HH_ = 2.2 Hz, C^α^H trans to P); 5.98 (t, 2H, ^3^*J*_HH_ = 2.6 Hz, C^β^H trans to P); 5.50 (t, 1H, ^3^*J*_HH_ = 2.7 Hz, C^β^H trans to Cl); 5.12 (d, 1H, ^3^*J*_HH_ = 2.4 Hz, C^α^H trans to Cl). ^31^P{^1^H} NMR (CDCl_3_): δ/ppm = 40.1^38^

#### Preparation of [RuCl(κ^3^-tpm)(PPh_3_)(NCMe)]Cl, **2** (Chart 2)

**Chart 2:**
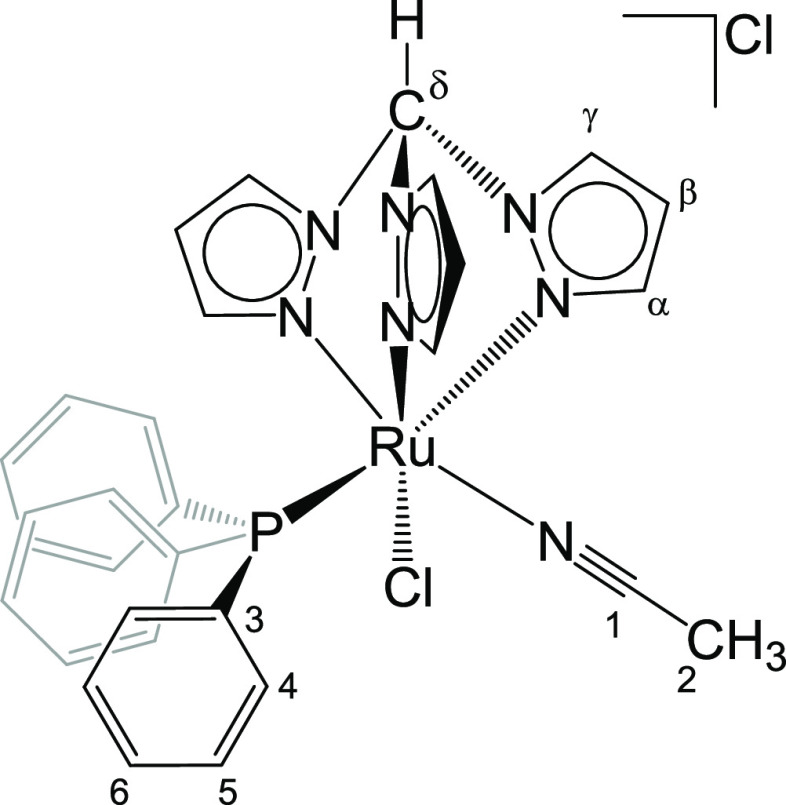
Structure of **2** (Labeling Refers to Carbon Atoms)

A solution of **1** (200 mg, 0.22 mmol) in 25 mL of acetonitrile (MeCN) was heated at reflux for 3 h. The solvent was evaporated under reduced pressure, and the obtained solid was washed with diethyl ether and dried under vacuum. Yellow solid, yield 144 mg (95%). Anal. calcd for C_30_H_28_Cl_2_N_7_PRu: C, 52.26; H, 4.09; N, 14.22; Cl, 10.28. Found: C, 52.08; H, 3.98; N, 14.26; Cl, 10.40. IR (solid state): ṽ/cm^–1^ = 3109w, 3055w, 2958w, 2919w, 2278w (ṽ_N=C_), 1620w-br, 1507w, 1483w, 1450w, 1433m, 1408m, 1375w, 1289m, 1277w, 1252w, 1223w, 1187w, 1090s, 1053w, 1048w, 997w, 987w, 857w, 971s, 779s, 767s, 750s, 756s, 695s. ^1^H NMR (CDCl_3_): δ/ppm = 12.29 (s, 1H, C^δ^H); 8.90, 8.75, 8.71 (d, 3H, ^3^*J*_HH_ = 2.9 Hz, C^γ^H); 8.14 (d-br, 1H, C^α^H); 6.91, 6.55 (d, 2H, ^3^*J*_HH_ = 2.2 Hz, C^α^H); 7.41–7.27 (m, 15H, C^3^H + C^4^H + C^6^H); 6.43 (s-br, 1H C^β^H); 6.07, 5.96 (t, 2H, ^3^*J*_HH_ = 2.6 Hz, C^β^H); 2.16 (s, 3H, C^2^H). ^13^C{^1^H} NMR (CDCl_3_): δ/ppm = 148.0, 147.2, 144.1 (C^α^); 135.6, 135.4, 133.6 (C^γ^); 134.1 (C^4^, ^3^*J*_PC_ = 9.4 Hz); 132.3 (C^3^, ^1^*J*_PC_ = 42.3 Hz); 130.1 (C^6^); 128.3 (C^5^, ^4^*J*_PC_ = 9.3 Hz); 127.9 (C^1^); 108.3, 108.2, 108.0 (C^β^); 74.4 (C^δ^); 4.7 (C^2^). ^31^P{^1^H} NMR (CDCl_3_): δ/ppm = 48.1 (Chart 2).

#### General Procedure for the Synthesis of Complexes [RuCl(κ^3^-tpm)(PPh_3_)(L)]Cl

A solution of **1** and the proper ligand L in ethanol or anhydrous isopropanol was heated at reflux for a variable time. After cooling to room temperature, the volatiles were evaporated under reduced pressure. The crude product was redissolved in the minimum volume of dichloromethane, precipitated with diethyl ether, filtered, and dried under vacuum.

#### [RuCl(κ^3^-tpm)(PPh_3_){NCHN(Me)(CH)_2_}]Cl, **3** (Chart 3)

**Chart 3:**
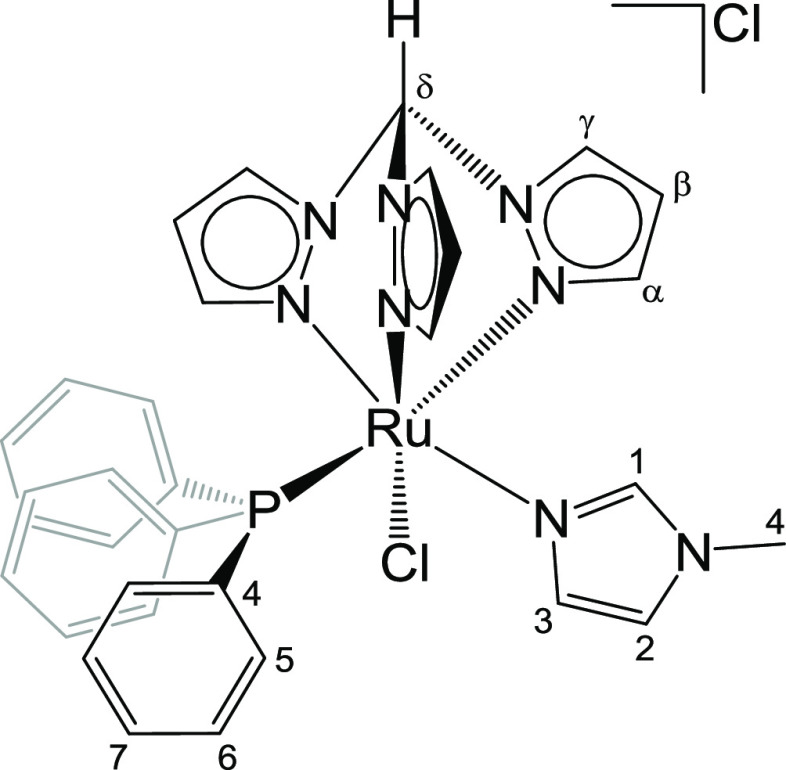
Structure of **3** (Labeling Refers to Carbon Atoms)

From **1** (150 mg, 0.165 mmol) and *N*-methylimidazole (14.5 μL, 0.182 mmol) in ethanol (10 mL). Reaction time: 16 h. Yellow solid, yield 114 mg (94%). Anal. calcd for C_32_H_31_Cl_2_N_8_PRu: C, 52.61; H, 4.28; N, 15.34; Cl, 9.71. Found: C, 52.42; H, 4.17; N, 15.47; Cl, 9.56. IR (solid state): ṽ/cm^–1^ = 3107w-br, 2982w-br, 1481w, 1449w-br, 1434m, 1407m, 1289m, 1250m, 1222w, 1087s, 1053m, 858m, 792s, 755s, 748s-br, 695s. ^1^H NMR (CDCl_3_): δ/ppm = 12.22 (s, 1H, C^δ^H); 8.99, 8.77, 8.73 (d, 3H, ^3^*J*_HH_ = 2.8 Hz, C^γ^H); 7.40, 7.13, 6.72 (d, 3H, ^3^*J*_HH_ = 2.2 Hz, C^α^H); 7.34 (t, 3H, ^3^*J*_HH_ = 7.3 Hz, C^7^H); 7.22–7.18 (m, 7H, C^5^H + CH^Imid^); 7.06 (m, 6H, C^6^H); 6.69 (t-br, 1H, CH^Imid^); 6.30 (t-br, 1H, C^β^H); 6.15–6.14 (m, 2H, C^β^H + CH^Imid^); 5.96 (t, 1H, ^3^*J*_HH_ = 2.6 Hz, C^β^H); 3.40 (s, 3H, C^4^H). ^13^C{^1^H} NMR (CDCl_3_): δ/ppm = 148.5, 148.2, 144.1 (C^α^); 143.3 (Imid); 135.6, 135.1, 133.4 (C^γ^); 133.9 (d, ^3^*J*_CP_ = 9.5 Hz, C^7^); 132.9 (d, ^1^*J*_CP_ = 40.1 Hz, C^5^); 130.4 (Imid); 129.7 (C^8^); 128.1 (d, ^2^*J*_CP_ = 9.1 Hz, C^6^); 120.2 (Imid); 108.0, 108.0, 107.9 (C^β^); 74.5 (C^δ^); 34.6 (C^4^). ^31^P{^1^H} NMR (CDCl_3_): δ/ppm = 52.5. Crystals suitable for X-ray diffraction were collected by slow diffusion of diethyl ether into a solution of **3** in methanol/dichloromethane (1:2 v/v) (Chart 3).

#### [RuCl(κ^3^-tpm)(PPh_3_){NCHNHC(CH)_4_C}]Cl, **4** (Chart 4)

**Chart 4:**
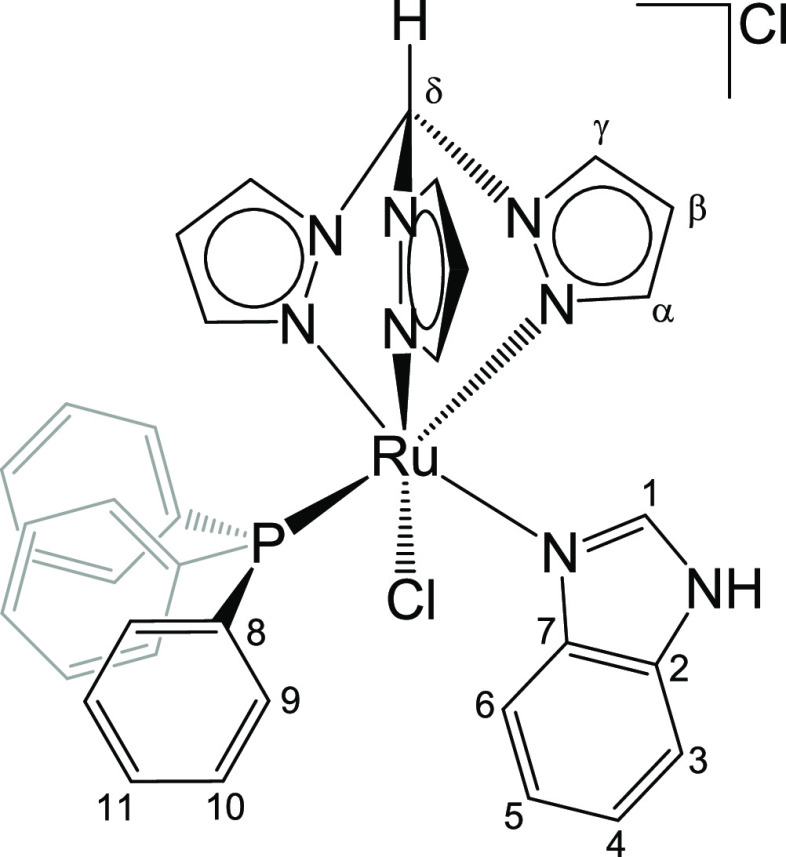
Structure of **4** (Labeling Refers to Carbon Atoms)

From **1** (200 mg, 0.22 mmol) and benzimidazole (40 mg, 0.33 mmol) in ethanol (15 mL). Reaction time: 16 h. Yellow solid, yield 129 mg (77%). Anal. calcd for C_35_H_31_Cl_2_N_8_PRu: C, 54.83; H, 4.08; N, 14.62; Cl, 9.25. Found: C, 54.92; H, 3.98; N, 14.76; Cl, 9.11. IR (solid state): ṽ/cm^–1^ = 3138w, 3104w, 3059w, 2933w-br, 1669w, 1619w, 1587w, 1517w, 1480w, 1449w, 1433m, 1409m, 1304w, 1289m, 1273m, 1245m, 1222m, 1186w, 1152w, 1091m, 1053m, 856m, 794m, 774m, 746s, 740s, 696s. ^1^H NMR (CDCl_3_): δ/ppm = 12.06 (s-broad, 1H, NH); 11.98 (s, 1H, C^δ^H); 8.97, 8.73, 8.66 (d-br, 3H, C^γ^H); 7.76 (s-br, 1H, C^1^H); 7.56 (d, 1H, ^3^*J*_HH_ = 8.1 Hz, C^3^H or C^6^H); 7.34 (t, 3H, ^3^*J*_HH_ = 7.7 Hz, C^11^H); 7.16–6.96 (m, 15H, C^4^H or C^5^H + C^9^H + C^10^H + 2C^α^H); 6.67 (d-br, 1H, C^α^H); 6.60 (t-br, 1H, C^4^H or C^5^H); 6.15, 6.10, 5.92 (t-br, 3H, C^β^H); 5.09 (d-br, 1H, C^3^H or C^6^H). ^13^C{^1^H} NMR (CDCl_3_): δ/ppm = 150.7, 148.7, 145.0 (C^α^); 148.1, 140.6 (C^2^ + C^7^); 135.4, 134.6, 133.2 (C^γ^); 134 (d-br, C^10^); 132.9 (C^1^); 132.3 (d, ^1^*J*_CP_ = 39.2 Hz, C^8^); 130.1 (C^11^); 128.4; (d, ^2^*J*_CP_ = 9.0 Hz, C^9^); 123.5 (C^4^); 122.2 (C^5^); 117.5 (C^6^); 113.1 (C^3^); 108.7, 108.2, 107.8 (C^β^); 74.8 (C^δ^). ^31^P{^1^H} NMR (CDCl_3_): δ/ppm = 51.1. Crystals suitable for X-ray diffraction were collected by slow diffusion of diethyl ether into a dichloromethane solution of **4** (Chart 4).

#### [RuCl(κ^3^-tpm)(PPh_3_){NNH(CH)_3_}]Cl, **5** (Chart 5)

**Chart 5:**
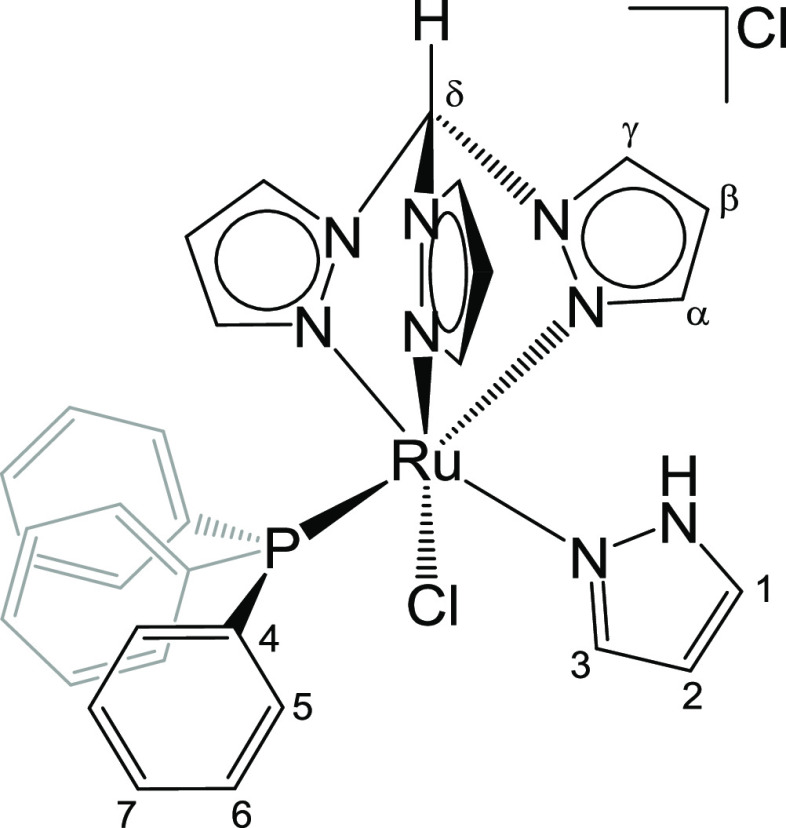
Structure of **5** (Labeling Refers to Carbon Atoms)

From **1** (150 mg, 0.165 mmol) and pyrazole (12 mg, 0.181 mmol) in ethanol (8 mL). Reaction time: 8 h. Yellow solid, yield 106 mg (90%). Anal. calcd For C_31_H_29_Cl_2_N_8_PRu: C, 51.96; H, 4.08; N, 15.64; Cl, 9.90. Found: C, 51.86; H, 3.99; N, 15.55; Cl, 10.01. IR (solid state): ṽ/cm^–1^ = 3452w-br (ṽ_N-H_), 3140w-br, 3109w-br, 2976w, 2924w, 2871w, 1518w, 1483w, 1450w, 1434w, 1407m, 1378w, 1350w, 1290m, 1283m, 1251m, 1223w, 1185w, 1156w, 1126w, 1114w, 1090s, 1050m, 1043m, 999w, 987w, 859m, 790m, 785–775s-br, 696s, 686m. ^1^H NMR (CDCl_3_): δ/ppm = 12.33 (s-br, 1H, NH); 11.79 (s, 1H, C^δ^H); 9.05, 8.81, 8.75 (d, 3H, ^3^*J*_HH_ = 2.8 Hz, C^γ^H); 7.41, 7.09, 6.64 (d-br, 3H, C^α^H); 7.36 (t, 3H, ^3^*J*_HH_ = 7.5 Hz, C^7^H); 7.24 (d-br, 1H, C^3^H); 7.19 (t, 6H, ^3^*J*_HH_ = 7.7 Hz, C^5^H); 6.94 (m, 7H, C^6^H + C^1^H); 6.31 (m, 2H, C^β^H + C^2^H); 6.13, 6.05 (t, 2H, ^3^*J*_HH_ = 2.6 Hz, C^β^H). ^13^C{^1^H} NMR (CDCl_3_): δ/ppm = 148.7, 148.2, 144.0 (C^α^); 141.3 (C^1^); 136.0, 135.4, 133.6 (C^γ^); 133.7 (d, ^3^*J*_CP_ = 9.5 Hz, C^6^); 132.2 (d, ^1^*J*_CP_ = 41.0 Hz, C^4^); 130.3 (C^3^); 130.0 (C^7^); 128.4 (d, ^2^*J*_CP_ = 9.2 Hz, C^5^); 108.5, 107.7 (C^β^); 108.2 (C^β^ + C^2^); 74.5 (C^δ^). ^31^P{^1^H} NMR (CDCl_3_): δ/ppm = 49.9. Crystals suitable for X-ray diffraction were collected by slow diffusion of diethyl ether into a dichloromethane solution of **5** (Chart 5).

#### [RuCl(κ^3^-tpm)(PPh_3_){N(CH)_3_C(OH)CH}]Cl, 6 (Chart 6)

**Chart 6:**
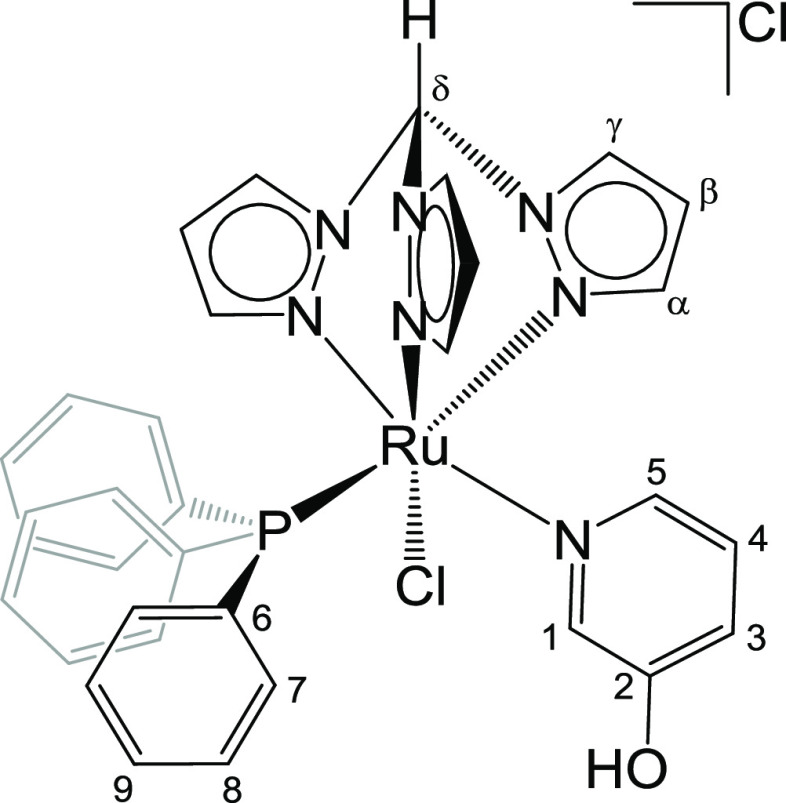
Structure of **6** (labeling refers to carbon atoms)

From **1** (150 mg, 0.165 mmol) and 3-pyridinol (25 mg, 0.26 mmol) in ethanol (10 mL). Reaction time: 16 h. Yellow solid, yield 108 mg (88%). Anal. calcd for C_33_H_30_Cl_2_N_7_OPRu: C, 53.30; H, 4.07; N, 13.19; Cl, 9.54. Found: C, 53.22; H, 4.02; N, 13.20; Cl, 9.65. IR (solid state): ṽ/cm^–1^ = 3668w-br (ṽ_O-H_), 3110w-br, 3052w, 2980w, 2797w, 2773w, 2989w, 2622w, 1589w, 1481w, 1451w, 1436m, 1408m, 1382w, 1288m, 1273m, 1257m, 1222w, 1189w, 1158w, 1087m, 1054w, 1029w, 996w, 988w, 892w, 857m, 792m, 777m, 752s, 699s. ^1^H NMR (CDCl_3_): δ/ppm = 11.53 (s, 1H, C^δ^H); 9.47 (s-br, 1H, OH); 8.72, 8.62, 8.50 (d-br, 3H, C^γ^H); 7.83 (s-br, 1H, C^5^H); 7.60 (s-br, 1H, C^1^H); 7.40 (d, 1H, ^3^*J*_HP_ = 8.4 Hz, C^3^); 7.34 (t, 3H, ^3^*J*_HP_ = 7.4 Hz, C^9^); 7.23 (d-br, 1H, C^α^H); 7.14 (t, 6H, ^3^*J*_HP_ = 7.6 Hz, C^7^); 7.02 (d, 1H, ^3^*J*_HH_ = 2.2 Hz, C^α^H); 6.90 (t, 6H, ^3^*J*_HP_ = 8.8 Hz, C^8^); 6.71 (dd, 1H, ^3^*J*_HH_ = 8.3 Hz, ^3^*J*_HH_ = 5.6 Hz, C^4^); 6.63 (d, 1H, ^3^*J*_HH_ = 2.2 Hz, C^α^H); 6.19 (t-br, 1H, C^β^H); 6.09 (t, 1H, ^3^*J*_HH_ = 2.5 Hz, C^β^H); 6.01 (t, 1H, ^3^*J*_HH_ = 2.4 Hz, C^β^H). ^13^C{^1^H} NMR (CDCl_3_): δ/ppm = 153.8 (C^2^); 148.9, 148.8, 144.5 (C^α^); 135.4 (C^γ^); 134.6, 133.4 (C^γ^); 133.9 (d, ^3^*J*_CP_ = 9.3 Hz, C^8^); 132.0 (d, ^1^*J*_CP_ = 40.7 Hz, C^6^); 130.1 (C^9^); 128.4 (d, ^2^*J*_CP_ = 9.0 Hz, C^7^); 124.0 (C^4^); 123.4 (C^3^); 108.8, 108.6, 108.3 (C^β^); 74.8 (C^δ^). *C*^*1*^*and C*^*5*^*not observed*. ^31^P{^1^H} NMR (CDCl_3_): δ/ppm = 50.6. Λ_m_ (H_2_O, *c* = 1.0 × 10^–3^ M, *t*_0_) = 118 S·cm^2^·mol^–1^; Λ_m_ (H_2_O, *c* = 1.0 × 10^–3^ M, after 24 h) = 240 S·cm^2^·mol^–1^. Crystals suitable for X-ray diffraction were collected by slow diffusion of diethyl ether into a dichloromethane solution of **6** (Chart 6).

#### [RuCl(κ^3^-tpm)(PPh_3_){P(OMe)_3_}]Cl, **7** (Chart 7)

**Chart 7:**
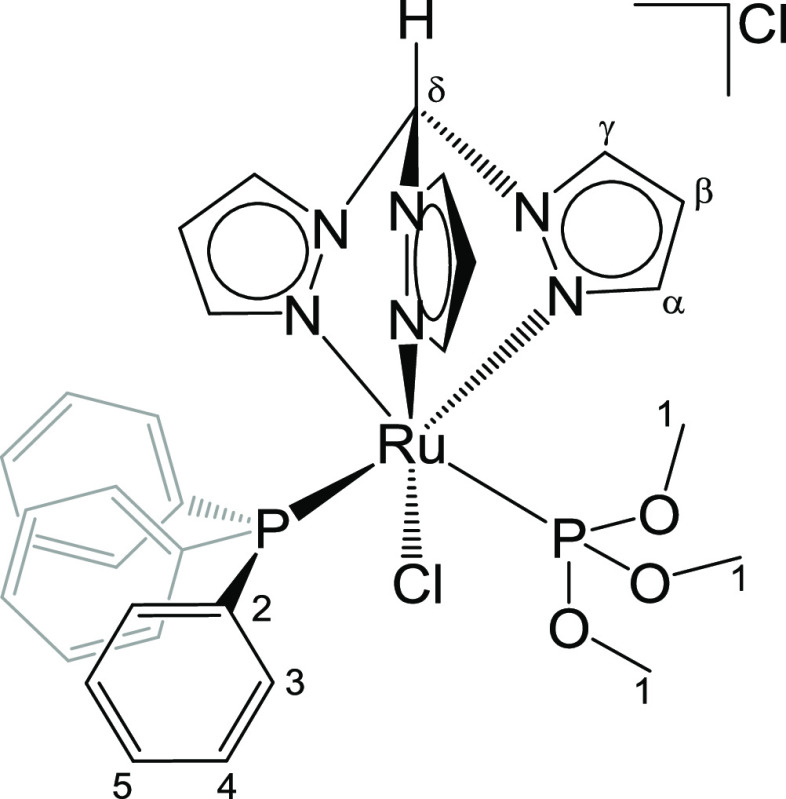
Structure of **7** (Labeling Refers to Carbon Atoms)

From **1** (100 mg, 0.11 mmol) and trimethylphosphite {P(OMe)_3_} (16.9 μL, 0.14 mmol) in anhydrous isopropanol (6 mL). Reaction time: 7 h. Yellow solid, yield 78 mg (94%). Anal. calcd for C_31_H_34_Cl_2_N_6_O_3_P_2_Ru: C, 48.19; H, 4.44; N, 10.88; Cl, 9.18. Found: C, 48.28; H, 4.39; N, 10.67; Cl, 9.27. IR (solid state): ṽ/cm^–1^ = 3118w, 3086w, 3055w, 2952w, 2952–2846w, 1484w, 1461w, 1454w, 1435w, 1405w, 1304w, 1292m, 1257w, 1224w, 1184w, 1093m, 1054–1021s (ṽ_P-O_), 990w, 983w, 856m, 793s, 769s, 762s, 749m, 740m, 728m, 695s, 684m. ^1^H NMR (CDCl_3_): δ/ppm = 12.19 (s, 1H, C^δ^H); 8.92, 8.69 (d, 2H, ^3^*J*_HH_ = 2.8 Hz, C^γ^H); 8.65 (d-br, 1H, C^γ^H); 8.15, 6.83, 6.37 (d, 3H, ^3^*J*_HH_ = 2.2 Hz, C^α^H); 7.4 (t, 6H, ^3^*J*_HH_ = 7.6 Hz, C^3^H); 7.34 (t, 3H, ^3^*J*_HH_ = 7.3 Hz, C^5^H); 7.24 (t, 6H, ^3^*J*_HH_ = 7.5 Hz, C^4^H); 6.34, 5.97 (t-br, 2H, C^β^H); 5.90 (t, 1H, ^3^*J*_HH_ = 2.6 Hz, C^β^H); 3.46 (d, 3H, ^2^*J*_HP_ = 10.2 Hz, C^1^H). ^13^C{^1^H} NMR (CDCl_3_): δ/ppm = 149.2, 146.6, 146.1 (C^α^); 135.8, 133.9, 133.8 (C^γ^); 134.4 (d, *J*_CP_ = 9.21 Hz, C^3^); 133.4 (d, ^1^*J*_CP_ = 43.5 Hz, C^2^); 129.9 (d, ^4^*J*_CP_ = 2.3 Hz, C^5^); 128.0 (d, *J*_CP_ = 9.4 Hz, C^4^); 109.0, 108.0, 107.3 (C^β^); 74.0 (C^δ^); 53.2 (d, ^2^*J*_CP_ = 8.2 Hz, C^1^). ^31^P{^1^H} NMR (CDCl_3_): δ/ppm = 138.0 (d, ^2^*J*_PP_ = 57.8 Hz, P(OMe)_3_); 44.8 (d, ^2^*J*_PP_ = 57.8 Hz, PPh_3_). Crystals suitable for X-ray diffraction were collected by slow diffusion into hexane from an acetone solution of **7** (Chart 7).

#### [RuCl(κ^3^-tpm)(PPh_3_)(CNCy)]Cl, **8** (Chart 8)

**Chart 8:**
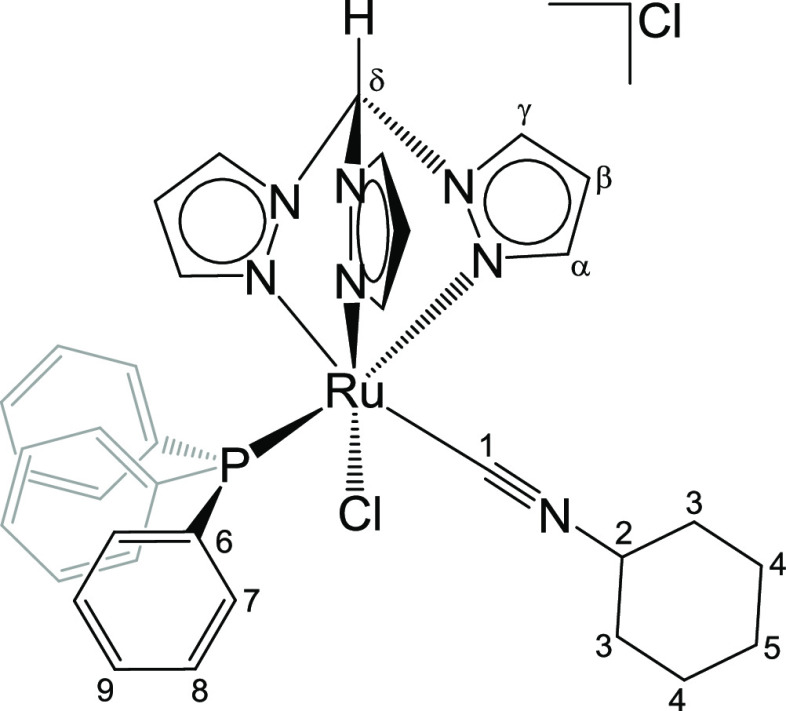
Structure of **8** (Labeling Refers to Carbon Atoms)

From **1** (200 mg, 0.22 mmol) and cyclohexyl isocyanide (30.3 μL, 0.242 mmol) in ethanol (15 mL). Reaction time: 3 h. Yellow solid, yield 157 mg (99%). Anal. calcd for C_35_H_36_Cl_2_N_7_PRu: C, 55.48; H, 4.79; N, 12.94; Cl, 9.36. Found: C, 55.25; H, 4.67; N, 12.88; Cl, 9.26. IR (solid state): ṽ/cm^–1^ = 3103w-br, 3058w, 2937w, 2926w, 2863w, 2854w, 2141s (ṽ_C=N_), 1483w, 1448m, 1437m, 1434m, 1410m, 1403m, 1288s, 1255m, 1250m, 1222w, 1093s, 1084m, 1053m, 856m, 792s, 774s, 758s, 745s, 695s, 688s. ^1^H NMR (CDCl_3_): δ/ppm = 12.28 (s, 1H, C^δ^H); 8.91, 8.72, 8.67 (d, 3H, ^3^*J*_HH_ = 2.8 Hz, C^γ^H); 8.06, 6.22, 6.03 (d, 3H, ^3^*J*_HH_ = 2.2 Hz, C^α^H); 7.49 (t, 6H, ^3^*J*_HH_ = 9.0 Hz, C^7^H); 7.40 (t, 3H, ^3^*J*_HH_ = 7.3 Hz, C^9^H); 7.31 (t, 6H, ^3^*J*_HH_ = 8.6 Hz, ^3^*J*_HH_ = 4.3 Hz, C^8^H); 6.38 (t-br, 1H, C^β^H); 6.07, 5.91 (t, 2H, ^3^*J*_HH_ = 2.5 Hz, C^β^H); 3.90 (m, 1H, C^2^-H); 1.90 (m, 2H, C^4^H); 1.66 (m, 2H, C^3^H); 1.59 (m, 2H, C^4^H); 1.49 (m, 1H, C^5^H); 1.30 (m, 3H, C^3^H + C^5^H). ^13^C{^1^H} NMR (CDCl_3_): δ/ppm = 155.9 (C^1^); 147.3, 145.9, 144.7 (C^α^); 135.9, 134.3, 133.4 (C^γ^); 134.2 (C^7^, ^3^*J*_PC_ = 9.6 Hz); 132.8 (C^6^, ^1^*J*_PC_ = 44.8 Hz); 130.2 (C^6^, ^4^*J*_PC_ = 2.21 Hz); 128.4 (C^8^, ^3^*J*_PC_ = 9.6 Hz); 108.2, 108.0, 107.8 (C^β^); 74.2 (C^δ^); 55.4 (C^2^); 33.7 (C^4^); 24.9 (C^5^); 23.3 (C^3^). ^31^P{^1^H} NMR (CDCl_3_): δ/ppm = 49.6. Crystals suitable for X-ray diffraction were collected by slow diffusion of diethyl ether into a CH_2_Cl_2_ solution of **8** (Chart 8).

#### [RuCl(κ^3^-tpm)(PPh_3_){CNCH_2_P(O)(OEt_2_)_2_}]Cl, **9** (Chart 9)

**Chart 9:**
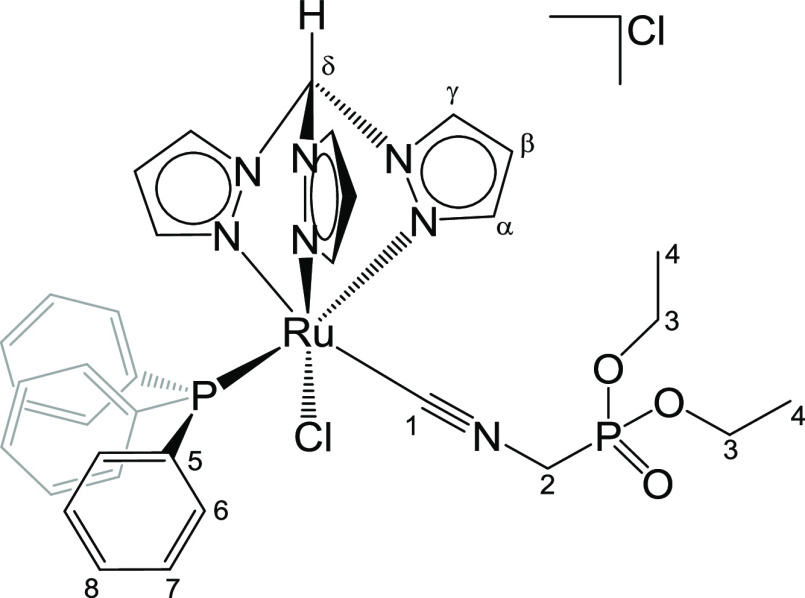
Structure of **9** (Labeling Refers to Carbon Atoms)

From **1** (200 mg, 0.22 mmol) and diethyl isocyanomethyl phosphonate (38.8 μL, 0.242 mmol) in ethanol (15 mL). Reaction time: 3 h. Yellow solid, yield 159 mg (92%). Anal. calcd for C_34_H_37_Cl_2_N_7_O_3_P_2_Ru: C, 49.46; H, 4.52; N, 11.88; Cl, 8.59. Found: C, 49.33; H, 4.57; N, 11.96; Cl, 8.68. HR-ESI-MS: [M]^+^*m*/*z* = 790.1171 (theoretical for [C_34_H_37_ClN_7_O_3_P_2_Ru]^+^: *m*/*z* = 790.1165). IR (solid state): ṽ/cm^–1^ = 3149w, 3123w, 3109w, 3061w, 3001w, 2981w, 2933w, 2905w, 2147s (ṽ_C=N_), 1515w, 1483w, 1479w, 1455w, 1445w, 1437m, 1433m, 1410m, 1397w, 1339w, 1293m, 1258m, 1245s (ṽ_P=O_), 1227m, 1191w, 1164w, 1159w, 1090s, 1055m, 1042s, 1011s, 990m, 982m, 971s, 938m, 918w, 8895w, 857m, 836w, 815m, 793m, 768m, 763s, 750s, 745s, 699s, 695s, 682m. ^1^H NMR (CDCl_3_): δ/ppm = 12.24 (s, 1H, C^δ^H); 8.86, 8.77 (d, 2H, ^3^*J*_HH_ = 2.8 Hz, C^γ^H); 8.64 (d-br, 1H, C^γ^H); 8.12, 6.85, 6.44 (d, 3H, ^3^*J*_HH_ = 2.2 Hz, C^α^H); 7.47–7.39 (m, 9H, C^6^H + C^8^H); 7.31 (m, 6H, C^7^H); 6.38 (t-br, 1H, C^β^H); 6.13, 5.90 (t, 2H, ^3^*J*_HH_ = 2.5 Hz, C^β^H); 4.11–3.86 (m, 6H, C^2^H + C^3^H); 1.20 (t, 3H, ^3^*J*_HH_ = 7.2 Hz, C^4^H); 0.99 (t, 3H, ^3^*J*_HH_ = 7.1 Hz, C^4^H). ^13^C{^1^H} NMR (CDCl_3_): δ/ppm = 163.5 (C^1^); 148.3, 146.0, 145.1 (C^α^); 135.7, 134.6, 133.4 (C^γ^); 134.1 (d, ^2^*J*_CP_ = 9.7 Hz, C^6^); 132.2 (d, ^1^*J*_CP_ = 45.6 Hz, C^5^); 130.4 (C^8^); 128.5 (d, ^3^*J*_CP_ = 9.7 Hz, C^7^); 108.1, 108.1, 107.9 (C^β^); 74.2 (C^δ^); 64.0 (d, ^2^*J*_CP_ = 6.8 Hz, C^3^); 63.7 (d, ^2^*J*_CP_ = 6.6 Hz, C^3^); 41.1 (d, ^1^*J*_CP_ = 156.3 Hz, C^2^); 16.4, 16.1 (d, ^3^*J*_CP_ = 5.6 Hz, C^4^). ^31^P{^1^H} NMR (CDCl_3_): δ/ppm = 47.2 (s, PPh_3_); 15.7 (s, PCH_2_) (Chart 9).

### X-ray Crystallography

Crystal data and collection details for **3·2CH**_**3**_**OH**, **4·0.5Et**_**2**_**O·solv**, **5·CH**_**2**_**Cl**_**2**_**·2H**_**2**_**O**, **6·solv**, **7·CH**_**3**_**COCH**_**3**_**·H**_**2**_**O**, and **8·2H**_**2**_**O** are reported in Table 6. Data were recorded on a Bruker APEX II diffractometer equipped with a PHOTON2 detector using Mo Kα radiation. The structures were solved by direct methods and refined by full-matrix least-squares based on all data using *F*^2^.^111^ Hydrogen atoms were fixed at calculated positions and refined using a riding model.^112^ The crystals of **4·0.5Et**_**2**_**O·solv** and **6·solv** contain some highly disordered solvent molecules that have been treated using the SQUEEZE routine of PLATON.^113,114^ The refined crystal structures of **4·0.5Et**_**2**_**O·solv** and **5·CH**_**2**_**Cl**_**2**_**·2H**_**2**_**O** contain large difference peaks; these are located in the proximity of disordered chloride ions or CH_2_Cl_2_ molecules.

**Table 6:** Crystal Data and Measurement Details for **3·2CH_3_OH**, **4·0.5Et_2_O·solv**, **5·CH_2_Cl_2_·2H_2_O**, **6·solv**, **7·CH_3_COCH_3_·H_2_O**, and **8·2H_2_O**

	**3·2CH_3_OH**	**4·0.5Et_2_O·solv**	**5·CH_2_Cl_2_·2H_2_O**	**6·solv**	**7·CH_3_COCH_3_·H_2_O**	**8·2H_2_O**
formula	C_34_H_39_Cl_2_N_8_O_2_PRu	C_37_H_36_Cl_2_N_8_O_0.5_PRu	C_32_H_35_Cl_4_N_8_O_2_PRu	C_33_H_30_Cl_2_N_7_OPRu	C_34_H_42_Cl_2_N_6_O_5_P_2_Ru	C_35_H_40_Cl_2_N_7_O_2_PRu
FW	794.67	803.68	837.52	743.58	848.64	793.68
*T*, K	100(2)	100(2)	100(2)	100(2)	100(2)	100(2)
λ, Å	0.71073	0.71073	0.71073	0.71073	0.71073	0.71073
crystal system	triclinic	monoclinic	triclinic	triclinic	monoclinic	monoclinic
space group	*P*1̅	**P**2_1_/*c*	*P*1̅	*P*1̅1̅	**C**2/*c*	**P**2_1_/*c*
*a*, Å	10.1428(8)	20.5145(12)	9.7016(8)	11.1525(4)	41.370(8)	17.110(2)
*b*, Å	13.2743(11)	20.6593(12)	10.0588(8)	12.4862(5)	9.756(2)	10.1025(13)
*c*, Å	13.9235(11)	19.0486(11)	19.2103(16)	14.7685(6)	19.152(4)	21.524(3)
α, deg	72.776(2)	90	97.728(2)	87.2220(10)	90	90
β, deg	77.299(2)	100.126(2)	103.181(3)	73.5550(10)	91.34(3)	106.679(8)
γ, deg	85.255(3)	90	97.790(2)	89.3910(10)	90	90
cell volume, Å^3^	1746.4(2)	7947.3(8)	1781.7(3)	1970.09(13)	7728(3)	3564.1(8)
*Z*	2	8	2	2	8	4
*D*_c_, g·cm^–3^	1.511	1.343	1.561	1.253	1.459	1.479
μ, mm^–1^	0.692	0.607	0.828	0.606	0.674	0.677
*F*(000)	816	3288	852	756	2488	1632
crystal size, mm	0.16 × 0.13 × 0.10	0.22 × 0.16 × 0.14	0.22 × 0.18 × 0.15	0.18 × 0.15 × 0.12	0.24 × 0.21 × 0.18	0.18 × 0.13 × 0.10
θ limits, deg	1.5649–26.998	1.467–25.998	2.074–25.997	1.633–27.227	1.970–26.000	1.9759–26.998
reflections collected	32 233	142 792	29 925	27 297	54 659	72 769
independent reflections	75 857 [*R*_int_ = 0.0850]	15 547 [*R*_int_ = 0.1422]	6987 [*R*_int_ = 0.0724]	8722 [*R*_int_ = 0.0465]	7594 [*R*_int_ = 0.0553]	7780 [*R*_int_ = 0.0673]
data/restraints/parameters	7585/17/428	15 547/8/871	6987/39/445	8722/0/407	7594/130/473	7780/6/445
goodness on fit on *F*^2 ^a	1.193	1.075	1.036	1.097	1.038	1.064
*R*_1_ (*I* > 2σ(*I*))^b^	0.0628	0.0799	0.0610	0.0486	0.0336	0.0308
*w*R**_2_ (all data)^c^	0.1366	0.1881	0.1604	0.1053	0.0772	0.0767
largest diff. peak and hole, e Å^–3^	1.972/–1.232	3.653/–1.407	3.443/–2.006	1.132/–0.668	1.028/–0.793	0.651/–0.568

a Goodness on fit on *F*^2^ = [Σ*w*(*F*_O_^2^ – *F*_C_^2^)^2^/(*N*_ref_ – *N*_param_)]^1/2^, where *w* = 1/[σ^2^(*F*_O_^2^) + (*aP*)^2^ + *bP*], where *P* = (*F*_O_^2^ + 2*F*_C_^2^)/3; *N*_ref_ = number of reflections used in the refinement; *N*_param_ = number of refined parameters.

b *R*_1_ = Σ||*F*_O_| – |*F*_C_||/Σ|*F*_O_|.

c w*R*_2_ = [Σ*w*(*F*_O_^2^ – *F*_C_^2^)^2^/Σ*w*(*F*_O_^2^)^2^]^1/2^, where *w* = 1/[σ^2^(*F*_O_^2^) + (*aP*)^2^ + *bP*], where *P* = (*F*_O_^2^ + 2*F*_C_^2^)/3.

### Behavior in Aqueous Media

#### (a) Solubility in Water

A suspension of the selected ruthenium complex (3–5 mg) in a D_2_O solution (0.7 mL) containing Me_2_SO_2_ as the internal standard^115^ (3.36 × 10^–3^ M) was vigorously stirred at 21 °C for 2 h. The resulting saturated solution was filtered over celite, transferred into an NMR tube, and analyzed by ^1^H NMR and ^31^P NMR spectroscopy (delay time = 3 s; number of scans = 20). The concentration (solubility) was calculated by the relative integral (starting complex + aquo complex, C^β^H signal in the ^1^H spectrum) with respect to Me_2_SO_2_ (δ/ppm = 3.14). The results are compiled in Table 2. NMR data are given in the Supporting Information (Figures S36–S47).

#### (b) Stability in D_2_O and DMSO-*d*_6_/D_2_O

The same samples prepared at point (a) were used in this experiment, apart from complex **1** that was dissolved in DMSO-*d*_6_/D_2_O 4:1 v/v solution (0.7 mL; [Ru] = approx. 3 mg) containing Me_2_SO_2_ as the standard.^115^ After ^1^H and ^31^P{^1^H} NMR analyses described above at point (a) (time = *t*_0_), samples were heated at 37 °C for 48 h. After cooling to room temperature, ^1^H and ^31^P NMR spectra were recorded. The percentage of starting complex + related aquo complex was calculated by the relative integral (C^β^H signal in the ^1^H spectrum) with respect to Me_2_SO_2_ (*c* = 3.3 × 10^–3^ mol·L^–1^; δ/ppm = 3.14 in D_2_O; δ/ppm = 2.95 in DMSO-*d*_6_/D_2_O 4:1 v/v), see Table 2 and S1.

#### (c) Octanol/Water Partition Coefficients (log *P*_ow_)

Partition coefficients (*P*_ow_); IUPAC: *K*_D_ partition constant,^116^ defined as *P*_ow_ = *c*_org_/*c*_aq_, where *c*_org_ and *c*_aq_ are the molar concentrations of the selected compound in the organic and aqueous phase, respectively, were determined by the shake-flask method and UV–vis measurements.^62,117,118^ Deionized water and 1-octanol were vigorously stirred for 24 h to enable saturation of both phases and then separated by centrifugation. A stock solution of the selected ruthenium compound (ca. 2 mg) was prepared by first adding MeOH (50 μL, to help solubilization), followed by octanol-saturated water (2.5 mL). The solution was diluted with octanol-saturated water (ca. 1:3 v/v ratio, *c*_Ru_ ≈ 10^–4^ M, so that 1.5 ≤ *A* ≤ 2.0 at λ_max_), and its UV–vis spectrum was recorded (*A*_aq_^0^). An aliquot of the solution (*V*_aq_ = 1.2 mL) was transferred into a test tube, and water-saturated octanol (*V*_org_ = *V*_aq_ = 1.2 mL) was added. The mixture was vigorously stirred for 20 min at 21 °C then centrifuged (5000 rpm, 5 min). The UV–vis spectrum of the aqueous phase was recorded (*A*_aq_^f^), and the partition coefficient was calculated as *P*_ow_ = (*A*_aq_^0^ – *A*_aq_^f^)/*A*_aq_^f^, where *A*_aq_^0^ and *A*_aq_^f^ are the absorbances in the aqueous phase before and after partition with the organic phase, respectively.^118^ For **1**, an inverse procedure was followed, starting from a solution of the compound in water-saturated octanol. The partition coefficient was calculated as *P*_ow_ = *A*_org_^f^/(*A*_org_^0^ – *A*_org_^f^), where *A*_org_^0^ and *A*_org_^f^ are the absorbances in the organic phase before and after partition with the aqueous phase, respectively. The wavelength of the maximum absorption of each compound (280–380 nm range) was used for UV–vis quantitation. The procedure was repeated three times for each sample (from the same stock solution); the results are given as mean ± standard deviation (Table 2). Naphthoquinone was used as a reference compound (log *P* = 1.8 ± 0.2; literature:^119^ 1.71).

#### (d) Stability in Cell Culture Medium

Powdered DMEM cell culture medium (1000 mg/L glucose and l-glutamine, without sodium bicarbonate and phenol red; D2902; Sigma-Aldrich) was dissolved in D_2_O (10 mg/mL), according to the manufacturer’s instructions. The solution of deuterated cell culture medium (“DMEM-*d*”) was treated with Me_2_SO_2_ (6.6 × 10^–3^ M) and NaH_2_PO_4_/Na_2_HPO_4_ (0.10 M, p*D* = 7.5)^120−122^ and then stored at 4 °C under N_2_. The selected ruthenium compound (2–3 mg) was dissolved in DMSO-*d*_6_ (0.14 mL; 0.18 mL for **4**) and then diluted with DMEM-*d* up to 0.75 mL total volume (*c*_Ru_ ca. 4 × 10^–3^ M). The mixture was stirred for 30 min, then filtered over celite, and transferred into an NMR tube. The resulting yellow solution was analyzed by ^1^H and ^31^P NMR (delay time = 3 s; number of scans = 20) and then heated at 37 °C for 24 h. After cooling to room temperature, NMR analyses were repeated. Compound **1** was instead dissolved in a DMSO-*d*_6_/DMEM-*d* 4:1 v/v solution (0.7 mL; [Ru] = approx. 3 × 10^–3^ M) containing Me_2_SO_2_ as the internal standard.^115^ The percentage of the starting complex + related aquo complex was calculated by the relative integral with respect to Me_2_SO_2_ (δ/ppm = 3.16 in DMSO-*d*_6_/DMEM-*d* 1:4 and 1:3 v/v; δ/ppm = 2.95 in DMSO-*d*_6_/DMEM-*d* 4:1 v/v).

### Biological Studies

#### Cell Lines, Culture Conditions, and Stock Solutions of Ru Complexes

The human cervical carcinoma HeLa cells and human colorectal carcinoma cells HCT116 were kindly supplied by Professor B. Keppler, University of Vienna (Austria). Human rhabdomyosarcoma RD cells were purchased from the American Type Culture Collection (ATCC, Manassas, VA). Human breast cancer MCF-7 cells, human skin melanoma 518A2 cells, and human MRC5pd30 cells derived from normal lung tissue were purchased from the European Collection of Authenticated Cell Cultures (ECACC) (Salisbury, U.K.). Chinese hamster ovary CHO-K1 cell line (wild type) and its derivative MMC-2 carrying the ERCC3/XPB mutation (NER-deficient) cell line were kindly supplied by Dr. M. Pirsel, Cancer Research Institute, Slovak Academy of Sciences, Bratislava (Slovakia).

All of the cell lines were cultivated in DMEM medium (high glucose 4.5 g L^–1^, PAA, Pasching, Austria) supplemented with gentamycin (50 μg mL^–1^, Serva, Heidelberg, Germany) and 10% heat-inactivated fetal bovine serum (PAA). The medium for MRC5pd30 cells was further supplemented by 1% nonessential amino acids (Sigma-Aldrich, Prague, Czech Republic). All cells were cultured as adherent monolayers in a humidified incubator at 37 °C in a 5% CO_2_ atmosphere and subcultured twice a week with an appropriate plating density.

For biological studies, stock solutions of Ru complexes were prepared by dissolving the compounds in DMSO to a final concentration of 10 mM and subsequently diluted to the media to the required concentration. The concentration of Ru in media used in the experiment was verified by flameless atomic absorption spectrometry (FAAS). The final DMSO concentration in the cell culture medium did not exceed 1% (v/v) to avoid DMSO toxicity.

#### Antiproliferative Activity

*In vitro* antiproliferative activity of Ru complexes was determined by the MTT or, alternatively, SRB assay as already described,^84^ after 72 h of incubation of cells with various concentrations of the Ru complex. The reported IC_50_ values are an average of three independent experiments, each consisting of three replicates per concentration.

#### Cellular Uptake

In these experiments, 1 × 10^6^ HCT116 cells were seeded on 100 mm Petri dishes. After overnight preincubation in a drug-free medium, the cells were treated with the Ru complexes (15 μM) for 24 h. Afterward, the cells were extensively washed with PBS (37 °C), detached using 0.25% trypsin, washed twice with ice-cold PBS, and counted by an automatic cell counter. The cell pellets were digested using a microwave acid (HCl) digestion system (CEM Mars). The quantity of Ru taken up by the cells was determined by inductively coupled plasma mass spectrometry (ICP-MS). All experiments were carried out in triplicate.

#### Annexin-V/PI Staining

The type of cell death (apoptosis/necrosis) caused by studied Ru complexes was determined by flow cytometry using Annexin-V and propidium iodide staining after 24 h of treatment. HCT116 cells were seeded in a six-well plate at a density of 150000 cells/well. After overnight cultivation, the cells were treated with studied complexes and incubated for 24 h. Afterward, the cells from individual wells were collected. Pellets were resuspended in the Annexin-V/PI staining solution (BD Pharmingen), and the samples were analyzed using a BD FACSverse flow cytometer.

#### Real-Time Apoptosis/Necrosis

Type of cell death and its kinetics were measured using the Real Time-Glo Annexin-V Apoptosis and Necrosis Assay (Promega). HCT116 cells were seeded at a density of 8 × 10^3^ cells/50 μL in a 96-well black plate and incubated overnight. The cells were then treated with the Ru complexes, and immediately afterward, kinetic analysis began. Staurosporine (10 μM) and ethanol (5%) were used as positive controls of apoptosis and necrosis, respectively. Luminescence (integration of 1000 ms) and fluorescence (λ_ex_: 485 nm; λ_em_: 535 nm) were detected by a SPARK reader (Tecan, Manedorf, Switzerland).

#### Real-Time Cell Growth Monitoring

We employed an xCELLigence RTCA SP Instrument (ROCHE) for monitoring cell growth in real time. First, the background of the 96-well E-Plate was read (100 μL of cultivation media). Then, the cells were added to the E-Plate (2000 cells/well in 50 μL of media), and the measurement was started. After 21 h, Ru complexes were added at various concentrations, and impedance was monitored for 72 h. An arbitrary unit CI (cell index) is a quantitative measure in which the status of the cells (number and morphology of attached cells) is reflected.

#### Cell Fractionation

The HCT116 cells were seeded at a density of 1.5 × 10^6^ cells/10 mL Petri dish and incubated overnight. Then, the cells were treated with 10 μM Ru complexes, incubated for 5 or 24 h, harvested, and exhaustively washed with PBS. The cell pellets were processed by the FractionPREP Cell Fractionation Kit (BioVision) according to the manufacturer’s instructions, yielding four subcellular fractions: cytosol, membrane/particulate, nuclear, and cytoskeletal. The Ru content in each fraction was evaluated by ICP-MS. The measurement was performed in triplicate.

#### Isolation of Mitochondria

The HCT116 cells were seeded at a density of 3 × 10^6^ cells/10 mL Petri dish and incubated overnight. The cells were treated with Ru complexes (10 μM) for 24 h, harvested, and washed with ice-cold PBS, and mitochondrial fractions were extracted by the Mitochondrial Isolation Kit (MITOISO2, Sigma-Aldrich) according to the manufacturer’s instructions. The Ru content in each sample was evaluated by ICP-MS. The measurement was performed in triplicate.

#### Effect on Mitochondrial Membrane Potential

Effects of Ru complexes on mitochondrial membrane potential were assessed by TMRE staining of HCT116 cells after treatment. First, HCT116 cells were seeded on a six-well plate at a 1.5 × 10^5^ cells/well density. The next day, the cells were treated with equimolar (10 μM) and equitoxic (twofold or fourfold IC_50,72h_) concentrations of Ru complexes for 5 h. Then, the cells were collected and stained with 100 nM TMRE in a complete DMEM medium for 30 min at 37 °C in the dark. The TMRE-containing medium was then replaced with PBS, and cells were analyzed by a BD FACSverse flow cytometer (λ_ex_ = 488 nm, λ_em_ = 586 nm).

#### Effect on Oxidative Phosphorylation

The Mitochondrial Tox-Glo assay was used to determine whether studied Ru complexes influence oxidative phosphorylation in HCT116 cells. Two culture media were prepared: glucose-containing, serum-free RPMI medium (RPMI 1640, + l-glutamine, 10 mM D-glucose (Gibco, Thermo Fisher Scientific)) supplemented with sodium bicarbonate and glucose-free, serum-free RPMI medium (RPMI 1640, + l-glutamine, no glucose (Gibco, Thermo Fisher Scientific)) supplemented with 10 mM galactose (Sigma) and sodium bicarbonate. Cells were seeded at a density of 1.5 × 10^3^ cells/well in 50 μL in a 96-well black plate in media containing either glucose or galactose (vide supra) and incubated overnight. Then, the cells were treated with 50 μL of Ru complexes in various concentrations as indicated. Cells were cultivated for 90 min at 37 °C. Cell membrane permeability and ATP quantity were determined by the Mitochondrial Tox-Glo assay according to the manufacturer’s protocol. The first step was to add a fluorogenic peptide substrate (bis-AAF-R110) to measure the dead cell protease activity. Bis-AAF-R110 substrate cannot cross the intact membrane of live cells and therefore gives an insignificant signal with viable cells relative to nonviable cells. The second step of the procedure is adding an ATP detection reagent, resulting in cell lysis and generating a luminescent signal proportional to the amount of ATP present. The final fluorescent and luminescent signals were detected on multimode reader SPARK (Tecan, Manedorf, Switzerland).

#### Calcium Flux

The distribution of calcium ions in cytosol and mitochondria was studied using calcium-sensitive fluorescent probes. HCT116 cells were seeded at a density of 2 × 10^5^ cells/well on six-well plates. After overnight cultivation, the cells were treated with Ru complexes at indicated concentrations for 2.5 h. The culture medium was then replaced with either 5 μM Rhod-2 or Fluo-4 in PBS supplemented with 2 mM CaCl_2_ and incubated for 30 min at 37 °C. At last, cells were harvested and suspended in PBS (with 2 mM CaCl_2_) or in PBS (with 2 mM CaCl_2_) supplemented with ionomycin (5 μM) for 30 min at 37 °C and analyzed using a BD FACSverse flow cytometer.

#### Cytotoxicity in Colonospheres

A spontaneous spheroid formation was used to generate colonospheres derived from the HCT116 cell line. The HCT116 cells were seeded on 96-well ultralow attachment plates (1400 cells/well) and cultivated in a tumor sphere-forming medium (DMEM/F12, supplemented with B27 (Invitrogen), BSA, bFGF (10 ng mL^–1^), and EGF (20 ng mL^–1^)) for 4 days. After the incubation period, colonospheres were treated with Ru complexes for another 3 days. The viability of colonospheres was determined by the Cell Titer-Glo 3D cell viability assay (Promega) according to the manufacturer’s instructions. The reported IC_50_ values are the average of three independent experiments, each consisting of three replicates per concentration level. Bright-field images of the spheroids were taken as well to determine the effect on the morphology of the spheroids.

#### Confocal Microscopy Imaging of Actin and Tubulin

HCT116 cells were seeded on coverslips precoated with 0.1% gelatin in six-well culture plates at a density of 1 × 10^5^ cells/well. The following day, the cells were treated with the tested compounds at concentrations corresponding to IC_50_ for 24 h. Following the treatment, the cells were washed with PBS, fixed with 4% p-formaldehyde, washed, permeabilized with 0.1% Triton X-100, and blocked. Samples for actin staining were blocked with 1.5% BSA for 1 h and then stained with Alexa Fluor 488-conjugated Phalloidin (Thermo Fisher Scientific, 1:50 dilution, 20 min). Samples for tubulin staining were blocked with 5% goat serum for 1 h and incubated with primary antibody (anti-α-tubulin, Abcam, 1:200 dilution, 1 h) and Alexa Fluor-conjugated secondary antibody (goat antirabbit, Abcam, 1:500 dilution, 1 h). Both groups of samples were mounted with ProLong Diamond Antifade Mountant with DAPI (Invitrogen). Cells were visualized on a confocal microscope Leica TCS SP8 SMD.

## Supporting Information Available

Solid-state IR and NMR spectra in CDCl_3_ of ruthenium complexes, NMR data and spectra of complexes in aqueous solutions, biological data, representative HR-ESI-MS spectra (PDF)
Data_3, loop, shelx_space_group_comment (CIF)
Molecular formula strings (CSV)

## Supplementary Material

jm2c00722_si_001.pdf

jm2c00722_si_002.cif

## Accession Codes

CCDC reference numbers 2167597 (**3**), 2167598 (**4**), 2167599 (**5**), 2167600 (**6**), 2167601 (**7**) and 2167602 (**8**) contain the supplementary crystallographic data for the X-ray studies reported in this work. Authors will release the atomic coordinates upon article publication.

## Abbreviations Used

δ: chemical shift in parts per million downfield from tetramethylsilane
μ: micro
Å: angstrom(s)
°C: degrees celsius
3D: three-dimensional
518A2: human skin melanoma cells
aq: aqueous
ATP: adenosine triphosphate
BSA: bovine serum albumin
CHO-K1: Chinese Hamster ovary cell line
CI: cell index
Cy: cyclohexyl
DMSO: dimethyl sulfoxide
DMEM: Dulbecco’s modified Eagle’s medium
Et: ethyl
FAAS: flameless atomic absorption spectrometry
FACS: fluorescence-activated cell sorting
FIA: flow injection analysis
HCT116: human colon tumor cell line 116
HeLa: cervical cancer cell line
HR-ESI-MS: high-resolution electron spray ionization mass spectroscopy
IC_50_: concentration that causes 50% inhibition of cell proliferation
ICP-MS: inductively coupled plasma mass spectrometry
KP1019: indazolium trans-[tetrachlorobis(1H-indazole)ruthenate(III)]
log *P*_ow_: octanol–water partition coefficient
MCF-7: human breast cancer cells (Michigan Cancer Foundation-7)
Me: methyl
mTOR: mammalian target of rapamycin
MTT: (3-(4,5-dimethylthiazol-2-yl)2,5-diphenyl-tetrazolium bromide)
NAMI-A: imidazolium trans-[tetrachloro(dimethylsulfoxide)imidazoleruthenate(III)]
NER: nucleotide excision repair
NMR: nuclear magnetic resonance
ORTEP: Oak Ridge thermal ellipsoid plot
OXPHOS: oxidative phosphorylation
PBS: phosphate-buffered saline
Ph: phenyl
PI: propidium iodide
ppm: part(s) per million
PTA: 1,3,5-triaza-7-phosphaadamantane
Q-TOF: quadrupole-time-of-flight
RAPTA: ruthenium arene 1,3,5-triaza-7-phosphaadamantane complexes
RD: rhabdomyosarcoma
RT: room temperature
RTCA: real-time cell analyzer
SI: selectivity index
SRB: sulphorhodamine B
SEM: standard error of the mean
SRB: sulphorhodamine B
TCRPs: time-dependent cell response profiles
TMRE: tetramethylrhodamine ethyl ester
tpm: tris(pyrazolyl)methane
