# Supplementary material for: Ruthenium(II)–Tris-pyrazolylmethane Complexes Inhibit Cancer Cell Growth by Disrupting Mitochondrial Calcium Homeostasis
Source: J Med Chem. 2022 Aug 1;65(15):10567–87. doi: 10.1021/acs.jmedchem.2c00722 (PMC9376960; doi:10.1021/acs.jmedchem.2c00722)

Supporting Information

Ruthenium(II) Tris-Pyrazolylmethane Complexes Inhibit Cancer Cell Growth by Disrupting Mitochondrial Calcium Homeostasis

Jakub Cervinka,[a,b,#] Alberto Gobbo,[c,d,#] Lorenzo Biancalana,[c] Lenka Markova,[a] Vojtech Novohradsky,[a] Massimo Guelfi,[c] Stefano Zacchini,[d] Jana Kasparkova,[a,e] Viktor Brabec,[a,*] Fabio Marchetti[c,*]

[a] Czech Academy of Sciences, Institute of Biophysics, Kralovopolska 135, CZ-61265 Brno, Czech Republic.

[b] Masaryk University, Faculty of Science, Department of Biochemistry, Kamenice 5, CZ-62500 Brno, Czech Republic

[c] University of Pisa, Department of Chemistry and Industrial Chemistry, Via G. Moruzzi 13, I-56124 Pisa, Italy.

[d] University of Bologna, Department of Industrial Chemistry "Toso Montanari", Viale Risorgimento 4, I-40136 Bologna, Italy.

[e] Palacky University in Olomouc, Faculty of Science, Department of Biophysics, Slechtitelu 27, CZ-78371 Olomouc, Czech Republic

Corresponding Authors: brabec@ibp.cz, fabio.marchetti1974@unipi.it. [#]J. C. and A. G. contributed equally.

**Table of contents**	**Pages**
IR spectra of ruthenium complexes (**Figures S1–S9**)	S2-S4
NMR spectra of ruthenium complexes (**Figures S10–S35**)	S5-S21
NMR analyses in D_2O (**Figures S36–S47**)	S22-S31
Experiments on chloride/water exchange (**Figures S48-S49**)	S32-S34
^{31}P NMR spectra of **2** in DMSO-d_6/DMEM-d 1:4 v/v (**Figure S50**)	S35
Overall data on speciation, IC_{50} and Log P_{ow} values of the complexes (**Table S1**)	S36
Biological data (**Figures S51-S57** and **Tables S2-S3**)	S37-S40
HR-ESI-MS spectrum of **9** (**Figure S58**)	S41

IR spectra

Figure S1. Solid-state IR spectrum (650-4000 cm^{-1}) of **1.**

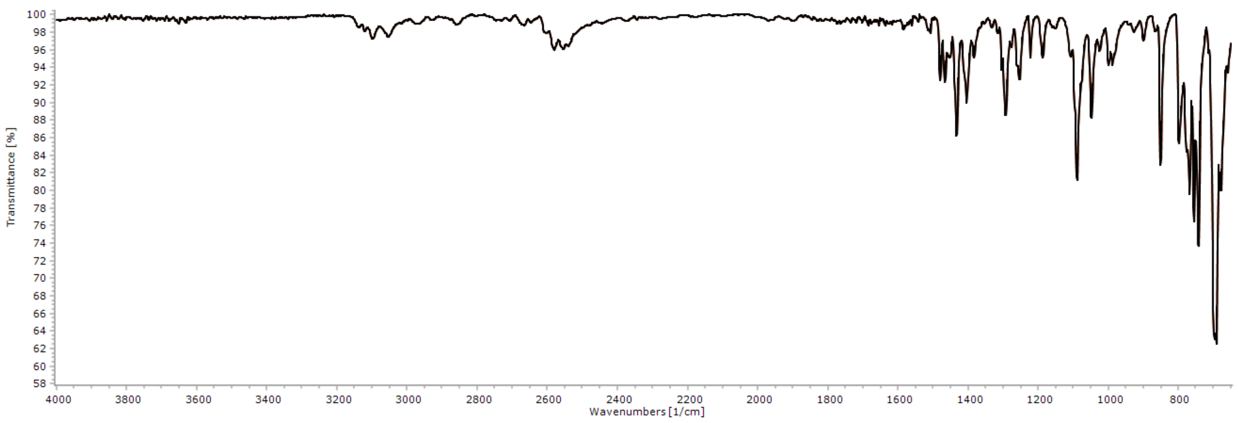

Figure S2. Solid-state IR spectrum (650-4000 cm^{-1}) of **2.**

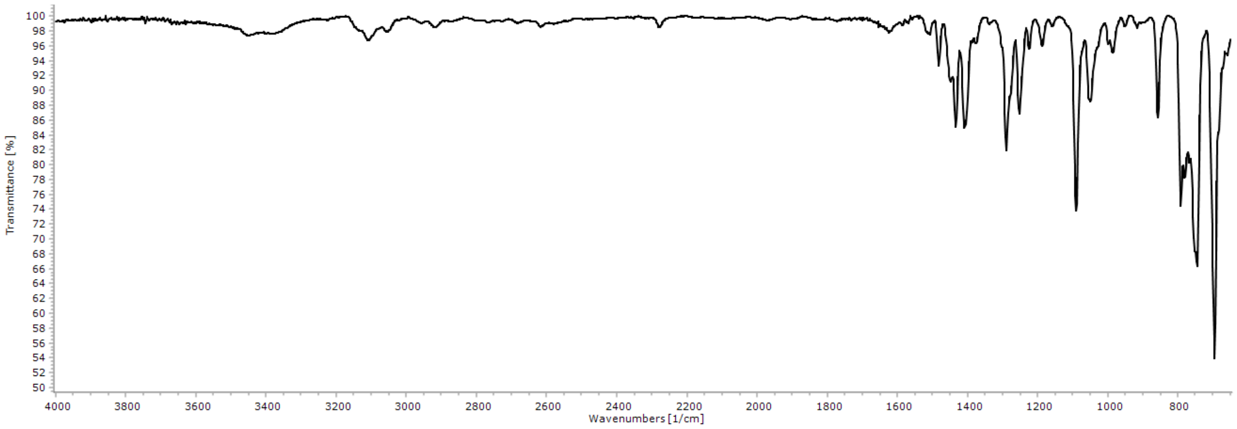

Figure S3. Solid-state IR spectrum (650-4000 cm^{-1}) of **3.**

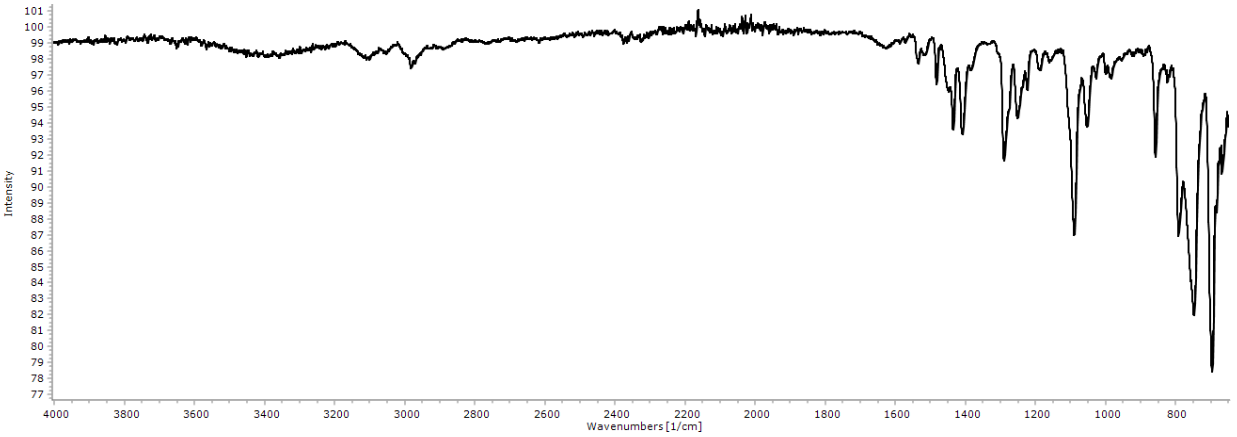

Figure S4. Solid-state IR spectrum (650-4000 cm^{-1}) of **4.**

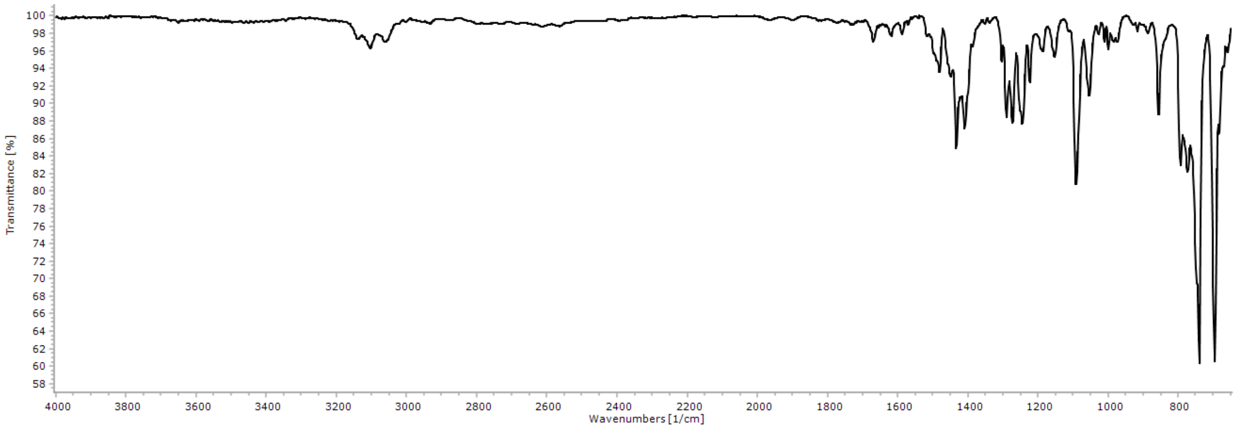

Figure S5. Solid-state IR spectrum (650-4000 cm^{-1}) of **5.**

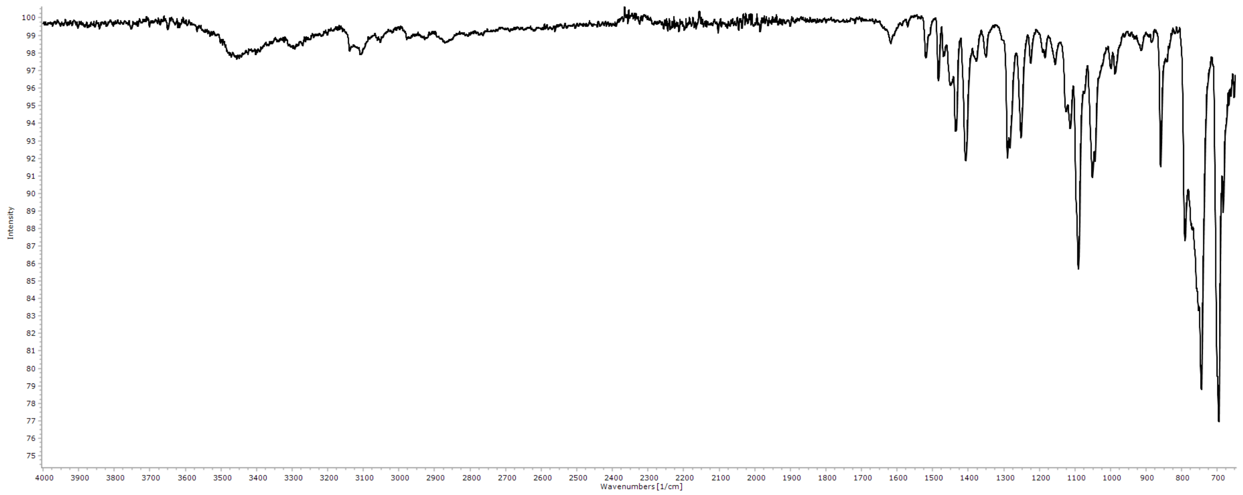

Figure S6. Solid-state IR spectrum (650-4000 cm^{-1}) of **6.**

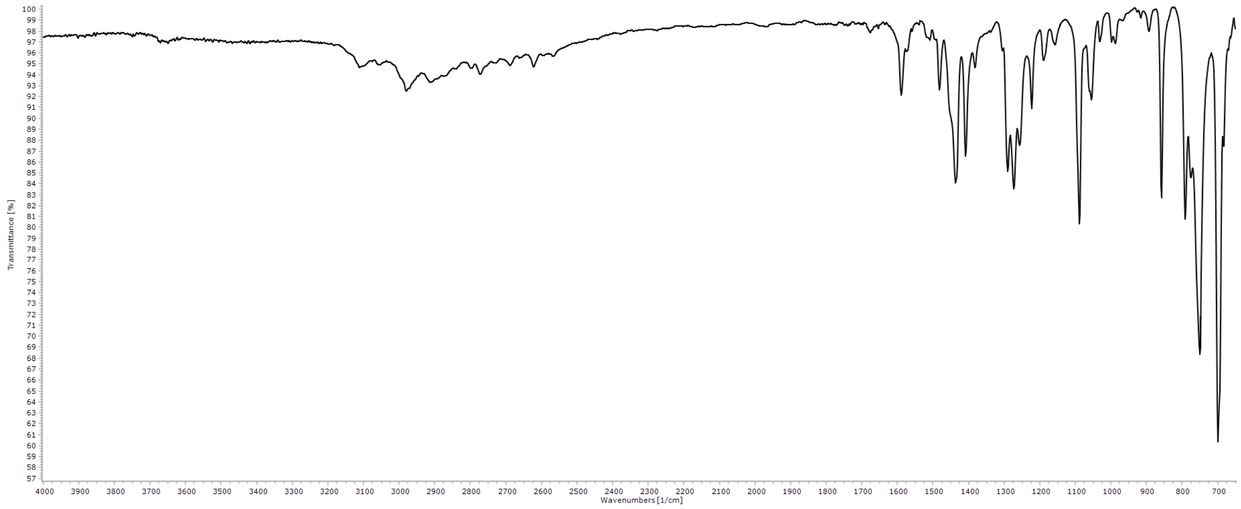

Figure S7. Solid-state IR spectrum (650-4000 cm^{-1}) of **7.**

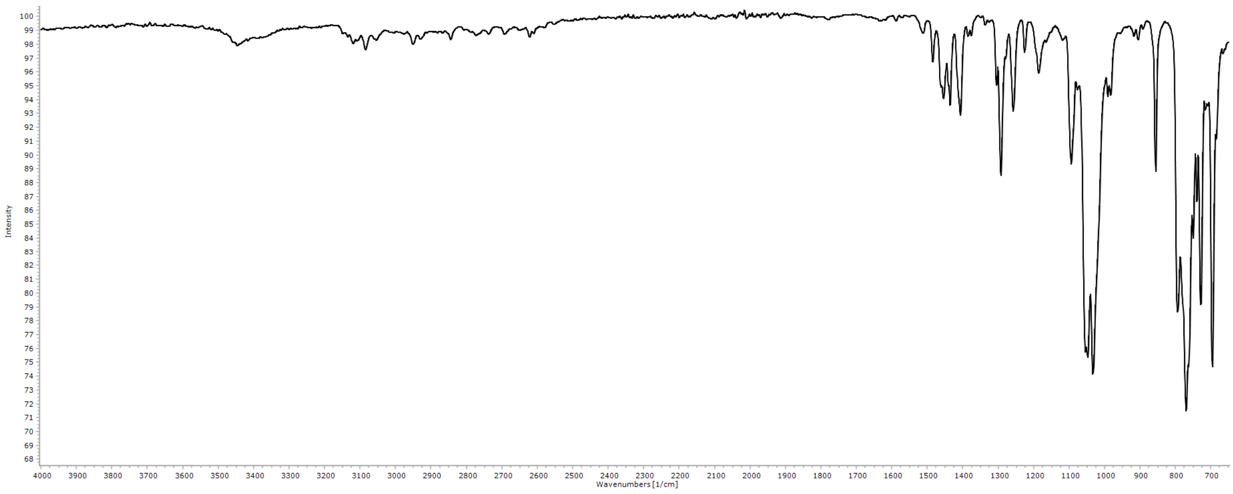

Figure S8. Solid-state IR spectrum (650-4000 cm^{-1}) of **8.**

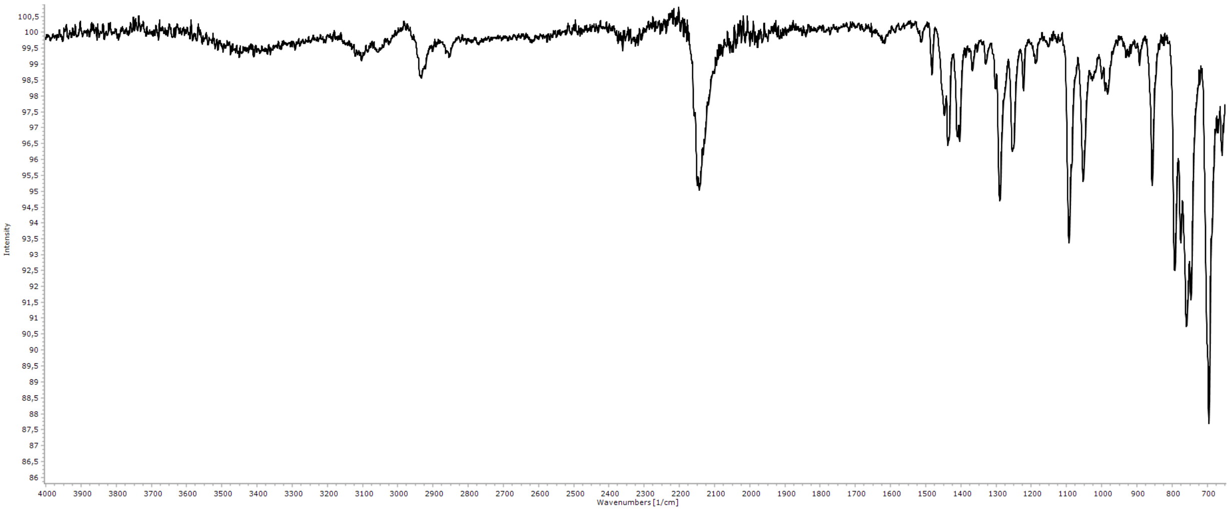

Figure S9. Solid-state IR spectrum (650-4000 cm^{-1}) of **9.**

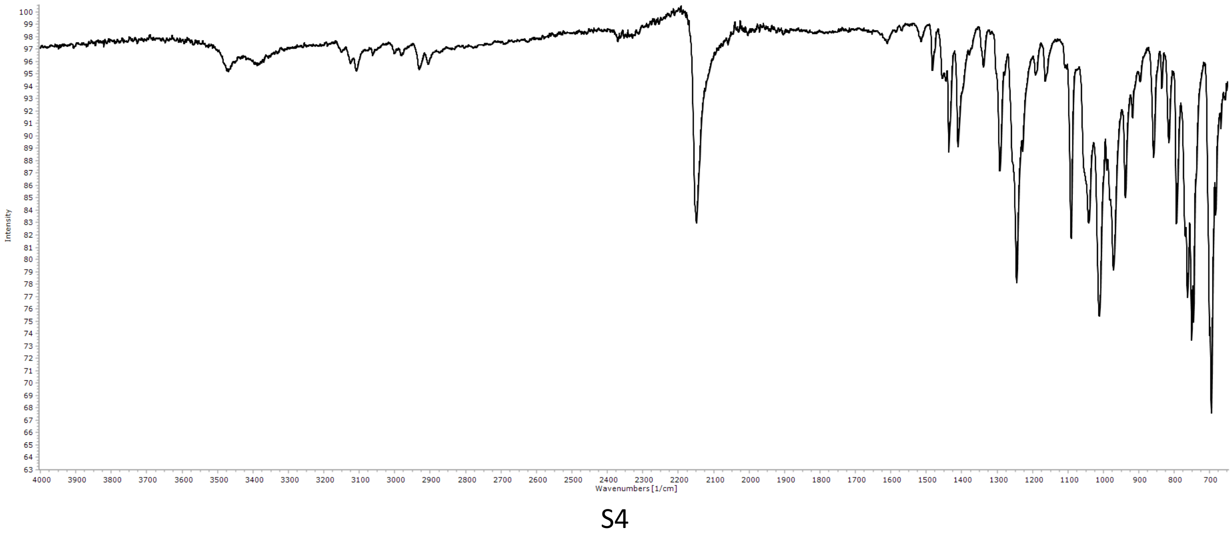

NMR spectra

Figure S10. ^{1}H NMR spectrum (401 MHz, $CDCl_3$) of **1**.

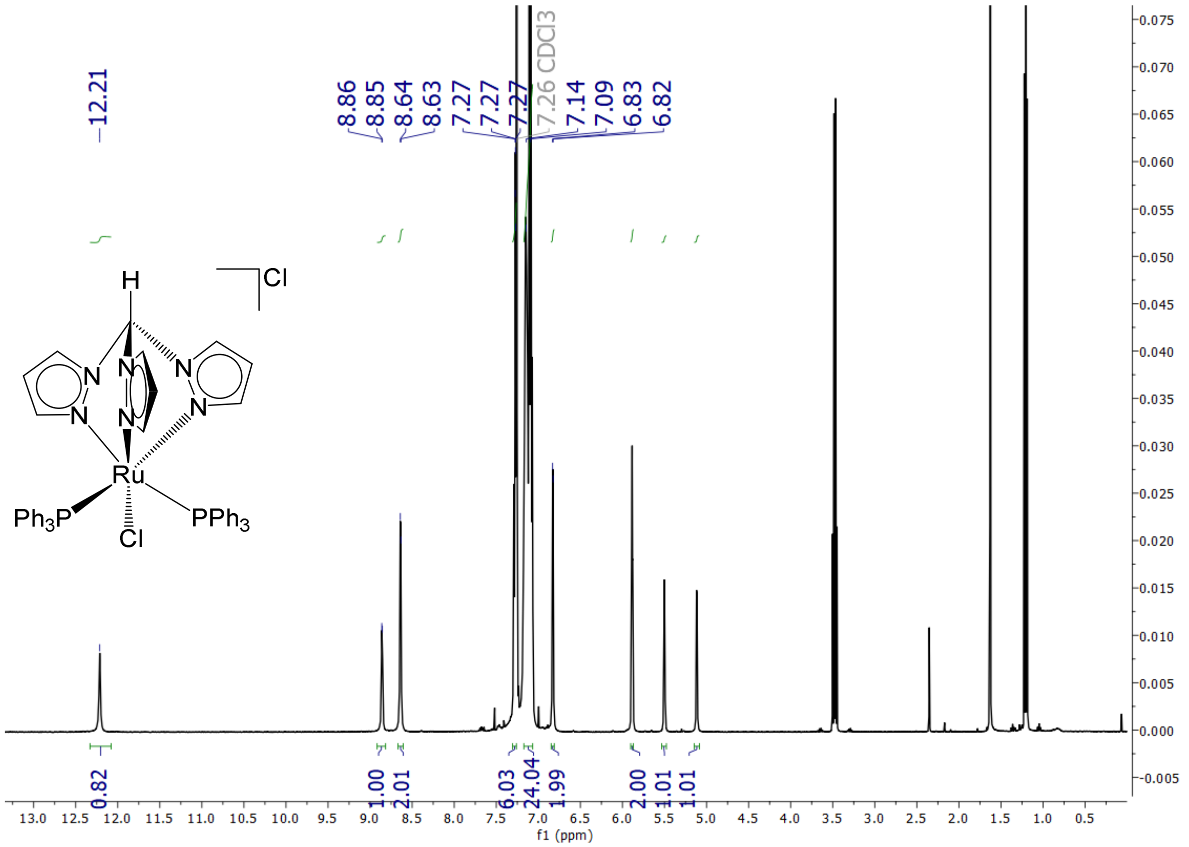

Figure S11. ^{31}P NMR spectrum (162 MHz, $CDCl_3$) of **1**.

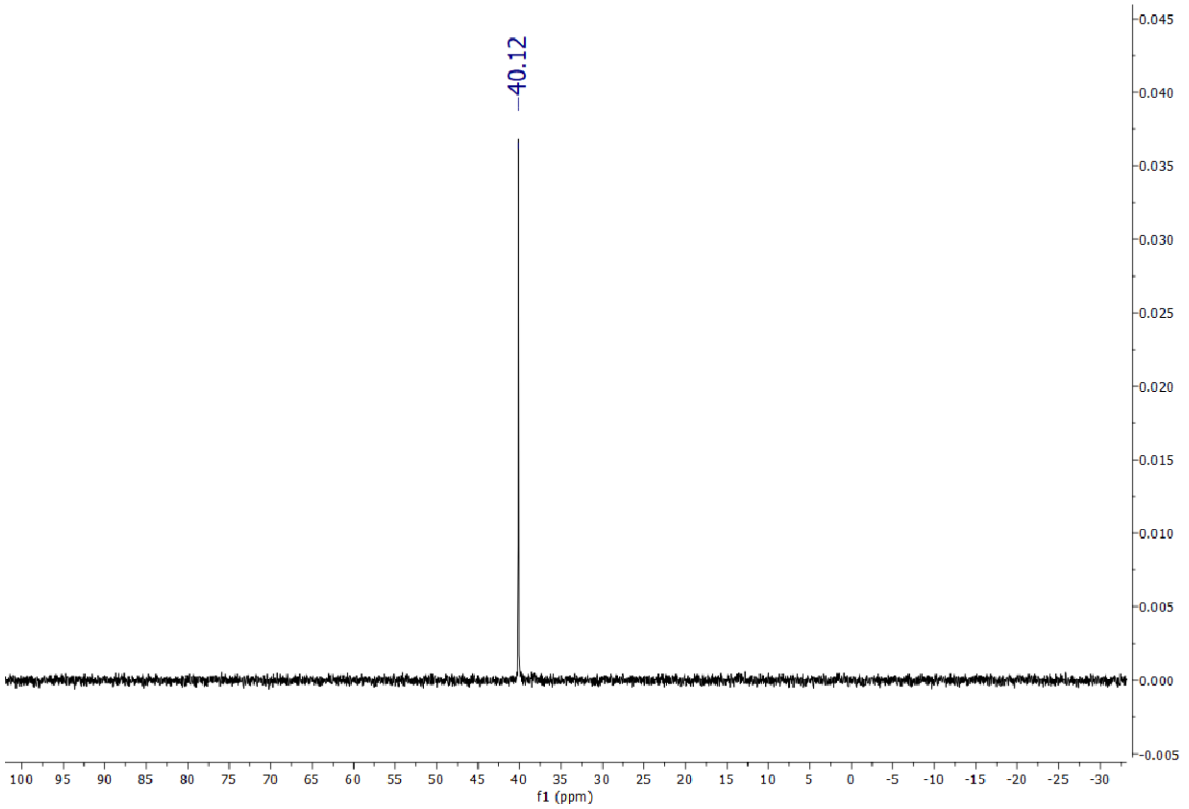

Figure S12. ^{1}H NMR spectrum (501 MHz, $CDCl_3$) of **2**

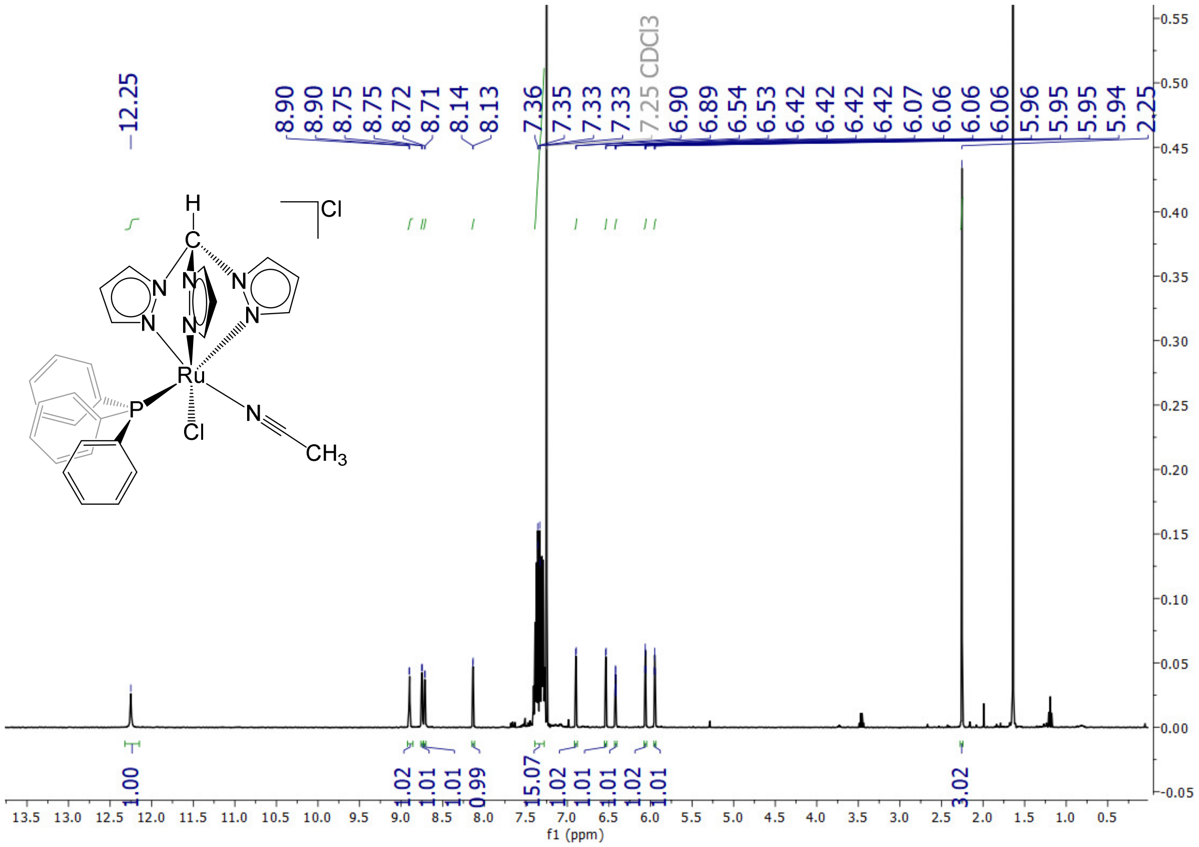

Figure S13. ^{13}C NMR spectrum (126 MHz, $CDCl_3$) of **2**

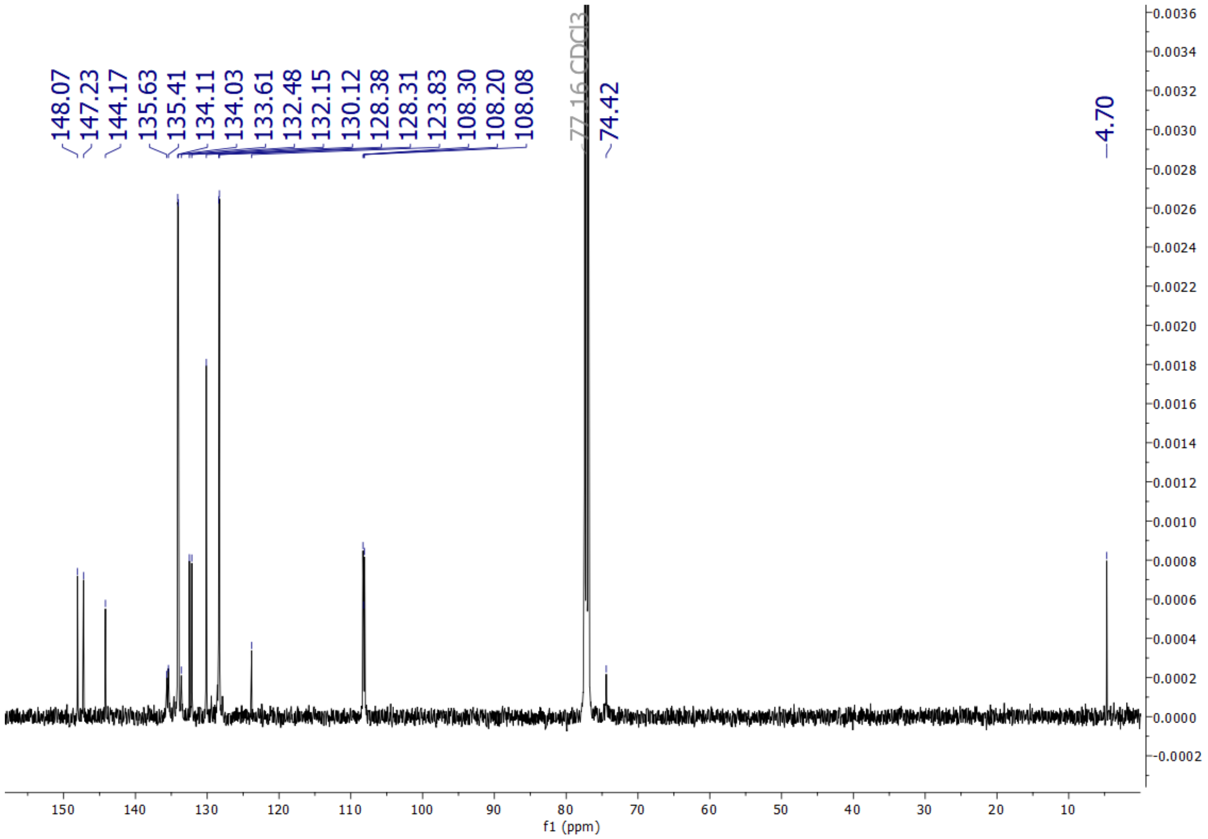

Figure S14. ^{31}P NMR spectrum (202 MHz, $CDCl_3$) of **2**

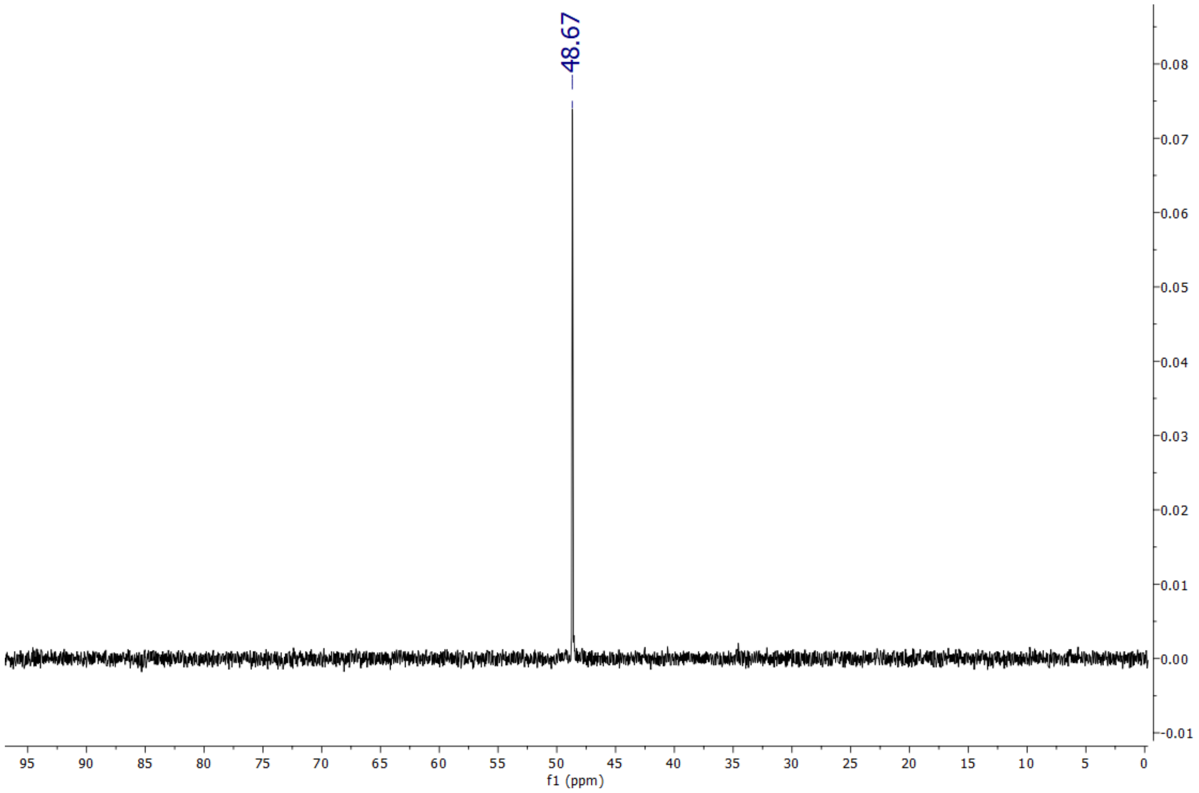

Figure S15. ^{1}H NMR spectrum (501 MHz, $CDCl_3$) of **3**

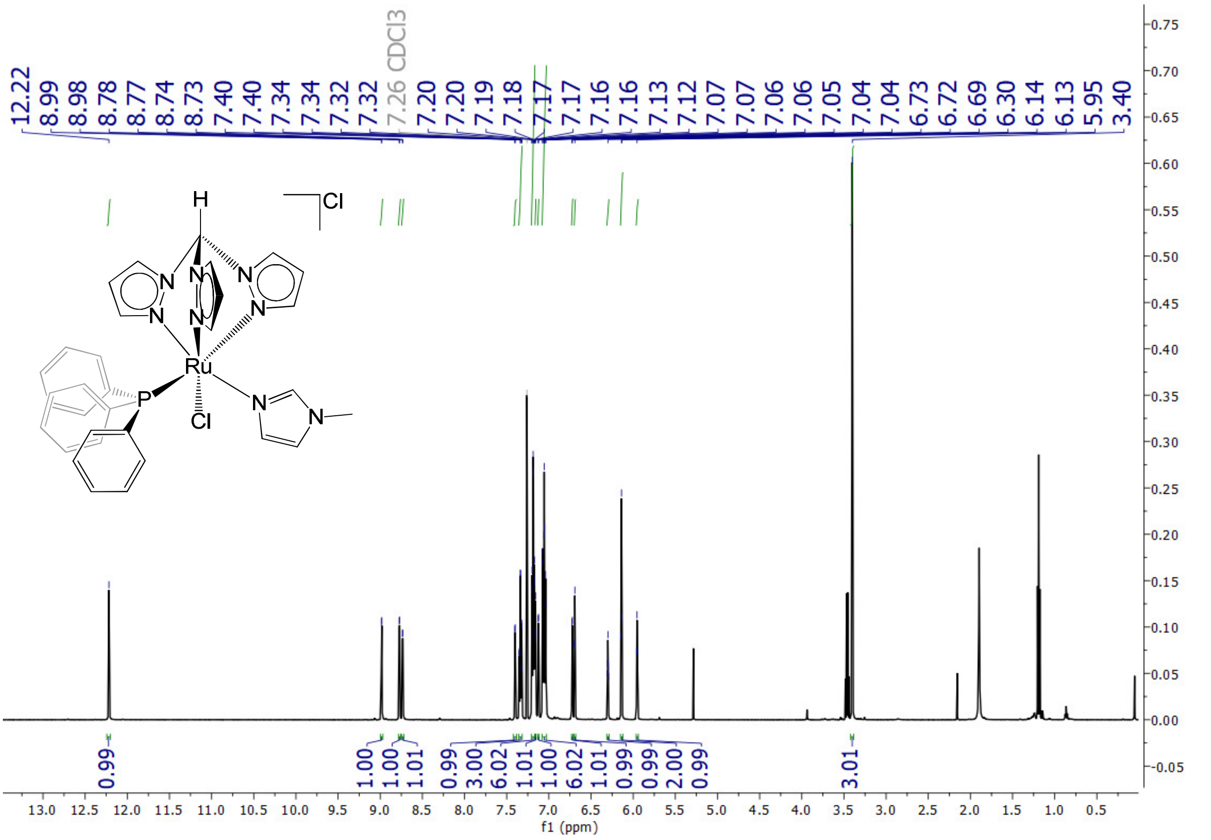

Figure S16. ^{13}C NMR spectrum (126 MHz, $CDCl_3$) of **3**

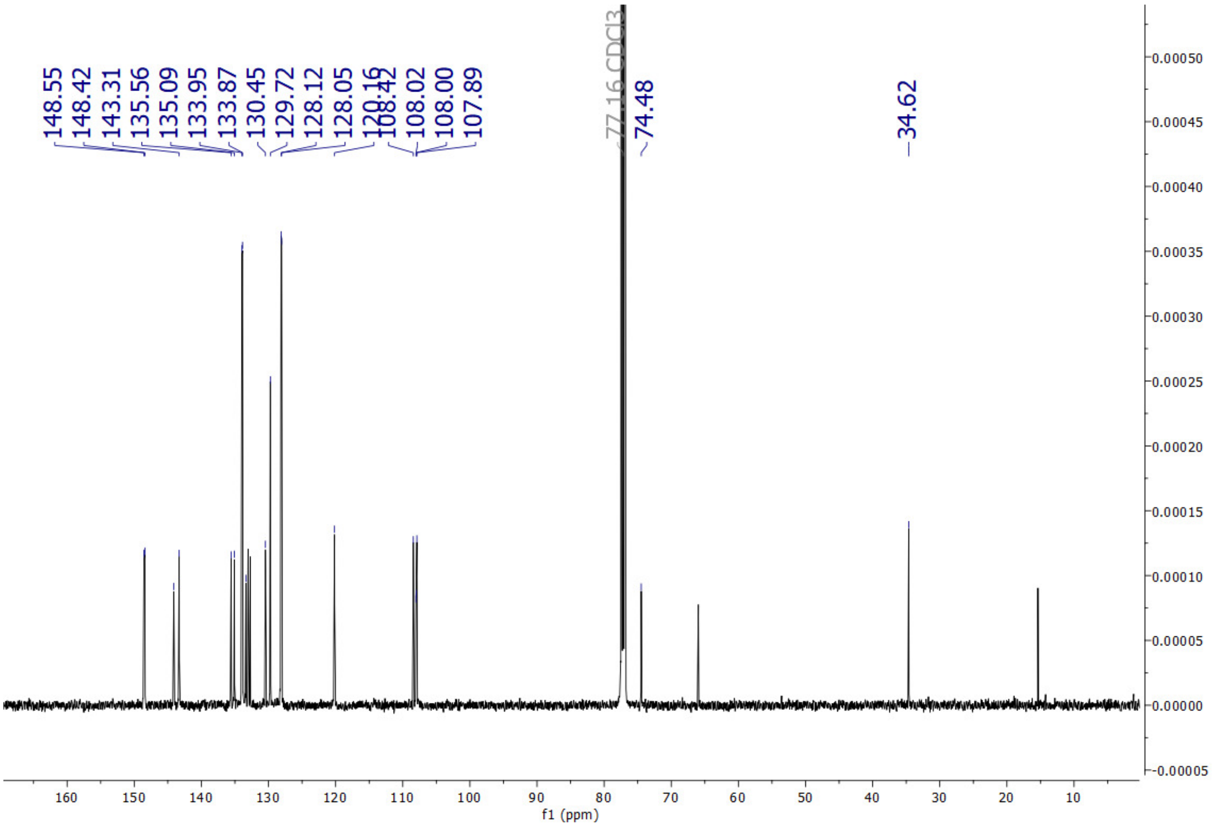

Figure S17. ^{31}P NMR spectrum (202 MHz, $CDCl_3$) of **3**

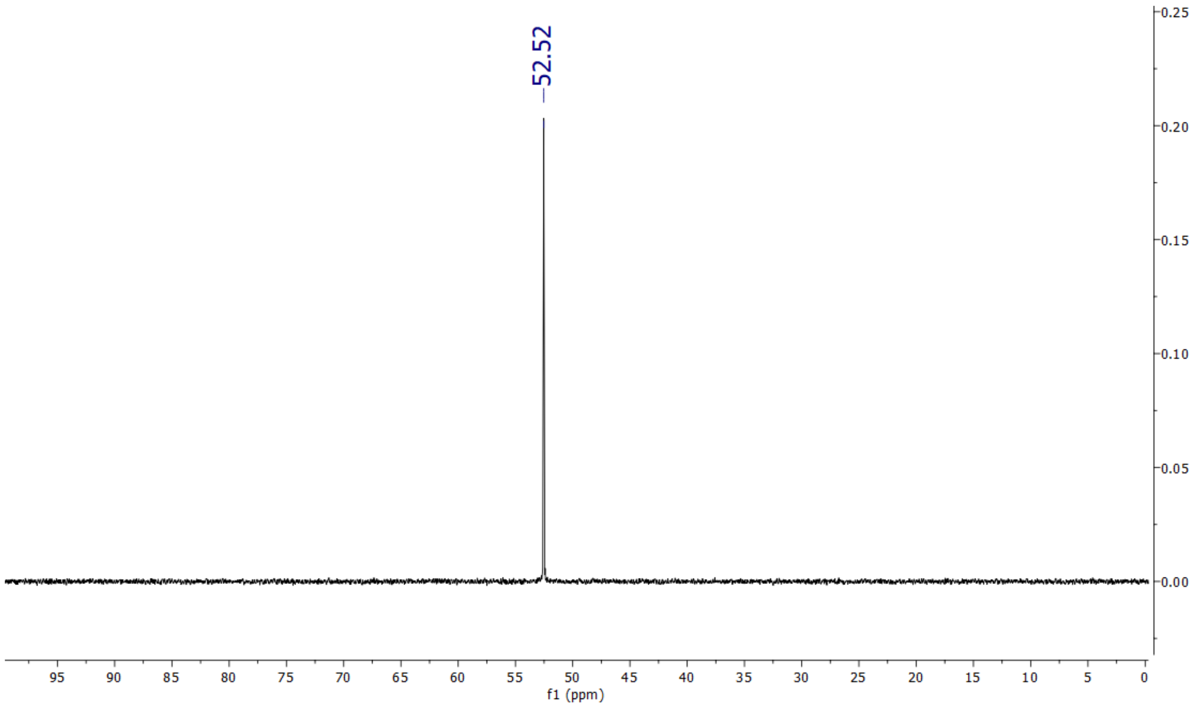

Figure S18. ^{1}H NMR spectrum (501 MHz, $CDCl_3$) of **4**

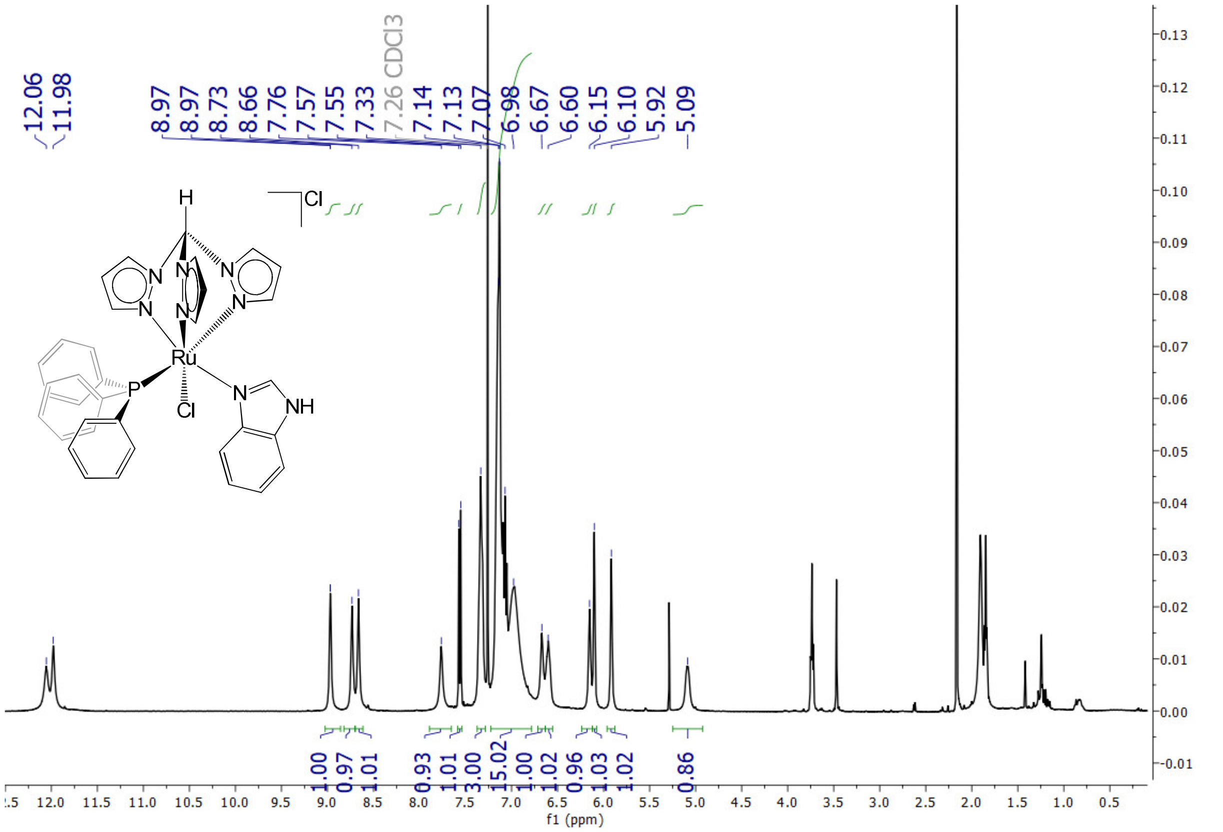

Figure S19. ^{13}C NMR spectrum (126 MHz, $CDCl_3$) of **4**

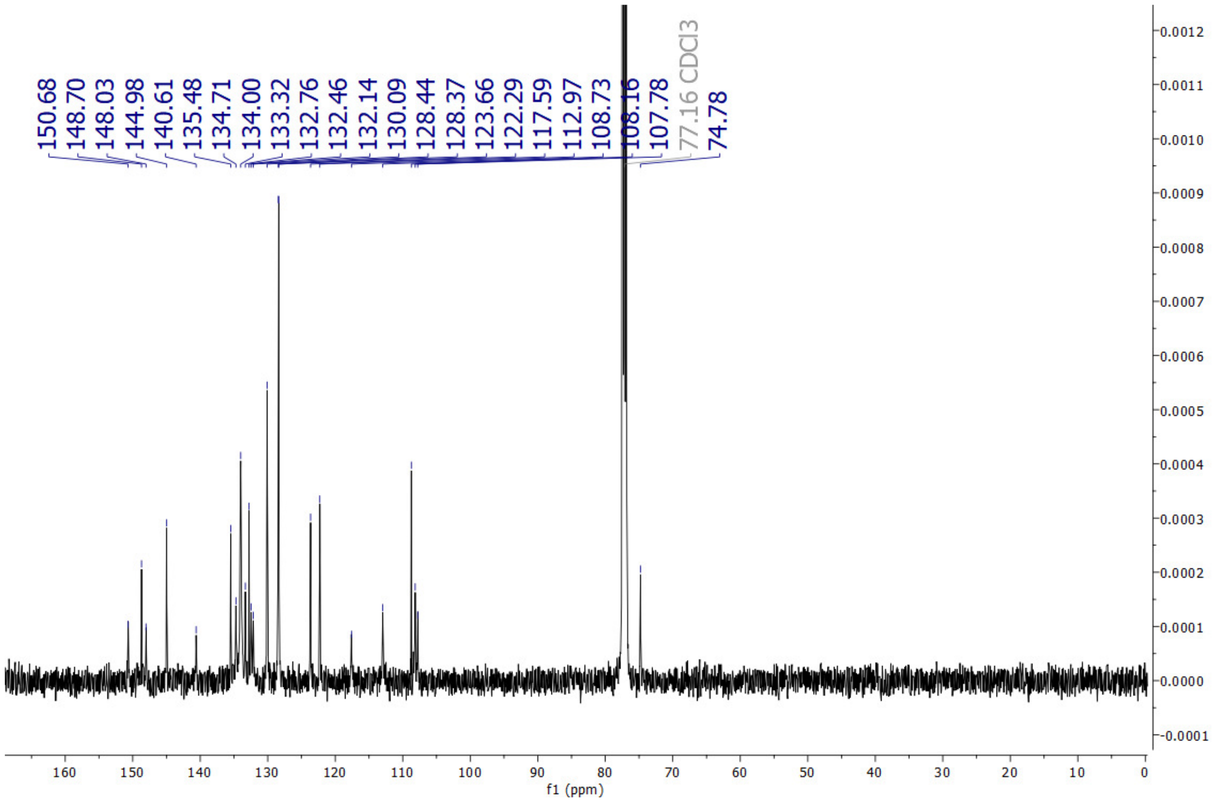

Figure S20. ^{31}P NMR spectrum (202 MHz, $CDCl_3$) of **4**

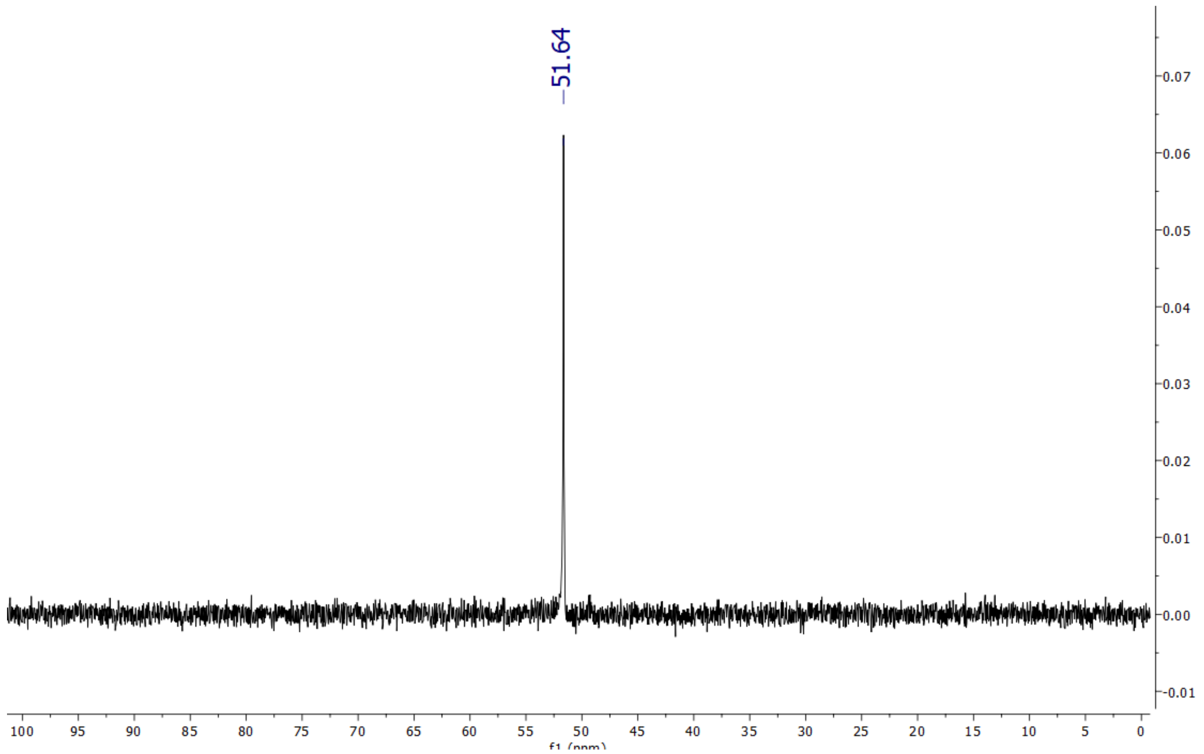

Figure S21. ^{1}H NMR spectrum (501 MHz, $CDCl_3$) of **5**

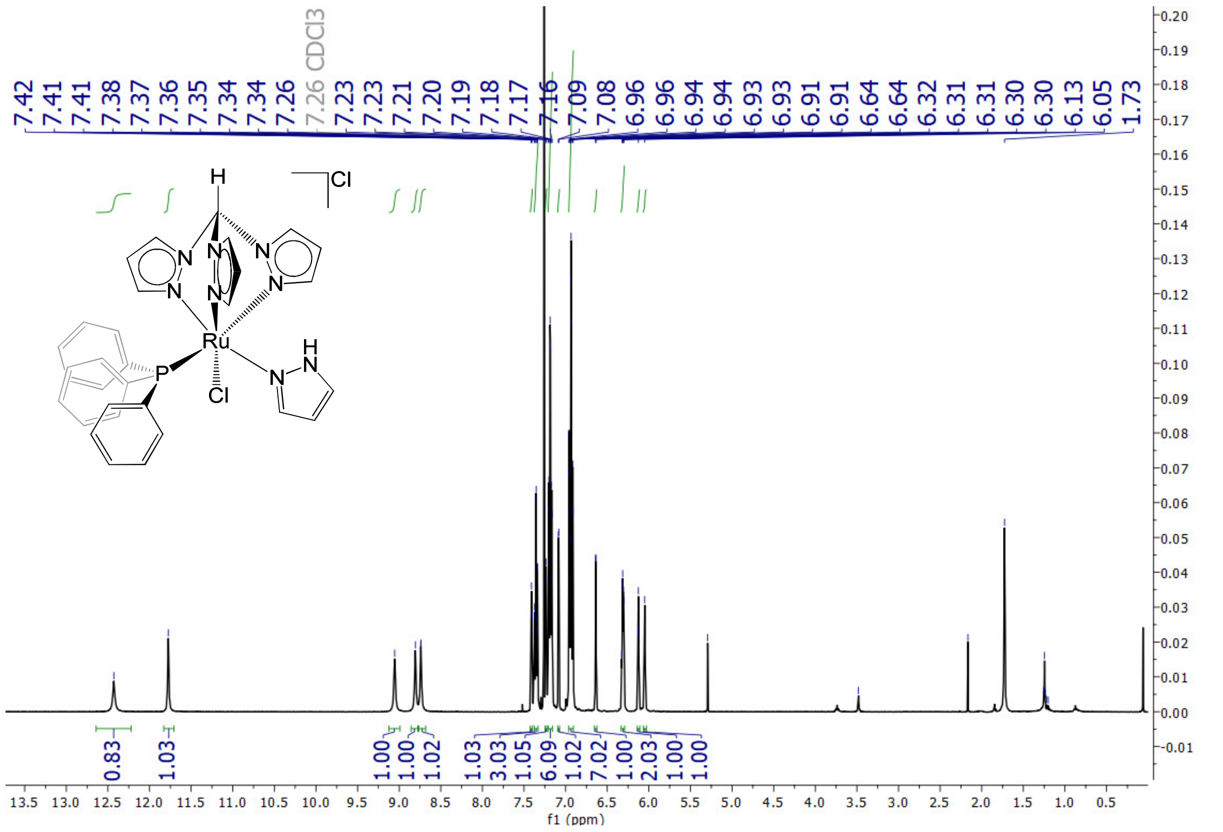

Figure S22. ^{13}C NMR spectrum (126 MHz, $CDCl_3$) of **5**

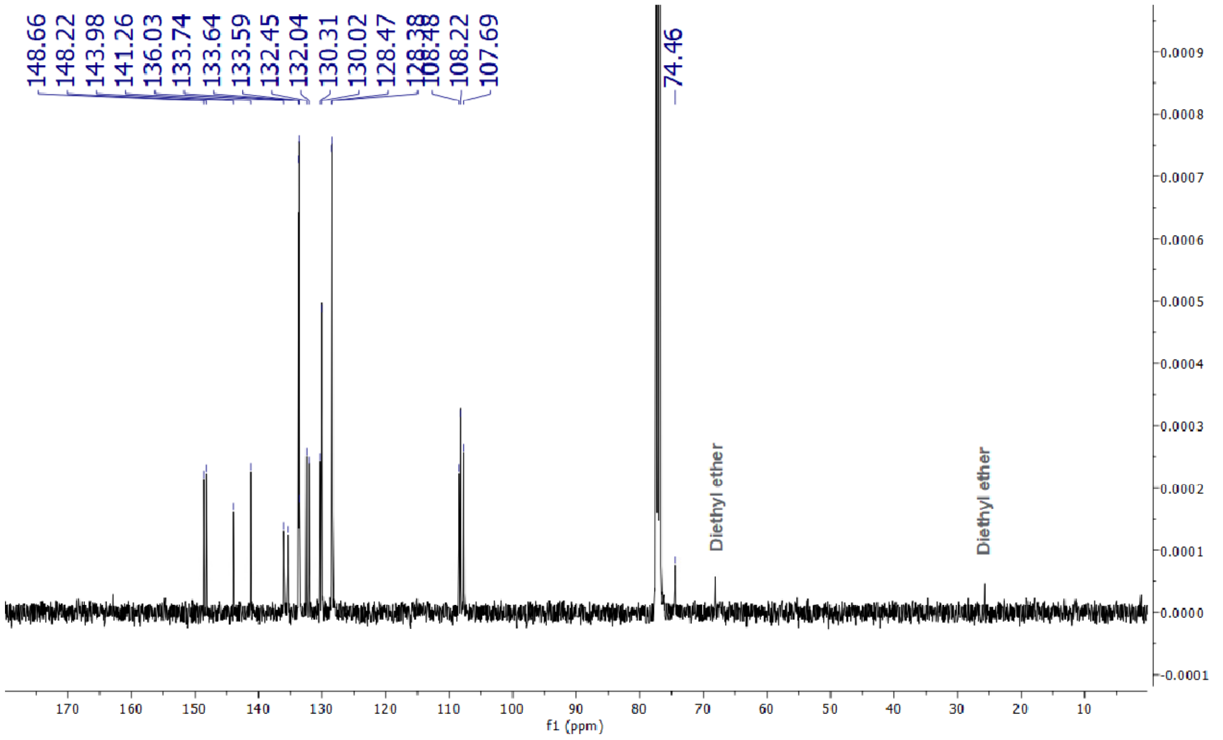

Figure S23. ^{31}P NMR spectrum (202 MHz, $CDCl_3$) of **5**

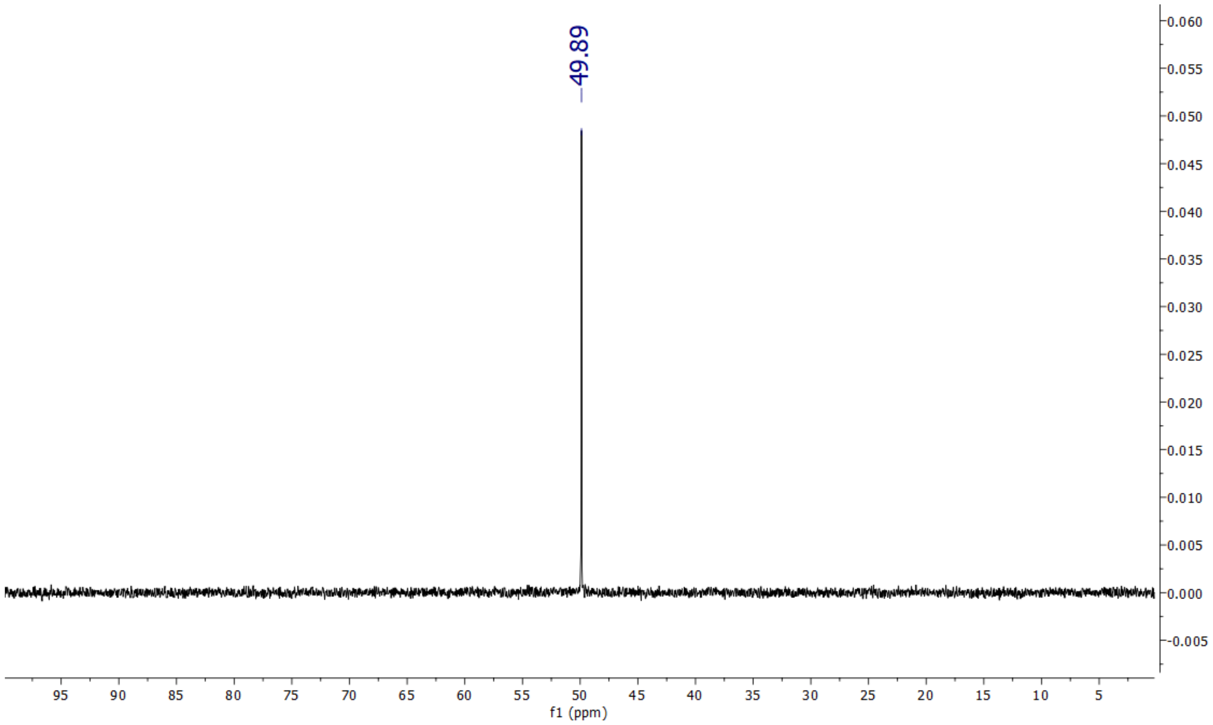

Figure S24. ^{1}H NMR spectrum (501 MHz, $CDCl_3$) of **6**

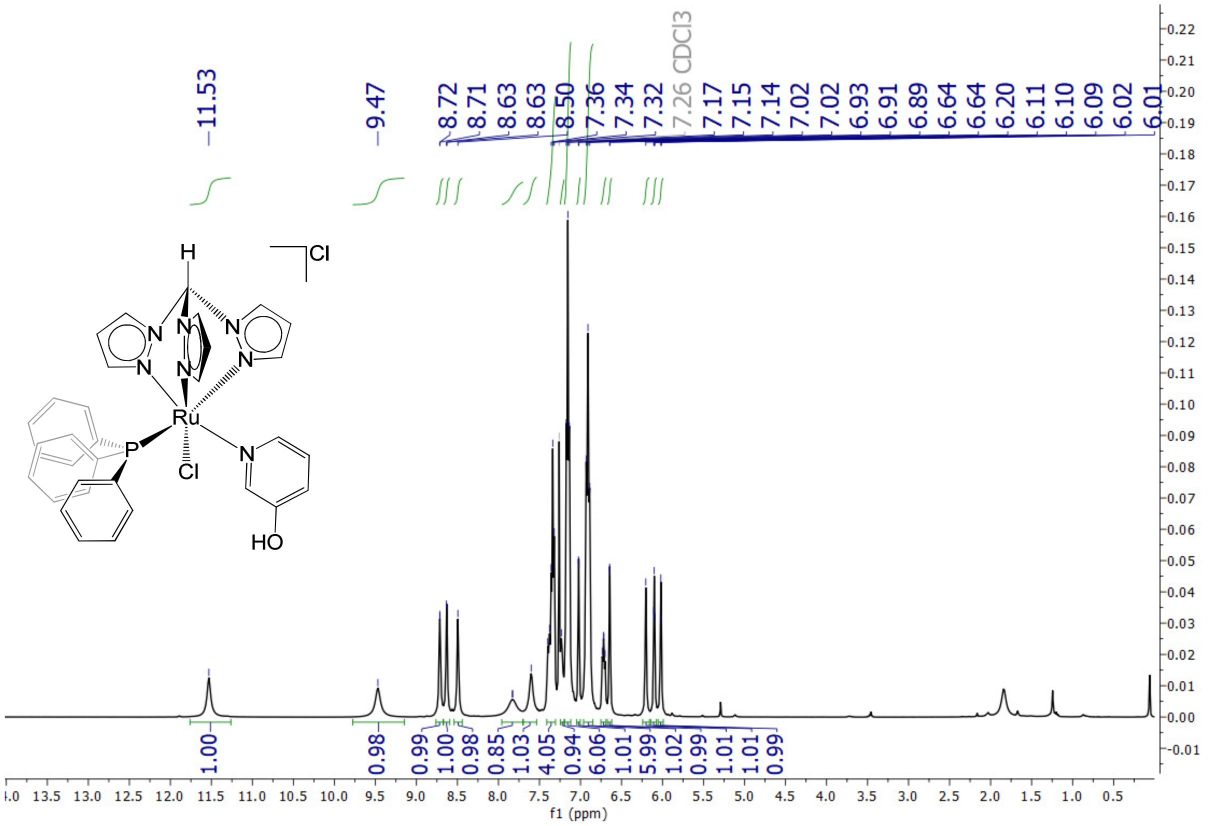

Figure S25. ^{13}C NMR spectrum (126 MHz, $CDCl_3$) of **6**

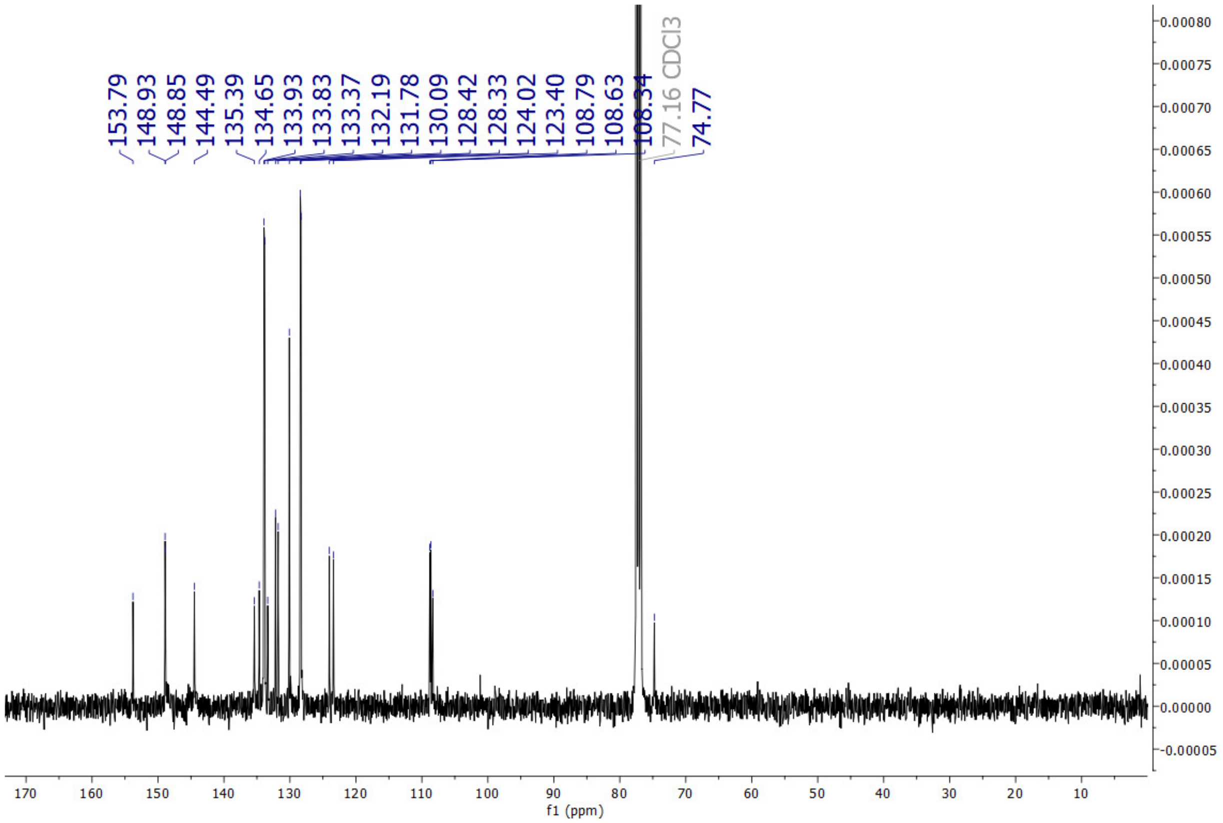

Figure S26. ^{31}P NMR spectrum (202 MHz, $CDCl_3$) of **6**

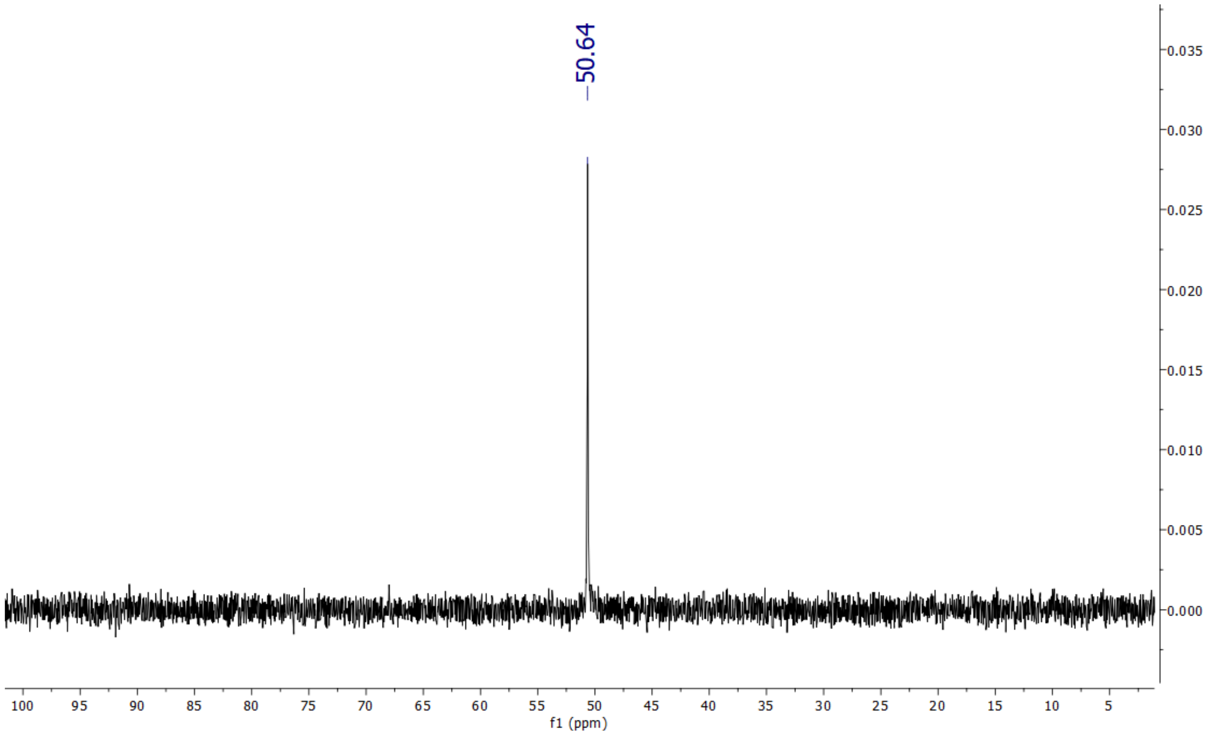

Figure S27. ^{1}H NMR spectrum (501 MHz, $CDCl_3$) of **7**

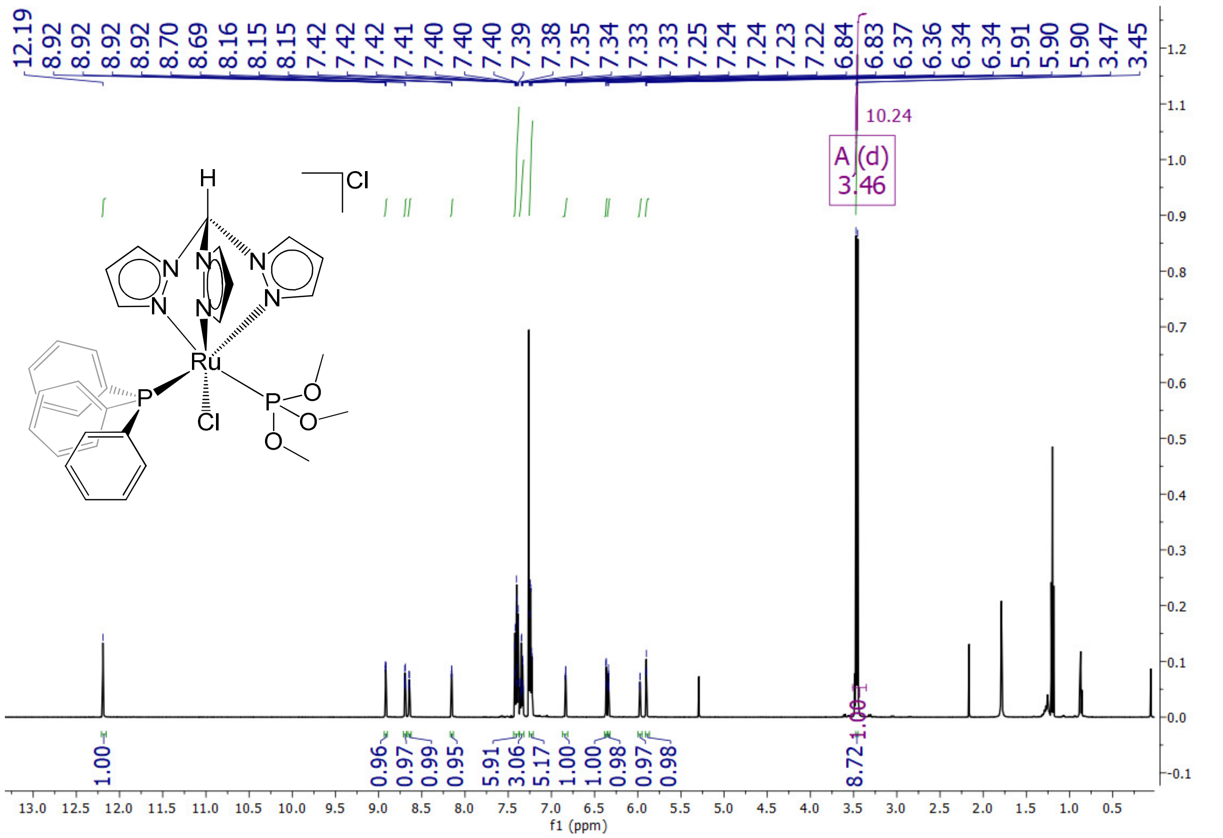

Figure S28. ^{13}C NMR spectrum (126 MHz, $CDCl_3$) of **7**

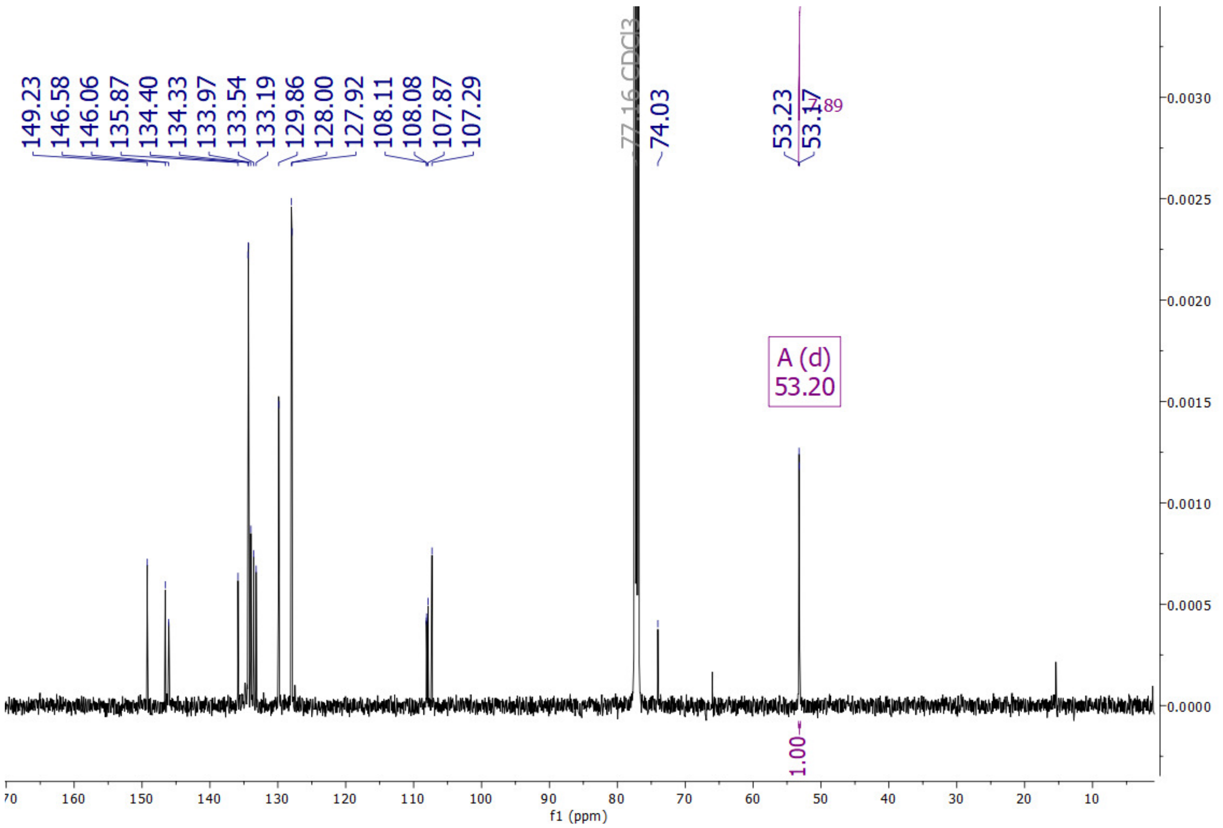

Figure S29. ^{31}P NMR spectrum (202 MHz, $CDCl_3$) of **7**

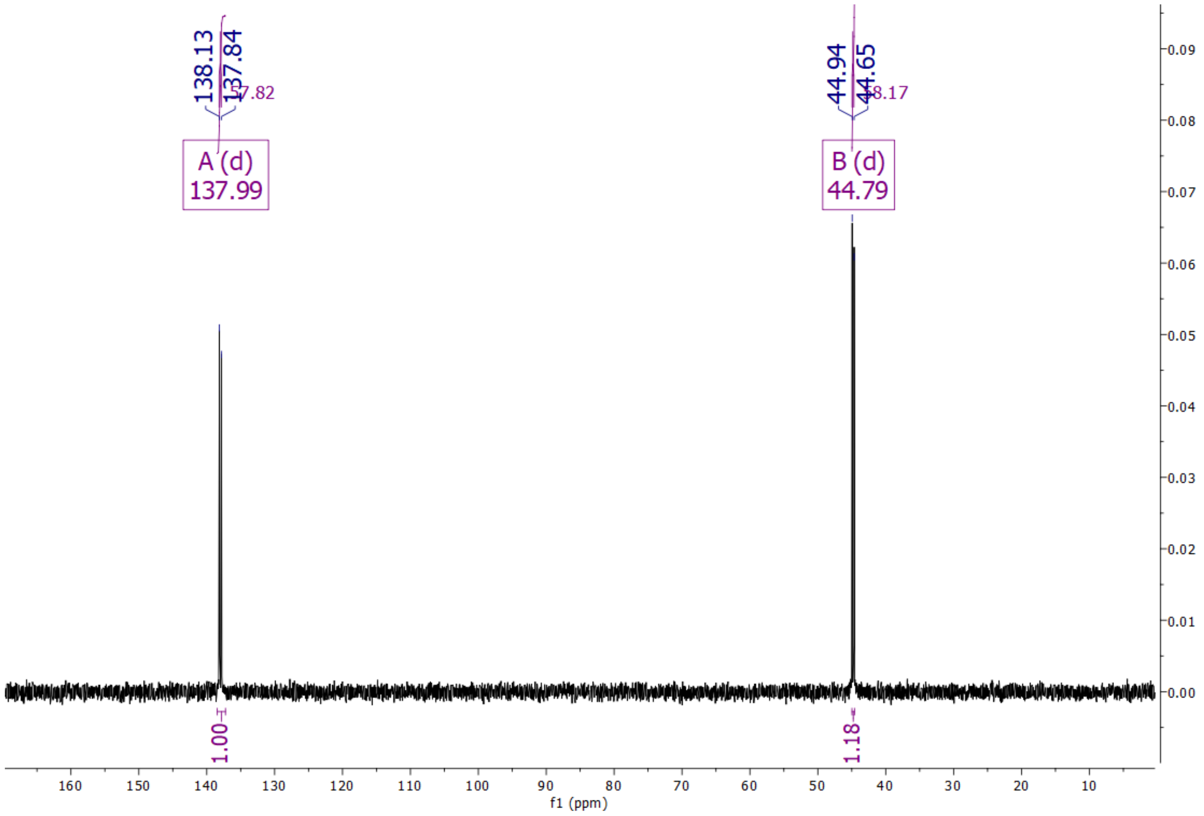

Figure S30. ^{1}H NMR spectrum (501 MHz, $CDCl_3$) of **8**

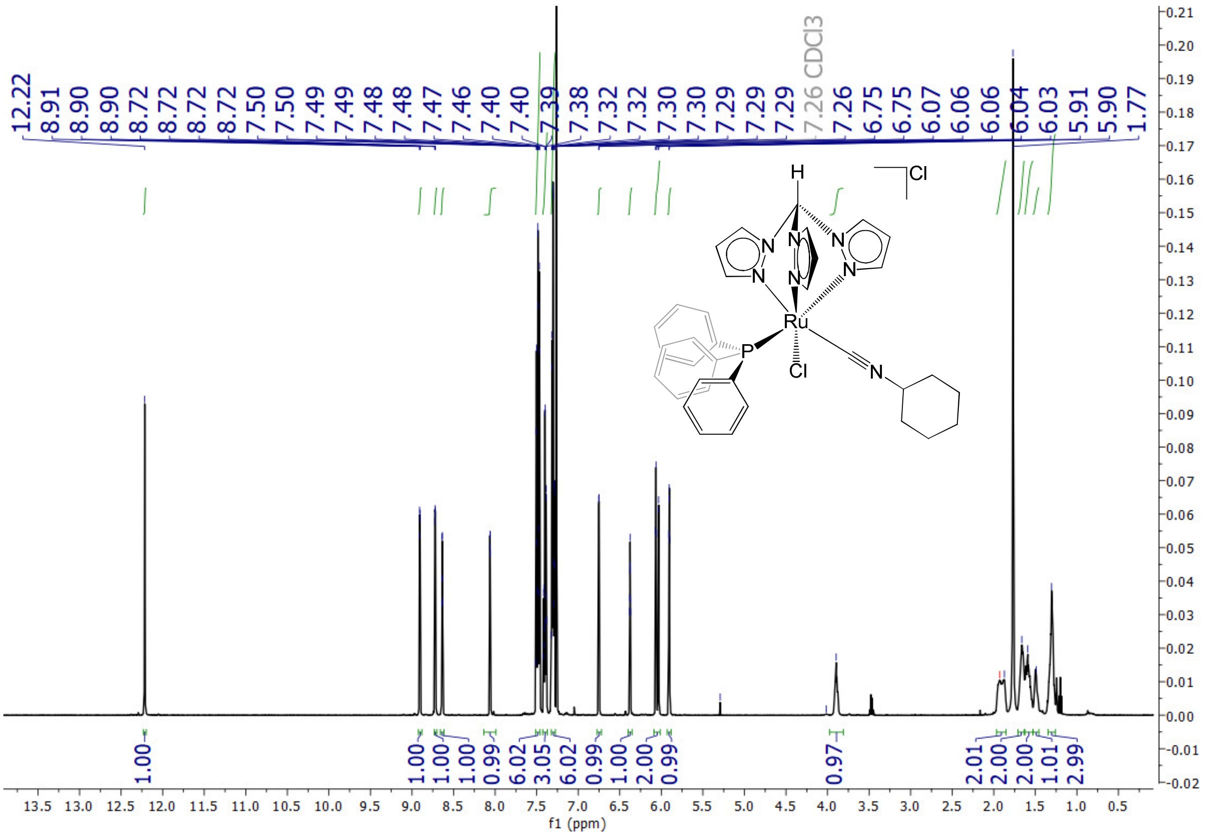

Figure S31. ^{13}C NMR spectrum (500 MHz, $CDCl_3$) of **8**

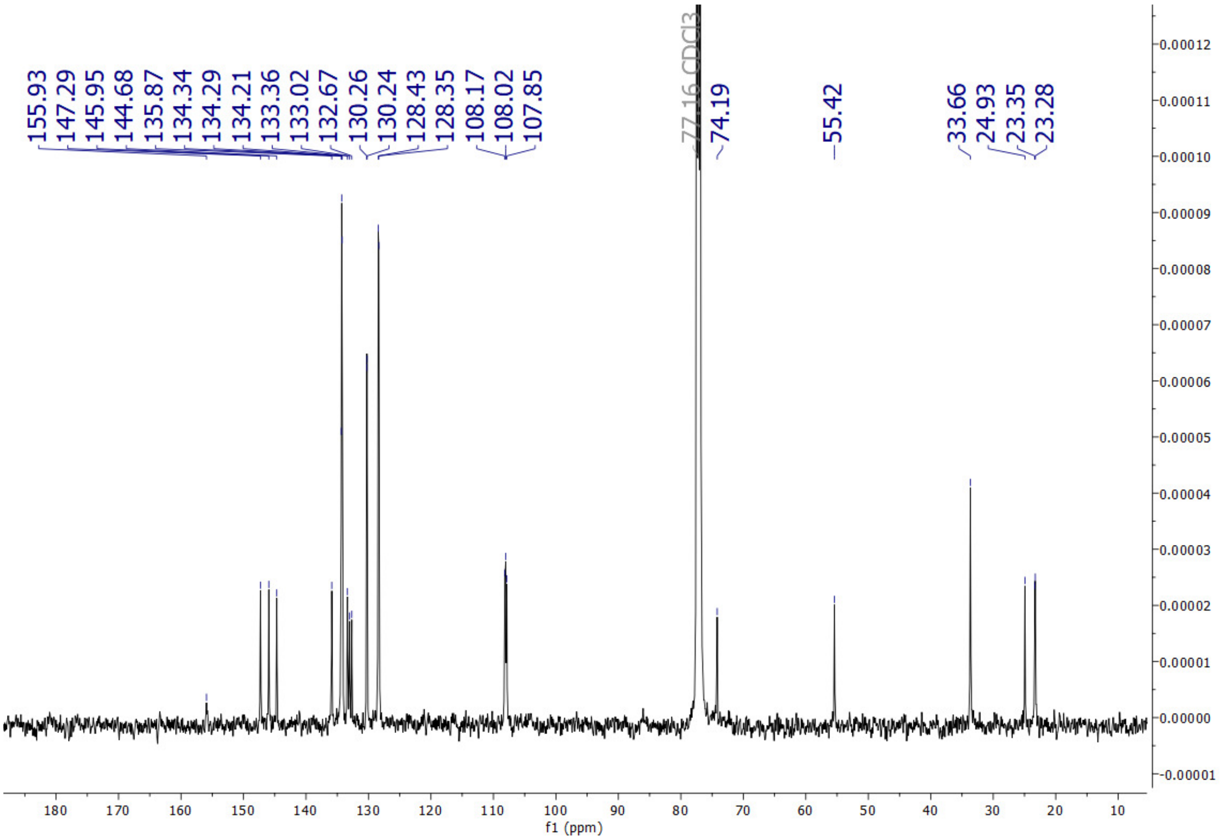

Figure S32. ^{31}P NMR spectrum (202 MHz, $CDCl_3$) of **8**

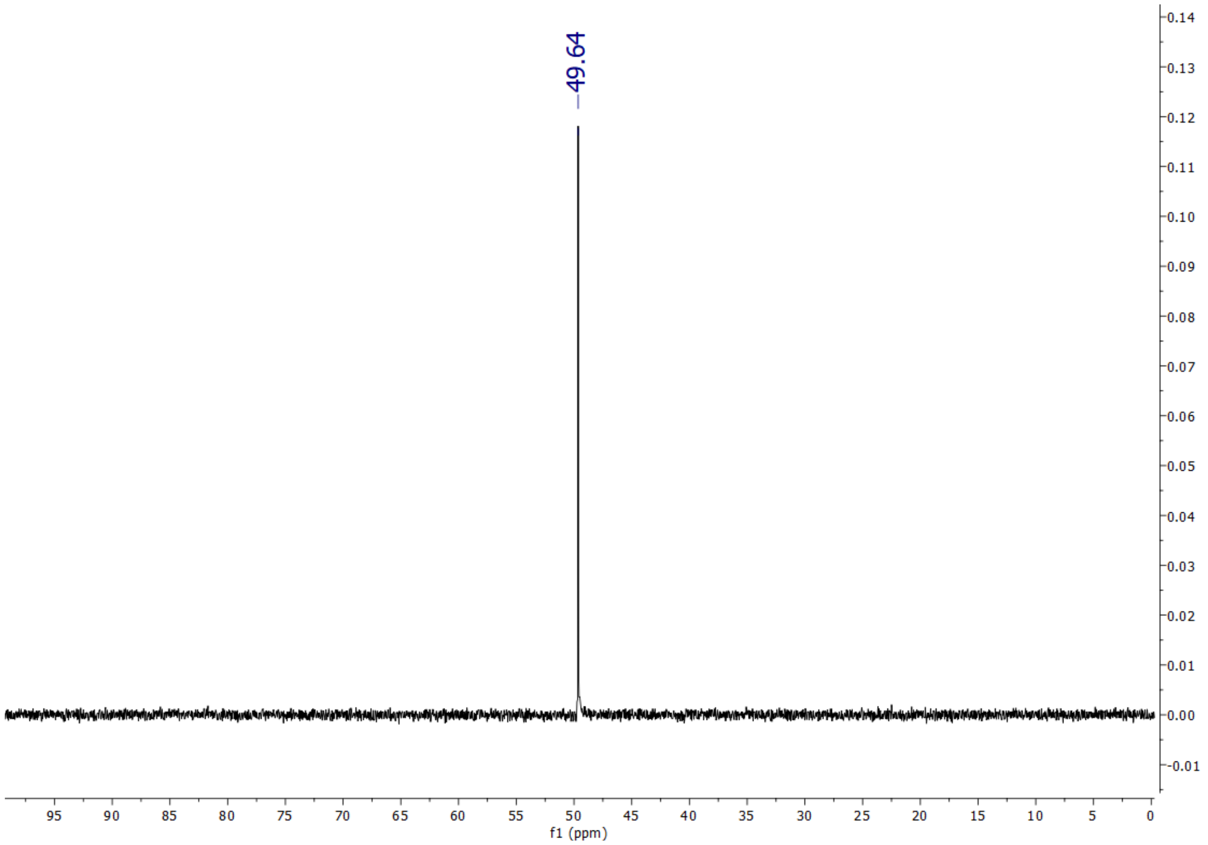

Figure S33. ^{1}H NMR spectrum (401 MHz, $CDCl_3$) of **9**

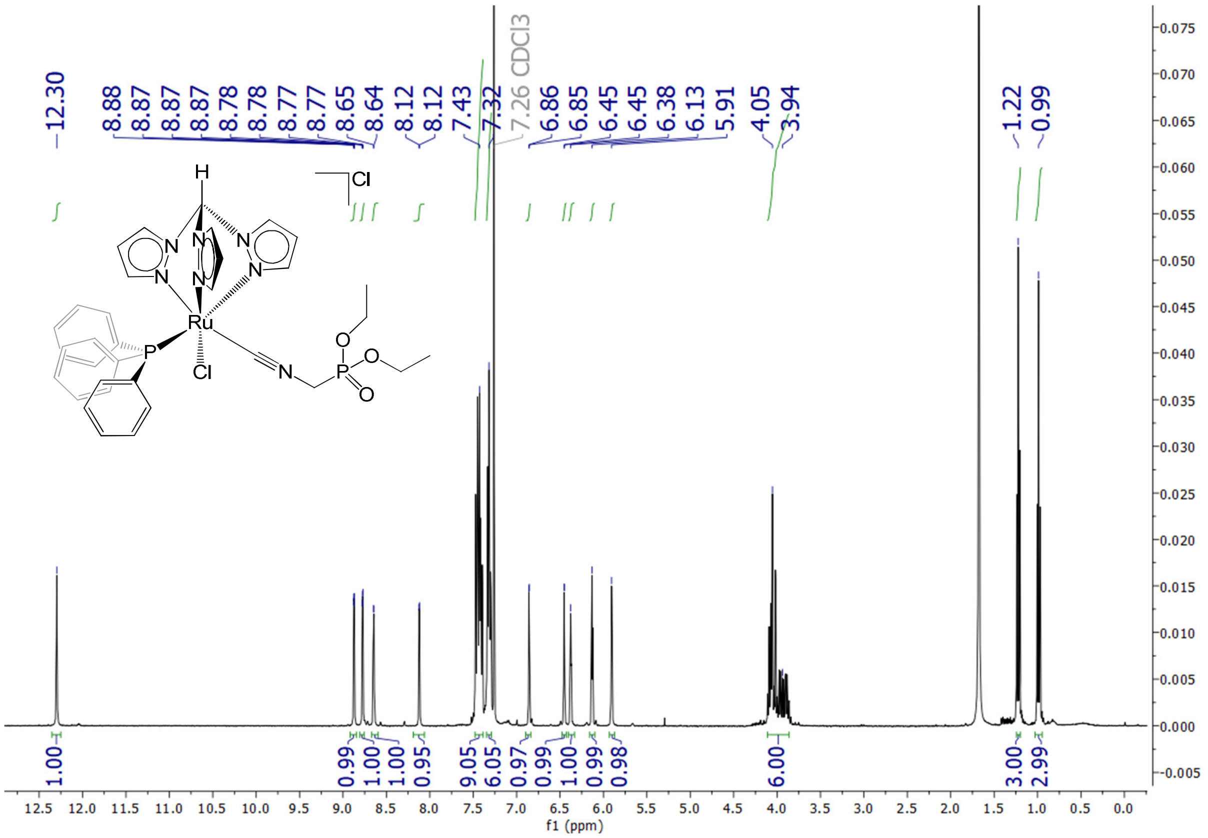

Figure S34. ^{13}C NMR spectrum (126 MHz, $CDCl_3$) of **9**

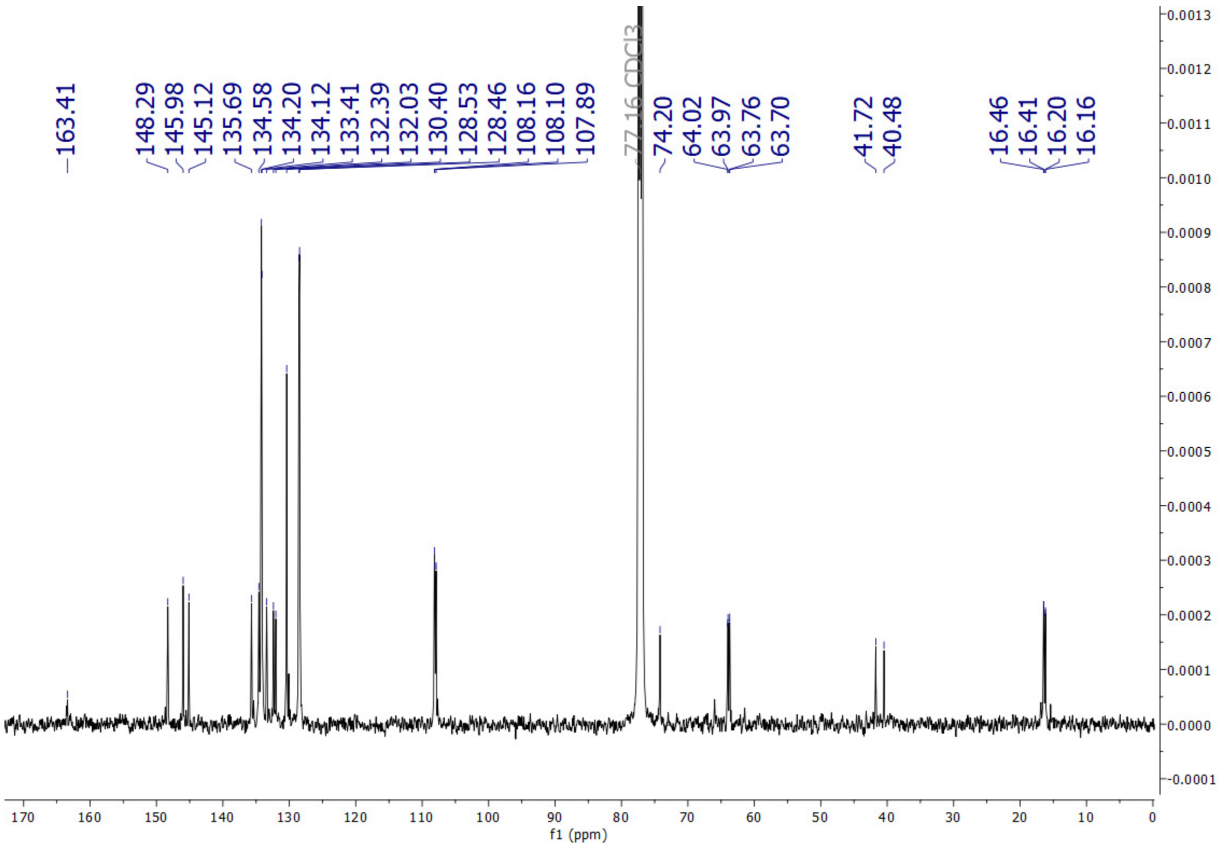

Figure S35. ^{31}P NMR spectrum (202 MHz, $CDCl_3$) of **9**

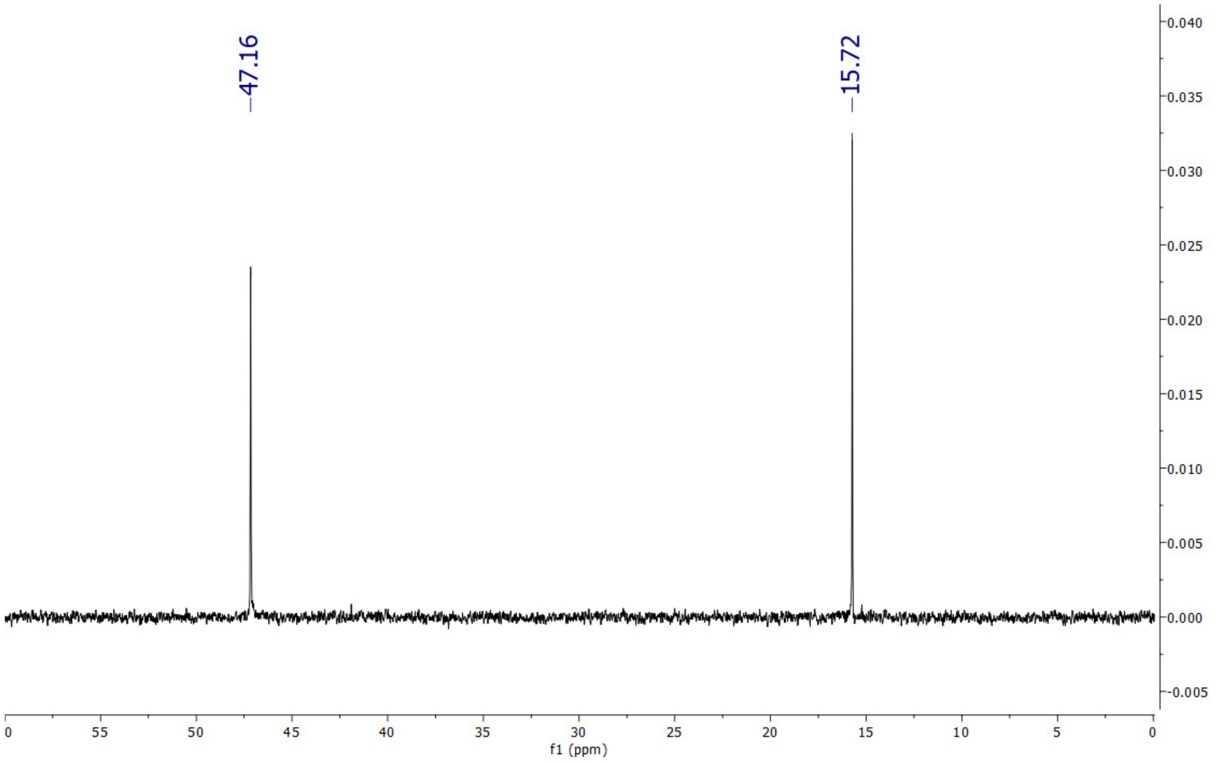

NMR data of complexes in D_2O[1]

Figure S36. Complex [RuCl(κ^3-tpm)(PPh_3)(NCMe)]Cl, **2**, in D_2O

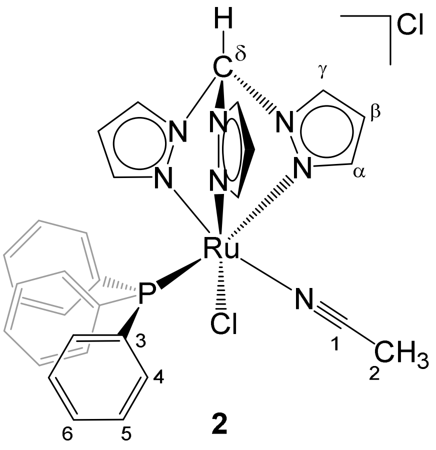

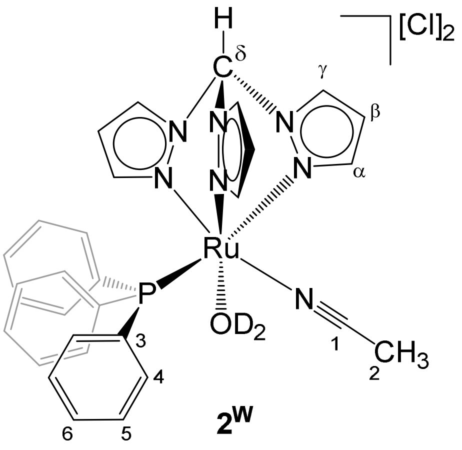

^{1}H NMR (D_2O): δ/ppm (**2**) = 8.41, 8.36, 8.31 (d-br, 3H, C^{γ}H); 8.22 (d-br, 1H, C^{α}H); 7.53 (t-br, 3H, C^6H); 7.39-7.32 (m, 12H, C^4H + C^5H); 7.10, 6.94 (d-br, 1H, C^{α}H); 6.65, 6.30, 6.17 (t, 3H, C^{β}H); 2.35 (s, 3H, C^2H). δ/ppm (**2^W**) = 8.44, 8.41, 8.36 (d-br, 3H, C^{γ}H); 8.19 (d-br, 1H, C^{α}H); 7.57 (t-br, 3H, C^6H); 7.44, 7.23 (t-br, 12H, C^4H + C^5H); 7.04 (d-br, 1H, C^{α}H); 6.97 (d-br, 1H, C^{α}H); 6.69, 6.32, 6.22 (t, 3H, C^{β}H); 2.39 (s, 3H, C^2H). *C^{δ}H not observed.* $^{31}P\{^1H\}$ NMR (D_2O): δ/ppm = 48.5 (**2^W**), 47.6 (**2**). **2^W/2** ratio (from ^{1}H NMR) = 5.1 (t_0), 11.6 (after 48h). In DMSO-d_6/DMEM-d (0.14/0.56 mL): **2^W/2** ratio (from ^{31}P NMR) = 0 (t_0), 1.5 (after 24h). Two additional signals were detected in the ^{31}P NMR spectrum (δ/ppm = 37.5, 37.2 ppm).

[1] Preparation of each sample required 2 hours (t_0) stirring of a suspension of each complex in D_2O (see manuscript).

Figure S37. Complex $[RuCl(\kappa^3\text{-tpm})(PPh_3)\{NCHN(Me)(CH)_2\}]Cl$, **3**, in D_2O

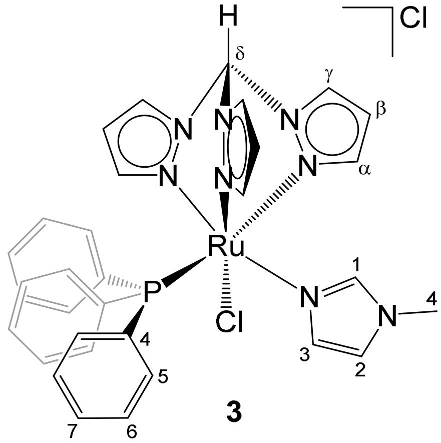

3

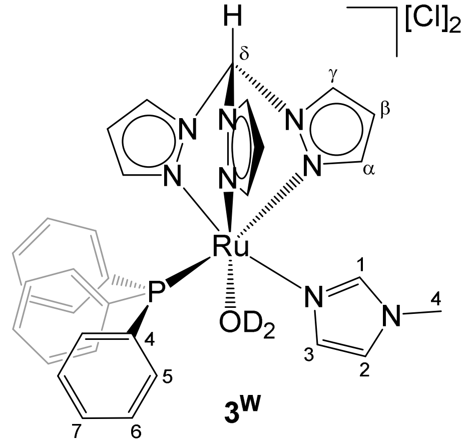

3^W

^{1}H NMR (D_2O): δ/ppm (**3**) = 8.41, 8.35, 8.35 (d-br, 3H, $C^{\gamma}H$); 7.53-7.45 (m, 3H, $C^7H + C^{\alpha}H$); 7.28 (t-br, 6H, C^6H or C^5H); 7.14 (d-br, 1H, $C^{\alpha}H$); 7.05-6.94 (m, 8H, C^6H or $C^5H + C^{\alpha}H + 2\ CH^{Imid}$); 6.53, 6.40, 6.14 (t, 3H, $C^{\beta}H$); 6.34 (m, 1H, CH^{Imid}); 3.52 (s, 3H, C^4H). δ/ppm (**3^W**) = 8.46, 8.41, 8.39 (d-br, 3H, $C^{\gamma}H$); 7.53-7.45, 7.33 (m, 3H, $C^7H + C^{\alpha}H$); 7.33 (t-br, 6H, C^6H or C^5H); 7.08 (d-br, 1H, $C^{\alpha}H$); 7.01-6.94 (m, 8H, C^6H or $C^5H + C^{\alpha}H + 2\ CH^{Imid}$); 6.58, 6.44, 6.18 (t, 3H, $C^{\beta}H$); 6.34 (m, 1H, CH^{Imid}); 3.57 (s, 3H, C^4-H). *$C^{\delta}H$ not observed.* $^{31}P\{^1H\}$ NMR (D_2O): δ/ppm = 52.5 (**3**), 51.1 (**3^W**). **3^W/3** ratio (from ^{1}H NMR) = 3.0 (t_0), 14.0 (after 48h). In DMSO-d_6/DMEM-d (0.14/0.56 mL): **3^W /3** ratio (from ^{31}P NMR) = 0 (t_0), 1.9 (after 24h).

Figure S38. Complex [RuCl(κ^3-tpm)(PPh_3){NCHNHC(CH)$_4$C}]Cl, **4**, in D_2O

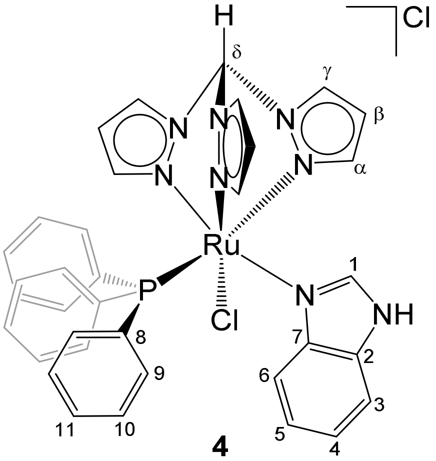

4

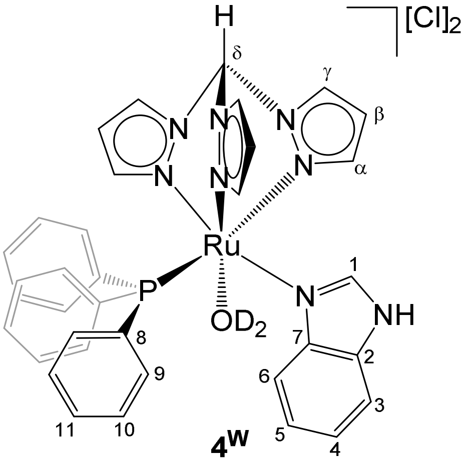

$\mathbf{4^W}$

^{1}H NMR (D_2O): δ/ppm (**4**) = 8.53-8.57 (m, 3H, $C^\gamma H$); 7.74-6.82 (m, 22H, $C^\alpha H$ + CH^{benzim}); 6.59, 6.35, 6.21 (t-br, 3H, $C^\beta H$); 5.38 (d-br, 1H, CH^{benzim}). δ/ppm ($\mathbf{4^W}$) = 8.53-8.57 (m, 3H, $C^\gamma H$); .74-6.82 (m, 22H, $C^\alpha H$ + CH^{benzim}); 6.46, 6.41, 6.08 (t-br, 3H, $C^\beta H$); 5.38 (d-br, 1H, CH^{benzim}). $C^\delta H$ *not observed.* $^{31}P\{^1H\}$ NMR (D_2O): δ/ppm = 52.2 ($\mathbf{4^W}$), 49.0 (**4**). $\mathbf{4^W/4}$ ratio (from ^{1}H NMR) = 2.5 (t_0), 2.5 (after 48h). In DMSO-d_6/DMEM-d (0.18/0.52 mL): $\mathbf{4^W/4}$ ratio.

Figure S39. Complex [RuCl(κ^3-tpm)(PPh$_3$){NNH(CH)$_3$}]Cl, **5**, in D_2O

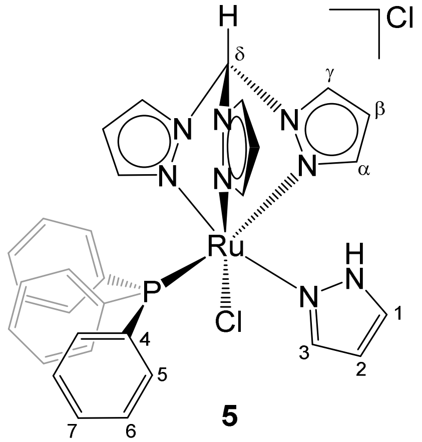

5

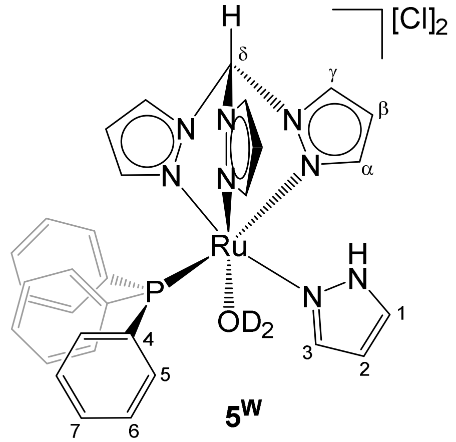

5^W

^{1}H NMR (D_2O): δ/ppm (**5**) = 8.37, 8.34, 8.31 (d-br, 3H, C^{γ}H); 7.60 (d-br, 1H, C^{α}H); 7.37 (t, 3H, C^{7}H); 7.37 (d-br, 1H); 7.18 (t, 6H, C^{5}H); 7.10 (d-br); 7.03 (d-br); 6.89-6.85 (m, 7H, C^{6}H + C^{1}H); 6.47, 6.34, 6.25, 6.11 (t-br, 4H, C^{2}H + C^{β}H). δ/ppm (**5^W**) = 8.41, 8.38, 8.37 (d-br, 3H, C^{γ}H); 7.72 (d-br, 1H, C^{α}H); 7.46 (t, 3H, C^{7}H); 7.41 (d-br, 1H); 7.27 (t, 6H, C^{5}H); 7.26 (d-br); 6.97 (d-br); 6.89-6.85 (m, 7H, C^{6}H + C^{1}H); 6.54, 6.42, 6.29, 6.15 (t-br, 4H, C^{2}H+ C^{β}H). C^{δ}H *not observed.* ^{31}P{^{1}H} NMR (D_2O): δ/ppm = 51.1 (**5^W**), 49.7 (**5**). **5^W/5** ratio (from ^{1}H NMR) = 0.3 (t_0), 7.4 (after 48h). In DMSO-d_6/DMEM-d (0.14/0.56 mL): **5^W/5** ratio (from ^{31}P NMR) = 0 (t_0), 1 (after 24h).

Figure S40. Complex [RuCl(κ^3-tpm)(PPh_3){N(CH)$_3$C(OH)CH}]Cl, **6**, in D_2O

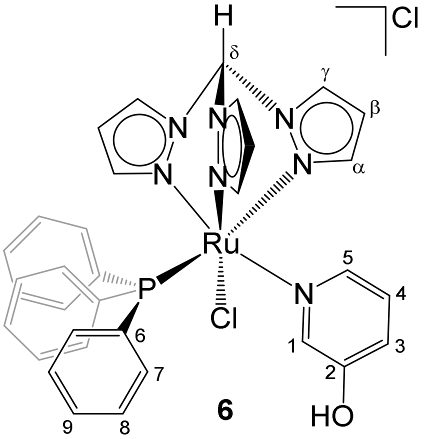

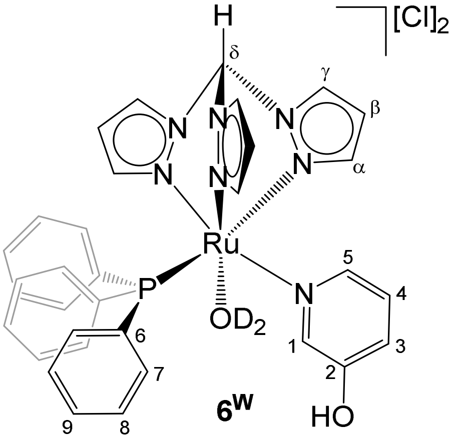

^{1}H NMR (D_2O): δ/ppm (**6**) = 9.53 (s-br, 1H, $C^{\delta}H$); 8.41, 8.37, 8.37 (d-br, 3H, $C^{\gamma}H$); 7.83 (s-br, 1H, C^5H); 7.5 (s-br, 1H, C^1H); 7.45 (m, 4H, $C^9H + C^3H$); 7.25 (m, 7H, $C^7H + C^{\alpha}H$); 7.06 (m, 2H, $C^{\alpha}H + C^4H$); 6.96 (m, 7H, $C^8H + C^{\alpha}H$); 6.54, 6.33, 6.15 (t-br, 3H, $C^{\beta}H$). *OH not observed.* δ/ppm (**6^W**) = 8.46, 8.42, 8.40 (d-br, 3H, $C^{\gamma}H$); 7.50-7.20 (m, 13H, $C^5H + C^1H + C^9H + C^3H + C^7H + C^{\alpha}H$); 6.99-6.93 (m, 9H, $C^{\alpha}H + C^4H + C^8H$); 6.58, 6.35, 6.18 (t-br, 3H, $C^{\beta}H$). *$C^{\delta}H$ and OH not observed.* $^{31}P\{^1H\}$ NMR (D_2O): δ/ppm = 51.2 (**6^W**), 50.0 (**6**). **6^W/6** ratio (from ^{1}H NMR) = 0.2 (t_0), *only B* (after 48h). In DMSO-d_6/DMEM-d (0.14/0.56 mL): **6^W/6** ratio (from ^{1}H NMR) = 0.1 (t_0), 2.3 (after 24h).

Figure S41. Complex [RuCl(κ^3-tpm)(PPh$_3$){P(OMe)$_3$}]Cl, **7**, in D$_2$O

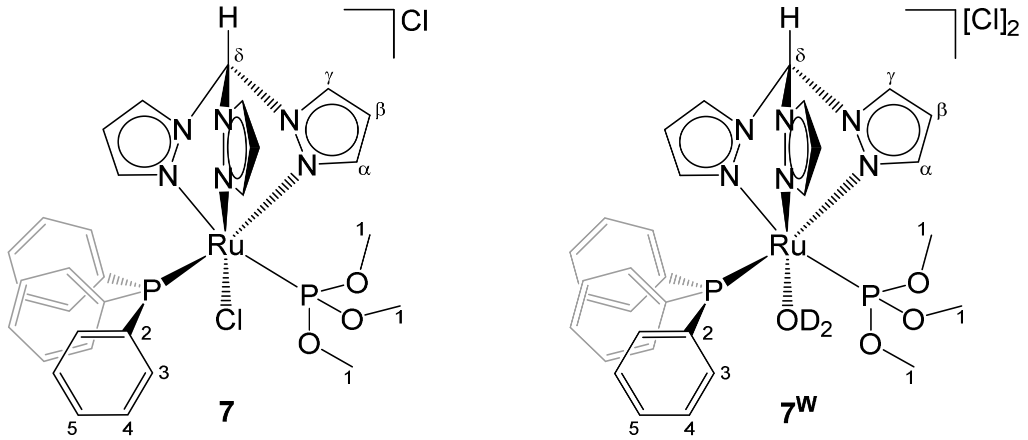

^{1}H NMR (D$_2$O): δ/ppm (**7**) = 8.30, 8.30, 8.28 (d-br, 3H, C^{γ}H); 8.24 (d-br, 1H, C^{α}H); 7.52-7.31 (m, 15H, C^{3}H + C^{4}H + C^{5}H); 8.83, 6.74 (d-br, 2H, C^{α}H); 6.74, 6.33, 6.30 (t-br, 3H, C^{β}H); 3.47 (d, $^3J_{HP}$ = 10.4 Hz, C^{1}H). δ/ppm (**7^W**) = 8.45, 8.44, 8.40 (d-br, 3H, C^{γ}H); 8.16 (d-br, 1H, C^{α}H); 7.52 (t-br, 3H, C^{5}H); 7.36-7.34 (m, 7H, C^{3}H + C^{α}H); 7.22 (t-br, 6H, C^{4}H); 8.98 (d-br, 1H, C^{α}H); 6.65, 6.33, 6.30 (t-br, 3H, C^{β}H); 3.39 (d, $^3J_{HP}$ = 10.4 Hz, C^{1}H). *C^{δ}H not observed.* ^{31}P{^{1}H} NMR (D$_2$O): δ/ppm = 136.2 (d, $^2J_{PP}$ = 56.4 Hz, P(OMe)$_3$), 133.4 (d, $^2J_{PP}$ = 62.6 Hz, P(OMe)$_3$), 44.67 (d, $^2J_{PP}$ = 57.1 Hz, PPh$_3$, **7**), 43.7 (d, $^2J_{PP}$ = 48.7 Hz, PPh$_3$, **7**). **7^W/7** ratio (from ^{1}H NMR) = 1.3 (t$_0$), 5.0 (after 48h). In DMSO-d$_6$/DMEM-d (0.14/0.56 mL): **7^W/7** ratio (from ^{1}H NMR) = 0 (t$_0$), 0.2 (after 24h).

Figure S42. Complex [RuCl(κ^3-tpm)(PPh_3)(CNCy)]Cl, **8**, in D_2O

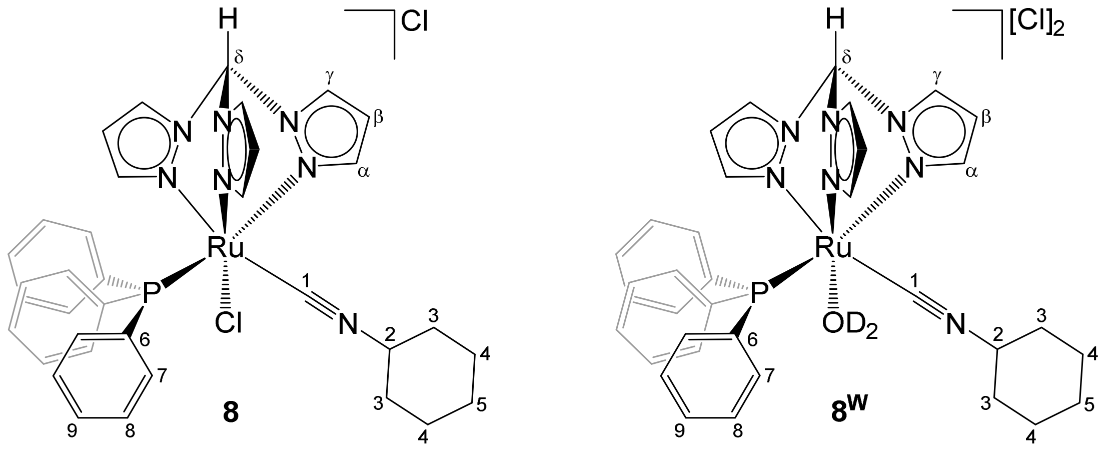

8 **8^W**

^{1}H NMR (D_2O): δ/ppm (**8**) = 8.36, 8.28, 8.28 (d-br, 3H, C$^\gamma$H); 8.17 (d-br, 1H, C$^\alpha$H); 7.43-7.54 (m, 15H, C^{7}H + C^{8}H + C^{9}H); 6.92, 6.52 (d-br, 2H, C$^\alpha$H); 6.59, 6.32, 6.13 (t-br, 3H, C$^\beta$H); 4.18 (m, 1H, C^{2}H); 1.86 (m, 2H, C^{4}H); 1.69 (m, 2H, C^{3}H); 1.51-1.30 (m, 6H, CH^{Cy}). δ/ppm (**8^W**) = 8.41, 8.34, 8.34 (d-br, 3H, C$^\gamma$H); 8.14 (d-br, 1H, C$^\alpha$H); 7.55 (t-br, 3H, C^{9}H); 7.42 (m, 6H, C^{7}H); 7.27 (t-br, 6H, C^{8}H); 7.11, 6.65 (d-br, 2H, C$^\alpha$H); 6.62, 6.34, 6.18 (t-br, 3H, C$^\beta$H); 4.17 (m, 1H, C^{2}H); 1.85 (m, 2H, C^{4}H); 1.67 (m, 2H, C^{3}H); 1.40-1.26 (m, 6H, C-H^{Cy}). *C$^\delta$H not observed.* ^{31}P{^{1}H} NMR (D_2O): δ/ppm = 48.1 (**8**), 47.5 (**8^W**). **8^W/8** ratio (from ^{1}H NMR) = 0.5 (t_0), 9.1 (after 48h). In DMSO-d_6/DMEM-d (0.14/0.56 mL): **8^W/8** ratio (from ^{1}H NMR) = 0 (t_0), 0.2 (after 24h).

Figure S43. Complex [RuCl(κ^3-tpm)(PPh$_3$){CNCH$_2$P(O)(OEt$_2$)$_2$}]Cl, **9**, in D$_2$O

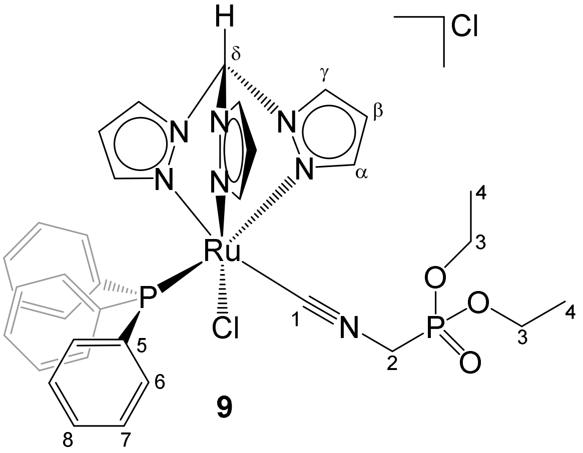

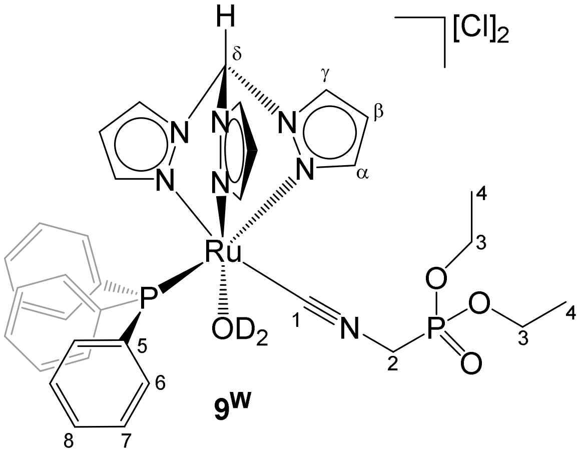

^{1}H NMR (D$_2$O): δ/ppm (**9**) = 8.40, 8.32, 8.32 (d-br, 3H, C^{γ}H); 8.18 (d-br, 1H, C^{α}H); 7.36 (m, 9H, C^{6}H + C^{8}H); 7.23 (t-br, 6H, C^{7}H); 6.87, 6.49 (d-br, 2H, C^{α}H); 6.61, 6.22, 6.15 (t-br, 3H, C^{β}H); 4.79 (m, 2H, C^{2}H, *D$_2$O superimposed*); 3.98 (m, 4H, C^{3}H); 0.98 (m, 6H, C^{4}H). δ/ppm (**9^W**) = 8.48, 8.42, 8.38 (d-br, 3H, C^{γ}H); 8.19 (d-br, 1H, C^{α}H); 7.62 (t-br, 3H, C^{8}H); 7.47 (t-br, 6H, C^{6}H or C^{7}H); 7.31 (t-br, 6H, C^{6}H or C^{7}H); 7.16, 6.65 (d-br, 2H, C^{α}H); 6.65, 6.39, 6.26 (t-br, 3H, C^{β}H); 4.79 (m, 2H, C^{2}H, *D$_2$O superimposed*); 4.20 (m, 4H, C^{3}H); 1.36 (t, 6H, $^3J_{HH}$ = 7.1 Hz, C^{4}H). C^{δ}H *not observed.* ^{31}P{^{1}H} NMR (D$_2$O): δ/ppm = 46.4 (s, PPh$_3$); 17.3 (s, PCH$_2$, **9**), 40.6 (s, PPh$_3$); 29.9 (s, PCH$_2$, **9^W**). **9^W/9** ratio (from ^{31}P{^{1}H} NMR) = 0 (t$_0$), 2.8 (after 48h). In DMSO-d$_6$/DMEM-d (0.14/0.56 mL): **9^W/9** ratio (from ^{1}H NMR) = 0 (t$_0$), 0.3 (after 24h).

Figure S44. ^{1}H NMR spectrum (301 MHz, D_2O) of **3** + **3**^{W} (see Figure S37) at t_0 (blue) and after 48h (red).

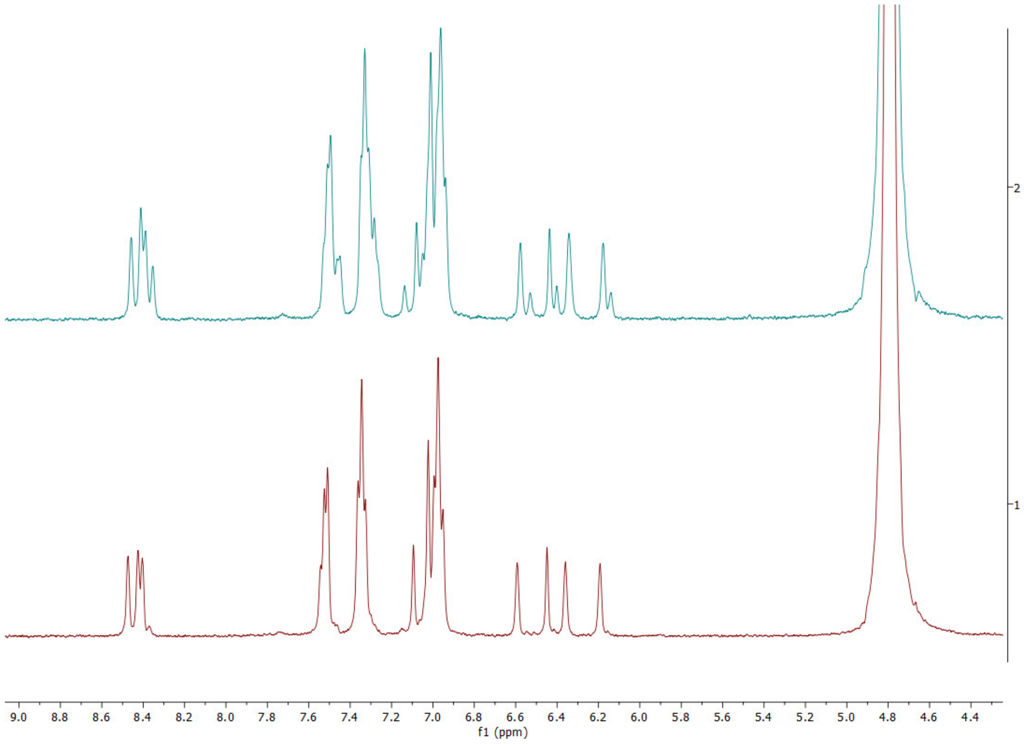

Figure S45. ^{31}P NMR spectrum (121 MHz, D_2O) of **3** + **3**^{W} (see Figure S37) at t_0 (blue) and after 48h (red).

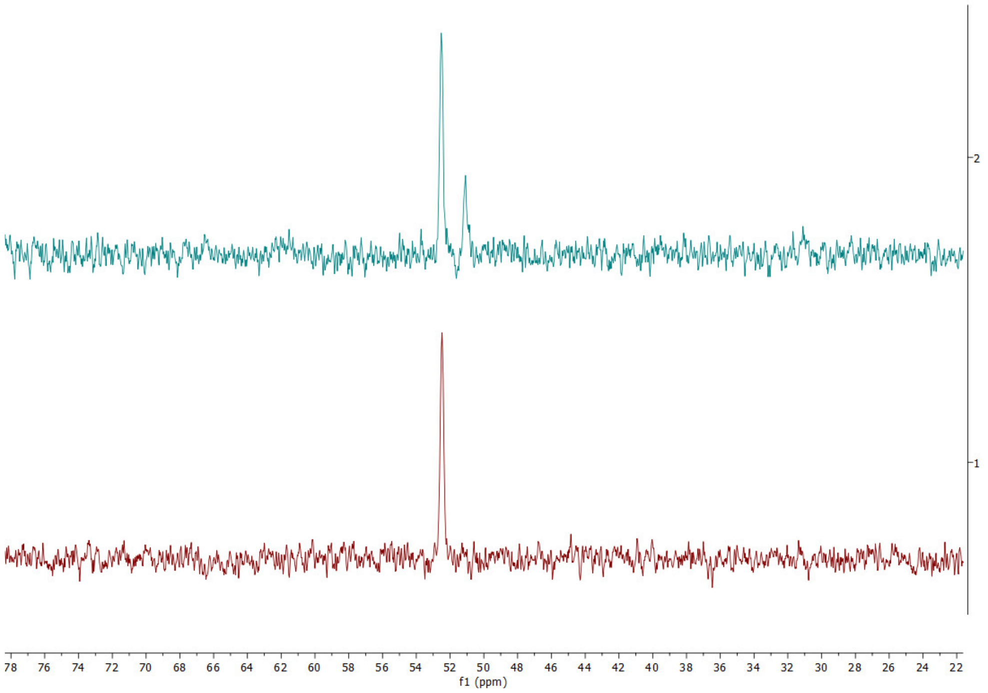

Figure S46. ^{1}H NMR spectrum (501 MHz, D_2O) of **3** immediately after the preparation of the sample (only **3** is observed).

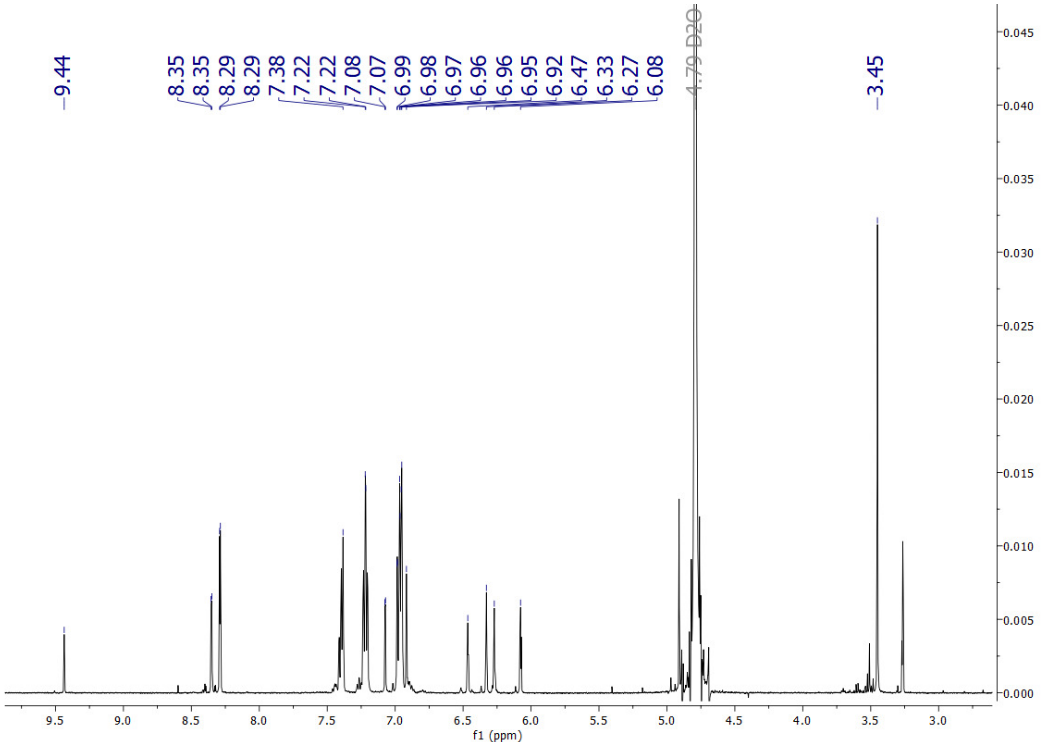

Figure S47. ^{31}P NMR spectrum (202 MHz, D_2O) of **3** immediately after the preparation of the sample (only **3** is observed).

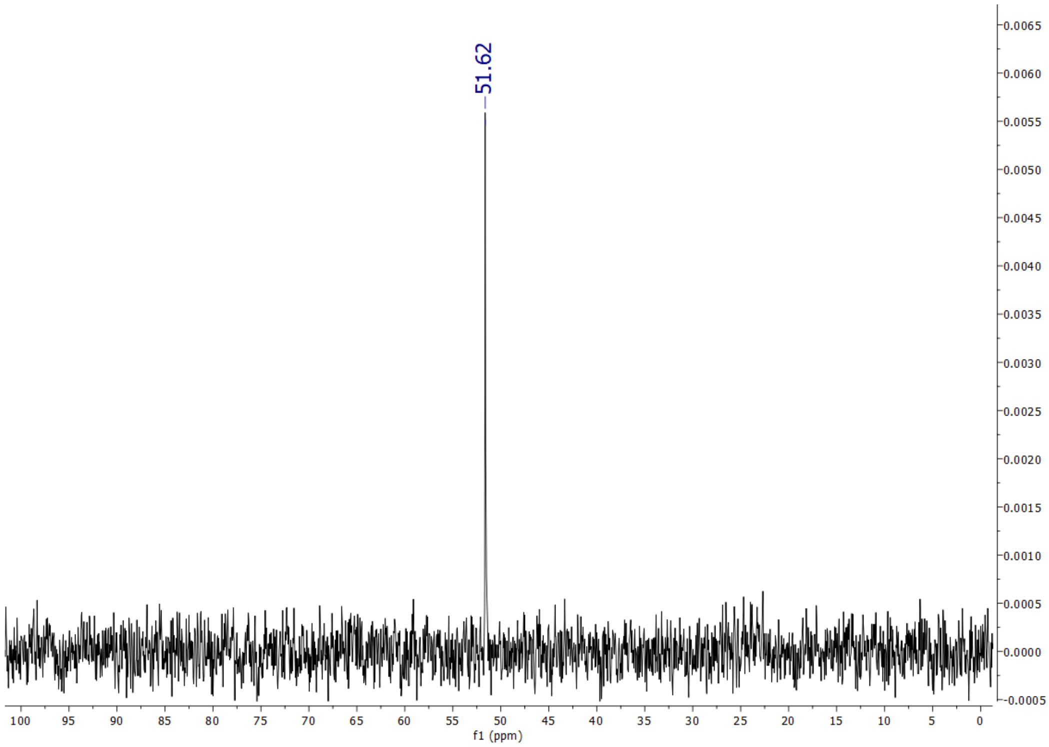

Experiments on chloride/water exchange. Compound **3** was dissolved in H_2O and maintained at 40 °C for 24h. The solution was then cooled to room temperature, the solvent was evaporated, and the obtained solid was washed with diethyl ether (2 x 3 mL). The isolated yellow powder was dissolved in CD_3OD, then ^{1}H NMR and ^{31}P NMR spectra of the solution were recorded immediately after the preparation of the sample (red), and after 24h at room temperature (green). Both **3** and $\mathbf{3^W}$ (see Figure S37) were identified in the first recorded spectrum. After 24h, only **3** was detected (see Figures S49-S50). ^{1}H NMR (CD_3OD): δ/ppm = 9.68 (s, 1H, $C^{\delta}H$); 8.47, 8.46, 8.40 (d, 3H, $^{3}J_{HH}$ = 2.9 Hz, $C^{\gamma}H$); 7.44 (d-br, 1H, $C^{\alpha}H$); 7.41 (t, 3H, $^{3}J_{HH}$ = 7.4 Hz, $C^{7}H$); 7.24 (t, 6H, $^{3}J_{HH}$ = 7.8 Hz, $C^{5}H$); 7.14 (m, 2H, $C^{\alpha}H + CH^{Imid}$); 7.06 (t-br, 6H, $C^{6}H$); 7.02 (d-br, 1H, $C^{\alpha}H$); 6.99 (t-br, 1H, CH^{Imid}); 6.52, 6.35, 6.20 (t-br, 1H, $C^{\beta}H$); 6.26 (t-br, 1H, CH^{Imid}); 3.50 (s, 3H, $C^{4}H$). $^{31}P\{^{1}H\}$ NMR (CD_3OD): δ/ppm = 51.1.

Figure S48. ^{1}H NMR spectra (301 MHz) of **3** in CD_3OD solution. Red: from isolated solid following chloride/water exchange in H_2O at 40 °C; green: same sample maintained in CD_3OD solution at room temperature for 24h; blue: from **3** not undergoing chloride/water exchange.

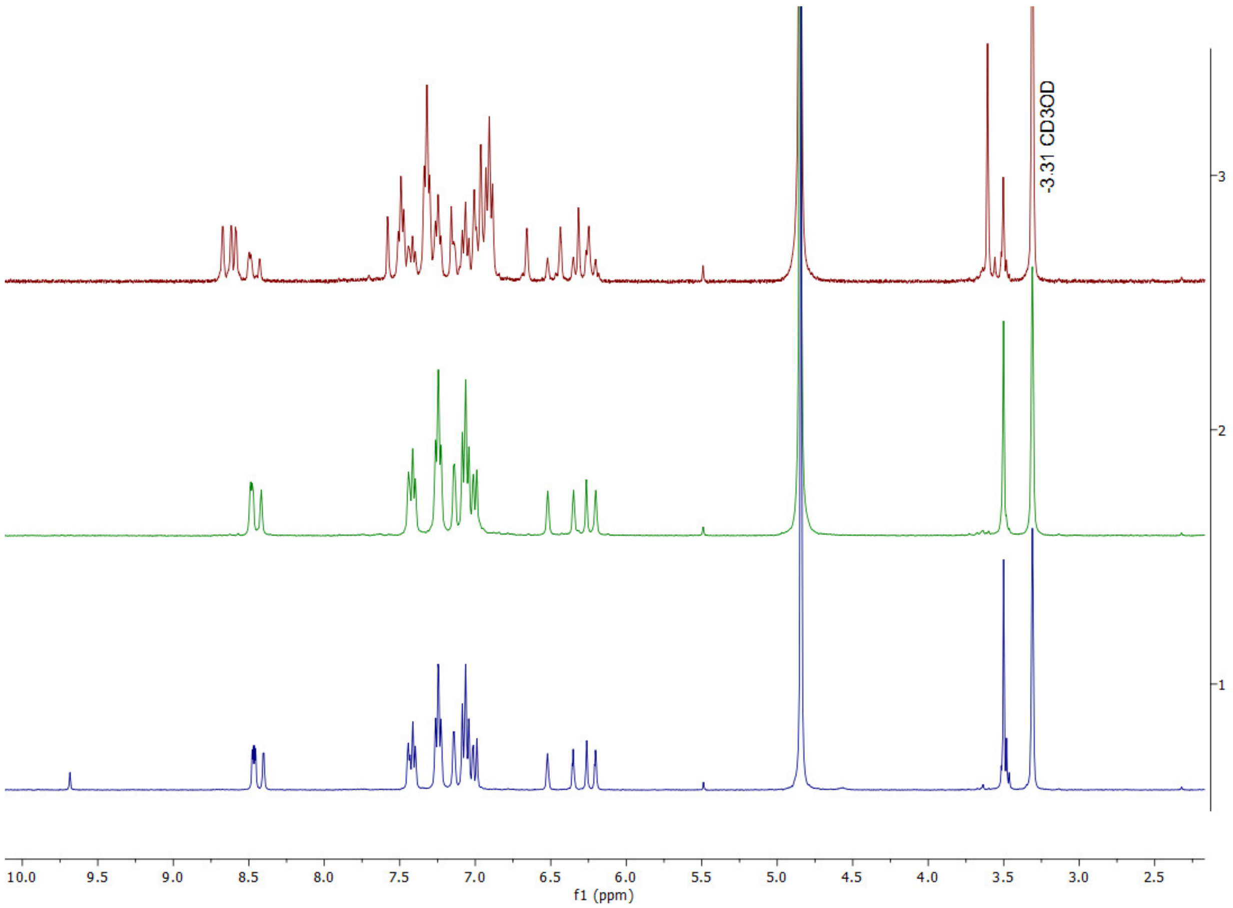

Figure S49. ^{31}P NMR spectra (121 MHz, CD_3OD) of **3** in CD_3OD solution. Red: from isolated solid following chloride/water exchange in H_2O at 40 °C; green: same sample maintained in CD_3OD solution at room temperature for 24h; blue: from **3** not undergoing chloride/water exchange.

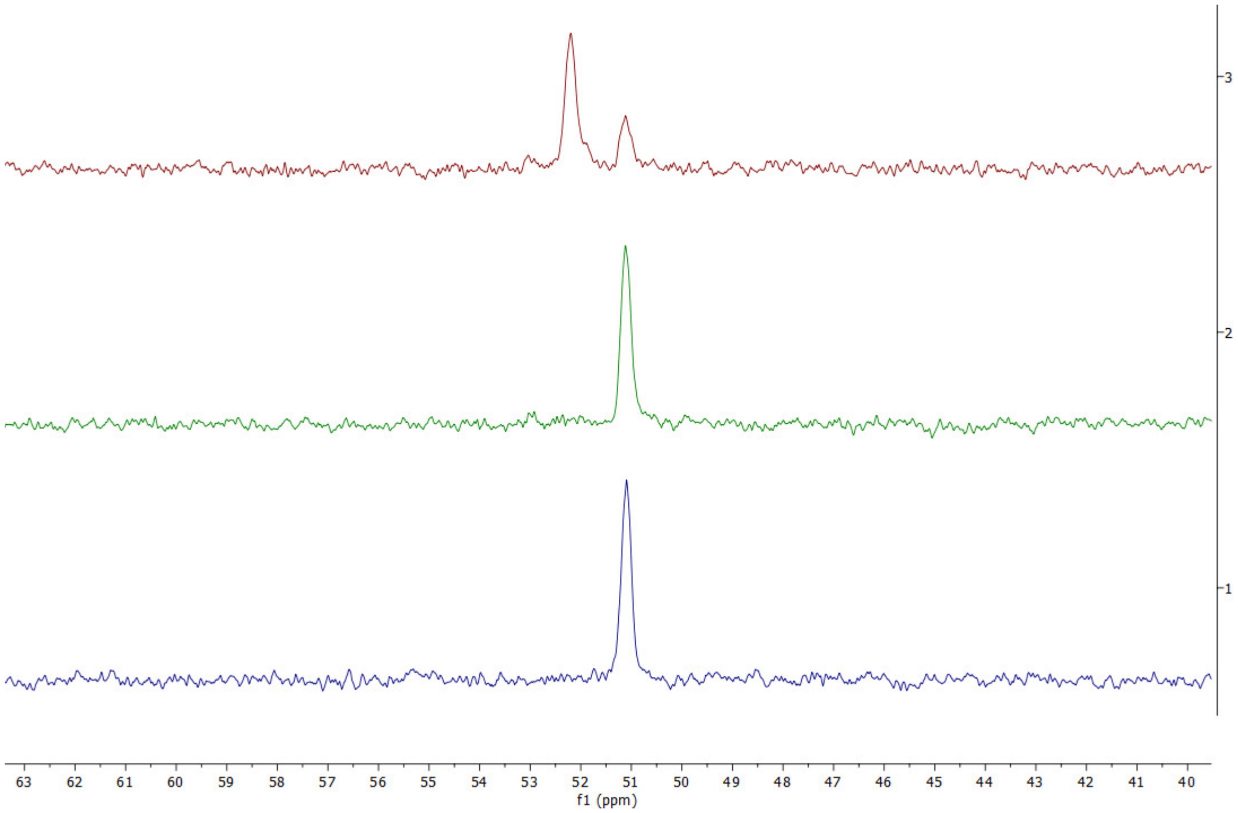

Figure S50. ^{31}P NMR spectrum (121 MHz, DMSO-d_6/DMEM-d 1:4 v/v) of **2**, at t_0 (blue) and after 24h at 37 °C (red).

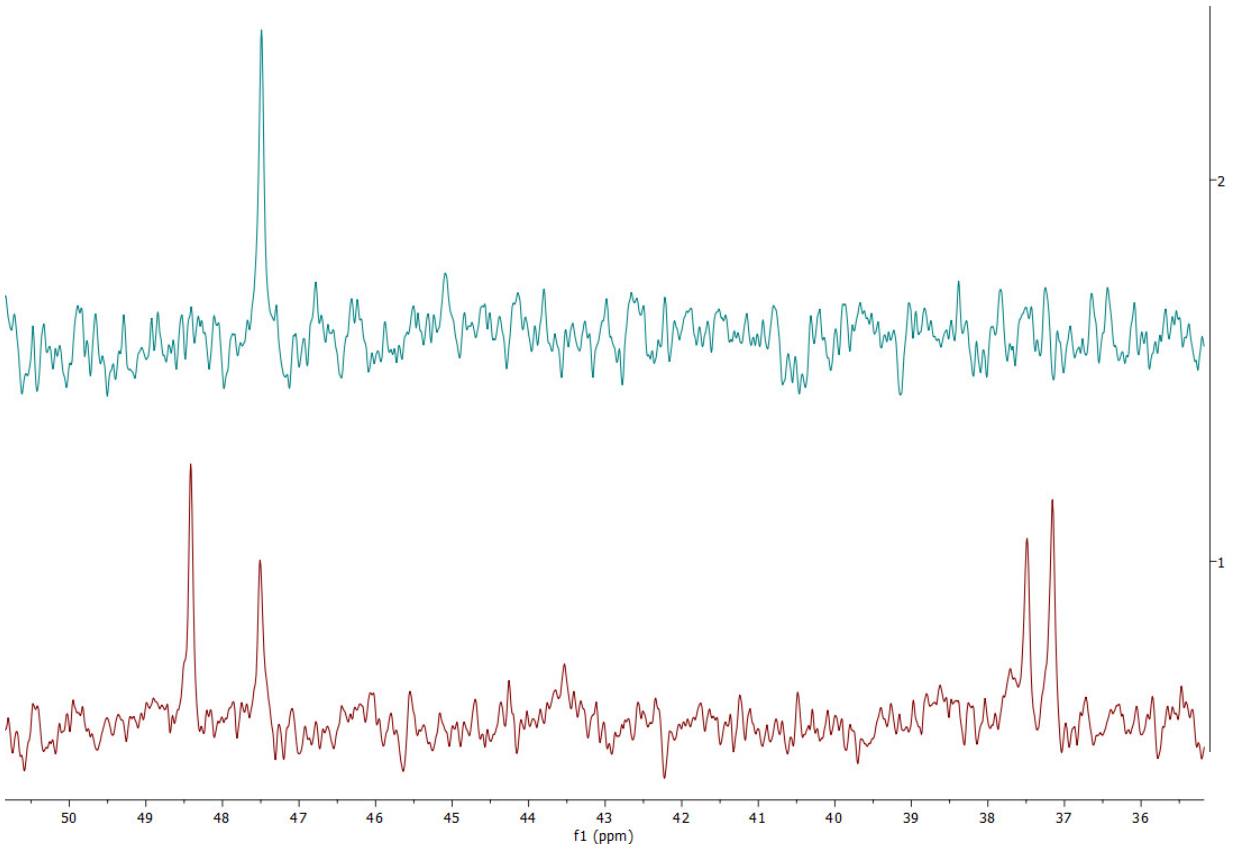

Table S1. Relative amounts of aquo-complexes in D_2O at t_0 and after 48h at 37 °C; relative amounts of aquo-complexes in DMSO-d_6-DMEM-d (1:4 v/v, except 1:3 v/v in the case of **4**) at t_0 (see manuscript) and after 24h at 37 °C; average of IC_{50} values related to cancer cell lines; Log P_{ow} values (see also Table 2). Data of **1** are not reported because the aquo-species was not observed in this case.

Compound	Aquo species % in D_2O at t_0	Aquo species % in D_2O after 48h	Aquo species % in DMEM-d/ DMSO-d_6 at t_0	Aquo species % in DMEM-d/ DMSO-d_6 after 24h	Average IC_{50}	Log P_{ow}
**1**	-	-	-	-	2.5 ± 0.3	1.18 ± 0.05
**2**	80	92	0	59	32.4 ± 3.8	–0.33 ± 0.07
**3**	75	93	0	66	42.8 ± 4.2	–0.15 ± 0.03
**4**	67	70	0	59	31.6 ± 3.4	0.58 ± 0.06
**5**	25	88	0	50	34.0 ± 3.6	–0.05 ± 0.05
**6**	15	99	9	70	41.4 ± 7.4	1.11 ± 0.07
**7**	57	83	0	16	7.1 ± 1.0	–0.02 ± 0.05
**8**	33	90	0	16	9.9 ± 1.5	0.34 ± 0.01
**9**	0	73	0	17	35.2 ± 5.0	–0.20 ± 0.05

Table S2. IC_{50} values (μM) for HCT116 cell line determined by the SRB and MTT tests after 72 h of treatment.[a]

	SRB	MTT
**1**	0.45 ± 0.05	1.5 ± 0.1
**2**	16 ± 1	25 ± 2
**3**	16 ± 2	25 ± 1
**4**	21 ± 3	25 ± 2
**5**	20 ± 3	30 ± 2
**6**	20 ± 2	31 ± 2
**7**	5.8 ± 0.6	6.7 ± 0.4
**8**	5.2 ± 0.5	8 ± 2
**9**	29 ± 3	35 ± 6

[a]Data represent a mean ± SD from at least three independent experiments.

Figure S51. Comparative linear relationship between IC_{50} in HCT116 cells and amount of Ru taken up to the cells.

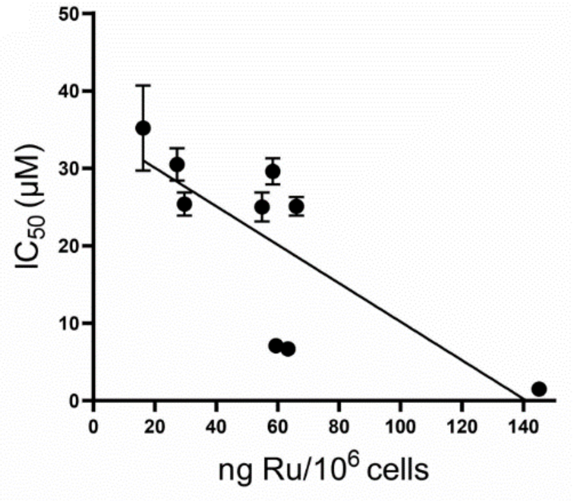

Figure S52. Evaluation of cell death by apoptosis quantified by FACS after Annexin V and PI staining. Density plot profiles of untreated HCT116 cells (K) or treated with staurosporine (Staur, 10 µM), ethanol (EtOH, 5% v/v) and Ru complexes **1** (6 µM), **7** (30 µM), **8** (20 µM) for 24 h. Concentrations of Ru complexes correspond to $4xIC_{50,72h}$.

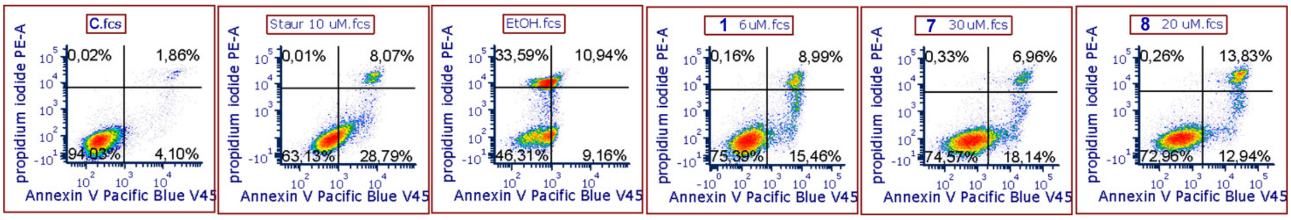

Figure S53. The kinetics of apoptosis and necrosis immediately after treatment of HCT116 cells with **1**, **7**, and **8** at their equitoxic concentrations (4x$IC_{50,72h}$). Differences of time when signals cross baseline were calculated and are indicative of cell death type. Positive controls staurosporine and EtOH, known apoptosis and necrosis inducers, respectively, were also included. The necrosis signal precedes the signal related to apoptosis for the EtOH treatment. For the staurosporine and all three tested Ru-complexes, the apoptotic signal is manifested significantly (3.6-13.6 h) earlier than the signal indicating necrosis.

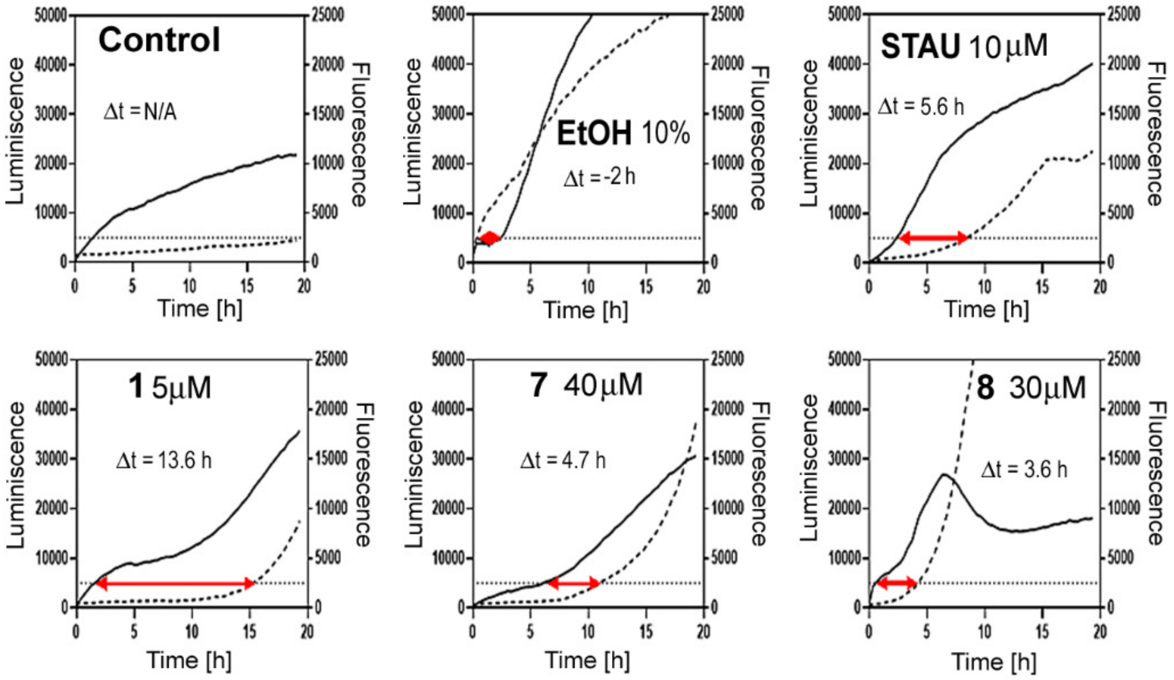

Table S3. IC_{50} values (μM) determined by the MTT test after 72 h of treatment.[a]

	CHO-K1	MMC-2
**1**	6.3 ± 0.2	7.7 ± 0.2
**7**	25.6 ± 0.5	33 ± 2
**8**	16 ± 4	16 ± 3
cisplatin	54 ± 6	5.5 ± 0.8

[a]The results are expressed as mean values: SD from at least three independent experiments.

Figure S54. Immunofluorescent staining of tubulin in HCT116 cells either untreated (control) or treated with complexes **1**, **7** or **8**. The primary antibody (anti-α-Tubulin, Abcam, 1:200 dilution, 1 h) and Alexa Fluor conjugated secondary antibody (Goat Anti-Rabbit, Abcam, 1:500 dilution, 1 h) were used to visualize α-tubulin. DAPI was used to stain the cell nuclei (blue). Scale bar represents 20 μm.

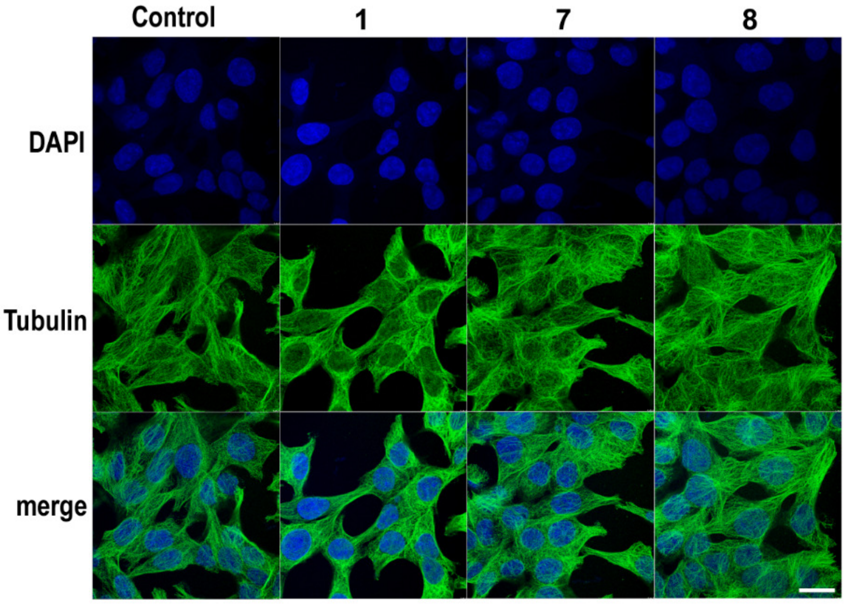

Figure S55. Immunofluorescent staining of actin in HCT116 cells either untreated (control) or treated with complexes **1**, **7** or **8**. Actin was stained with Alexa Fluor 488 conjugated Phalloidin (ThermoFisher Scientific, 1:50 dilution, 20 min). DAPI was used to stain the cell nuclei (blue). Scale bar represents 20 μm.

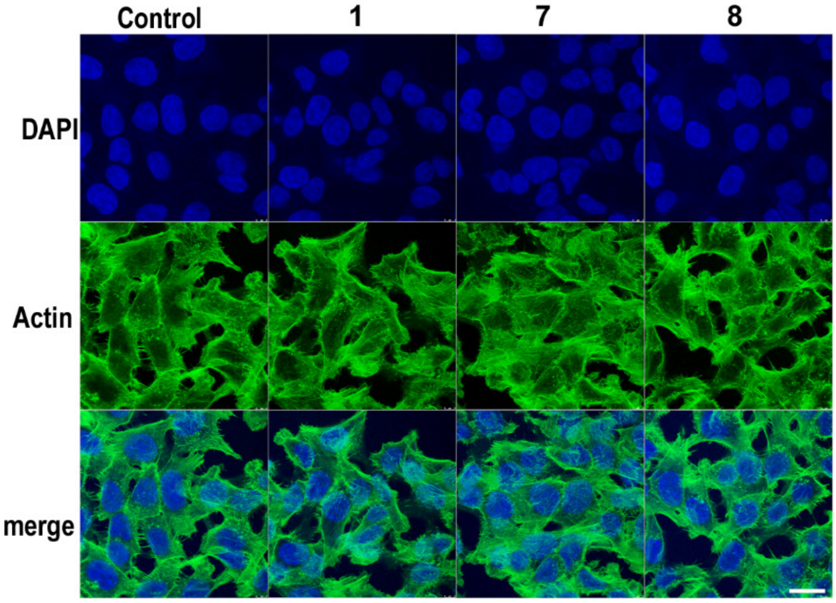

Figure S56. Representative TMRE histograms. HCT116 cells were treated for 5 h with equitoxic concentrations corresponding to 4xIC_{50} values (panel A) or treated with Ru complexes at the equimolar concentration (10 µM) (panel B).

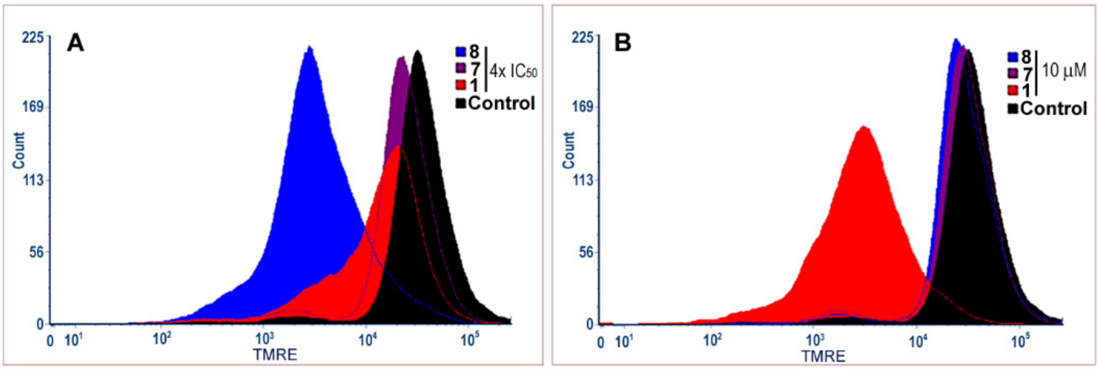

Figure S57. Representative bright-field images of HCT116 colonospheres. Four-day spheroids were either untreated (control, panel A) or treated with **1** (B), **7** (C), **8**(D) or cisplatin (E) at their 20 µM concentrations and incubated for a further 72 h. Scale bar represents 100 µm.

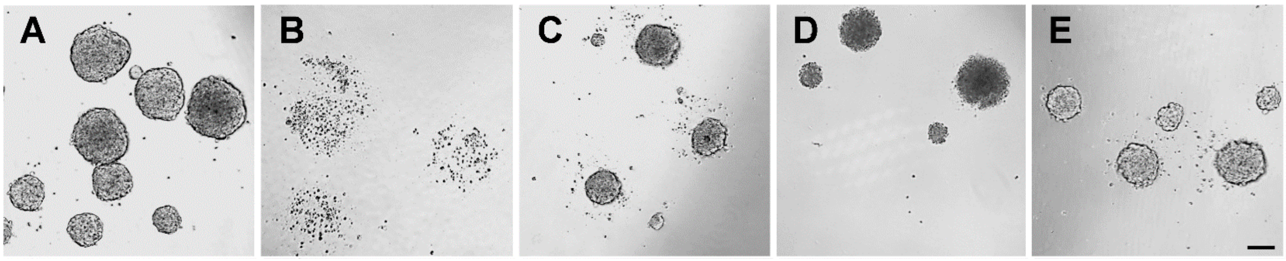

Figure S58. HR-ESI-MS analysis of **9**. Top: FIA-ESI-Q-ToF mass spectrum after blank subtraction (10 ppm solution in acetonitrile, HPLC-MS grade); bottom: zoomed portion of the spectrum highlighting the isotopic cluster of the ion corresponding to the cation of the analyzed complex; the red rectangles mark the expected m/z values and intensities calculated for the formula $[C_{34}H_{37}ClN_7O_3P_2Ru]^+$.

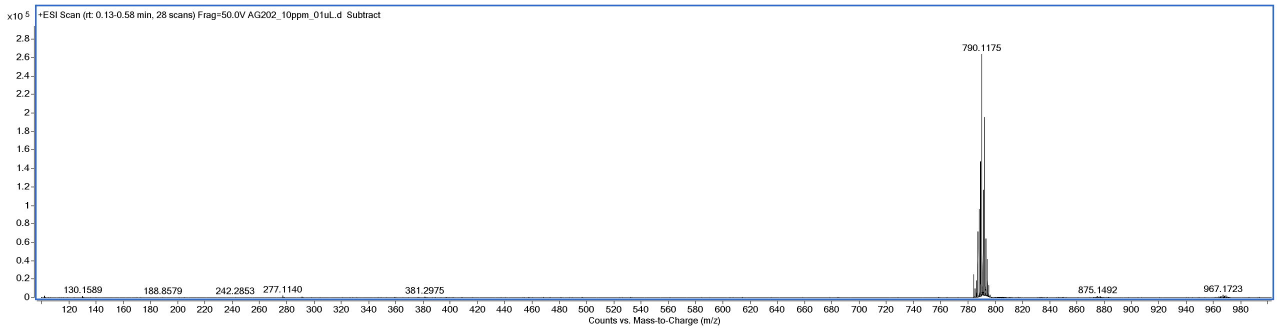

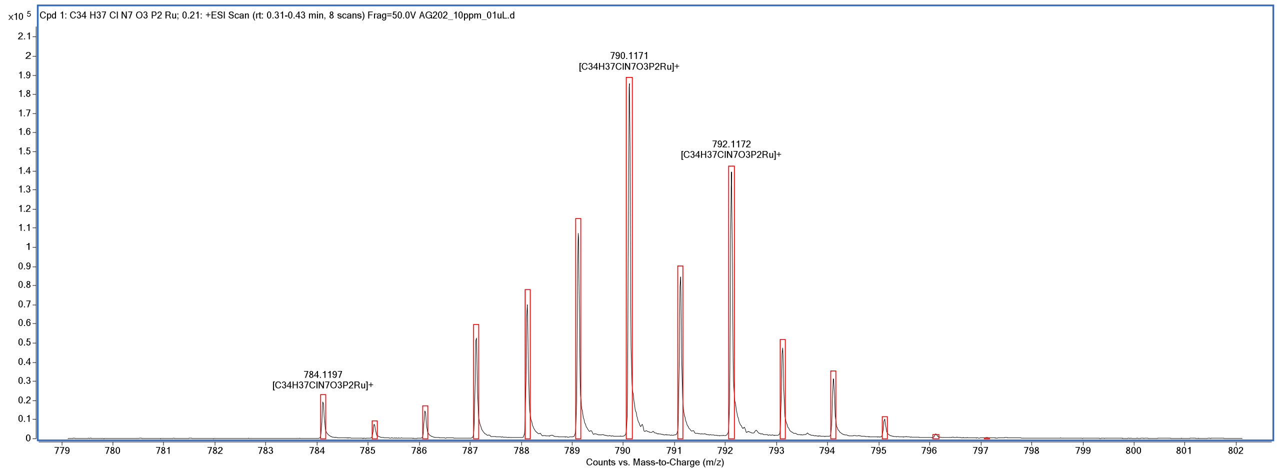

Supplement: Supplementary file 1 — jm2c00722_si_001.pdf [file jm2c00722_si_001.pdf]
